# Association between Heavy metals and triglyceride-glucose-related index: a mediation analysis of inflammation indicators

**DOI:** 10.1186/s12944-025-02441-9

**Published:** 2025-02-13

**Authors:** Yitao Hu, Yuzhe Kong, Xinling Tian, Xinyi Zhang, Yu Zuo

**Affiliations:** 1 Xiangya School of Medicine, Central South University, Changsha, China; 2 College of Education, Wenzhou University, Wenzhou, China; 3 Third Xiangya Hospital of Central South University, Changsha, China

**Keywords:** Heavy metal, Inflammation, Triglyceride, Glucose Index, Triglyceride, Glucose Index and Waist Circumference, Triglyceride, Glucose Index and Waist Height Ratio, Triglyceride, Glucose Index and Body Mass Index, Mediation analysis, National Health and Nutrition Examination Survey

## Abstract

**Background:**

In cardiovascular diseases (CVD) and insulin resistance (IR), elevated blood lipids and glucose are common. These abnormalities accelerate atherosclerosis and may impair insulin signaling via oxidative stress and inflammation. The triglyceride-glucose (TyG) index is a cost-effective marker for assessing IR and CVD risk, reflecting insulin resistance and early atherosclerosis. However, research on factors affecting the TyG index, especially mixed heavy metal exposure, is limited. Heavy metals might alter the TyG index by inducing oxidative stress and inflammation, affecting lipid and glucose metabolism. This study explores the link between heavy metal exposure and TyG index changes, focusing on inflammation's mediating role, aiming to offer new strategies for CVD and IR prevention and management.

**Method:**

This research explores the association between heavy metal concentrations and TyG indicators, drawing on data from the National Health and Nutrition Examination Survey spanning 2011 to 2016. It employs a range of statistical approaches, such as linear and non-linear analyses, multiple linear regression, weighted quantile sum regression, and Bayesian kernel machine regression. Additionally, a mediation analysis investigates the role of inflammation in modifying the effects of heavy metal exposure.

**Result:**

The research analyzed data from a sample of 2,050 individuals, finding notable links between mixed heavy metals and variations in TyG markers. Specifically, the presence of heavy metal mixtures was associated with significant increases in these indicators. Additionally, six inflammatory markers were identified that act as intermediaries in the process leading from heavy metal exposure to alterations in TyG indicators.

**Conclusion:**

The study establishes a clear association between heavy metal and adverse changes in TyG markers, influenced in part by inflammation. These insights highlight the urgent need for improved monitoring of environmental health and specific strategies to decrease heavy metal exposure, thus lessening their harmful impact on cardiovascular health. The research enhances understanding of the dynamic interactions between environmental exposures and metabolic health, laying groundwork for public health initiatives aimed at curtailing chronic disease risks linked to heavy metals.

**Graphical Abstract:**

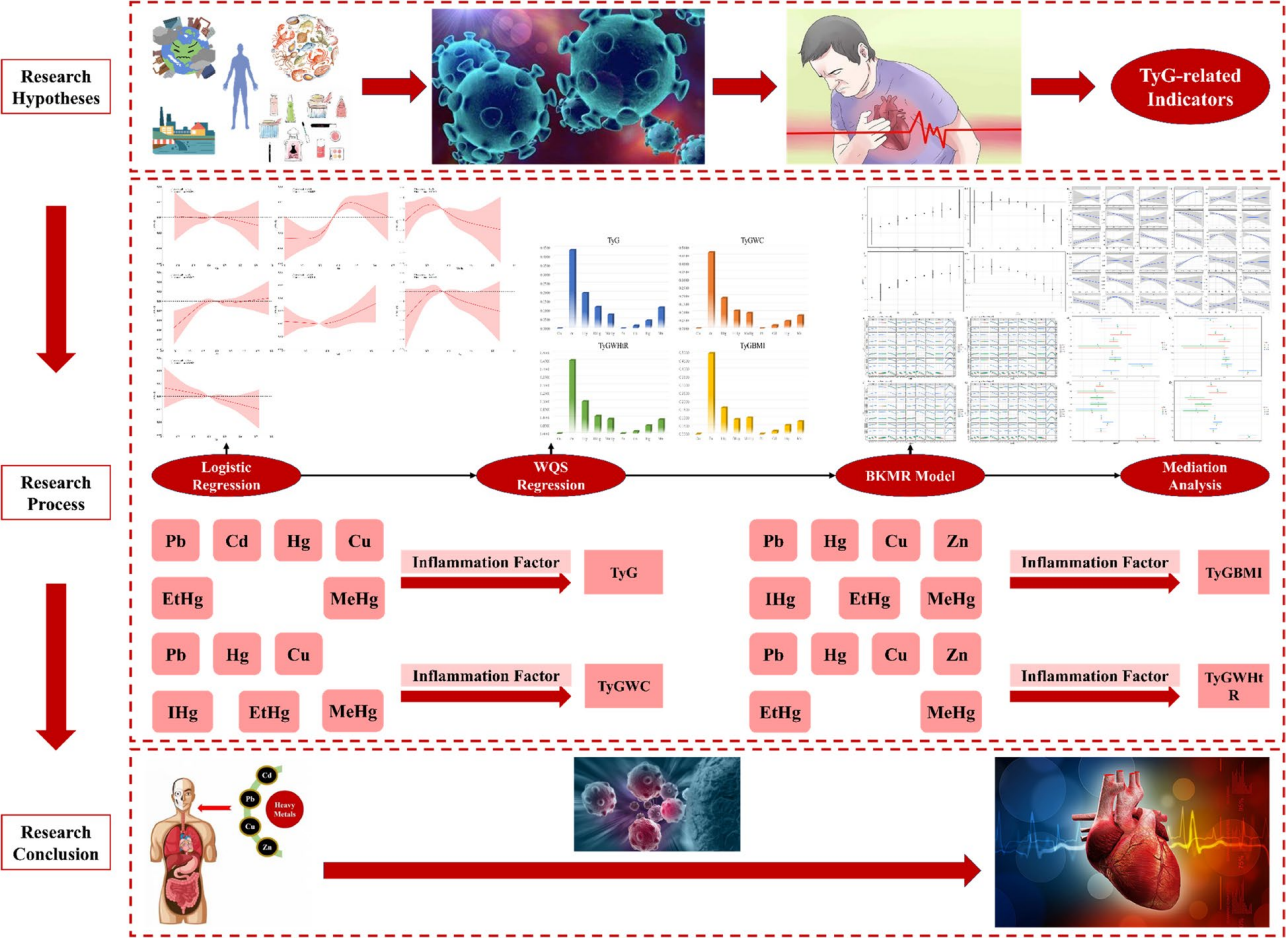

**Supplementary Information:**

The online version contains supplementary material available at 10.1186/s12944-025-02441-9.

## Introduction

Cardiovascular diseases (CVDs) significantly impact global health, leading to numerous complications and roughly 17.9 million deaths each year, making up about 31% of all worldwide fatalities [1, 2]. An increase in CVD incidence is anticipated due to factors like aging populations, urbanization, and unhealthy lifestyle choices [3, 4]. Estimates predict that by 2030, CVDs will cause over 23 million deaths annually, exacerbating the public health crisis [5].

Evidence increasingly links blood heavy metals to a higher risk of CVDs, with multiple studies based on NHANES data confirming a strong positive correlation between these metals and CVD occurrence [6–9]. Additionally, a Korean study identified a significant relationship between blood levels of lead (Pb) and cadmium (Cd) and the TyG index in males [10]. Recent insights into CVD mechanisms suggest that heavy metals, by competing with essential metals and damaging vascular endothelial cells through oxidative stress, inflammation, and endocrine disruption, contribute to cardiovascular issues [11–13].

Inflammatory markers are recognized as key contributors to cardiovascular disease [14], with research demonstrating their link to elevated mortality risks from these conditions [15]. Elevated inflammatory marker levels generally suggest a systemic metabolic imbalance, potentially heightening the risk of cardiovascular problems through various biological mechanisms [16–18]. According to Blake's research, inflammatory processes, particularly TNF-α and CRP, are pivotal throughout the progression of atherosclerosis, from the initial adherence of leukocytes to arterial walls to the rupture of plaques [19], highlighting a key underlying mechanism.

Studies also illustrate the critical role heavy metals have in stimulating inflammatory responses. Anka's research demonstrated that heavy metals might compromise the immune system by reducing the effectiveness and quantity of immune cells [20]. Further findings indicate that Pd disturbs the equilibrium between pro-oxidants and antioxidants, leading to oxidative stress and modifying the balance of oxidative and antioxidant reactions [21, 22]. This imbalance encourages the production of inflammatory mediators, activates inflammatory pathways, and impacts cytokine metabolism and expression, ultimately influencing the activity of inflammatory enzymes.

Recent research highlights a strong association between TyG-related markers and the onset of cardiovascular diseases. Various cohort studies have demonstrated that a higher TyG index is linked to an elevated risk of cardiovascular disease [23–29]. This suggests the potential for developing a non-invasive, cost-effective method to assess and prevent cardiovascular disease using TyG-related markers. However, previous research primarily examined how blood heavy metals and inflammatory markers directly cause cardiovascular disease, without connecting these factors to trends in TyG-related indicators [25, 26].

This study, therefore, investigates the associations between heavy metals and TyG-related markers, as well as the role of inflammatory factors as mediators, using a large-scale cross-sectional analysis of the NHANES dataset spanning from 2011 to 2016.

## Method

### Study population

Administered by the National Center for Health Statistics (NCHS) under the CDC, the NHANES program conducts biennial surveys to evaluate the health and dietary habits of non-institutionalized U.S. civilians. This survey utilizes a sophisticated four-stage, multiyear sampling process that targets a stratified and clustered demographic sample, selecting individuals according to factors such as age, sex, race/ethnicity, and socio-economic status. Results from these surveys are updated every two years.

### Ethic Approval

Informed consent was obtained from all participants. Ethic approval received from NCHS Ethics Review Board (Protocol #2011–17).

### Eligibility Cretia

**Fig. 1:**
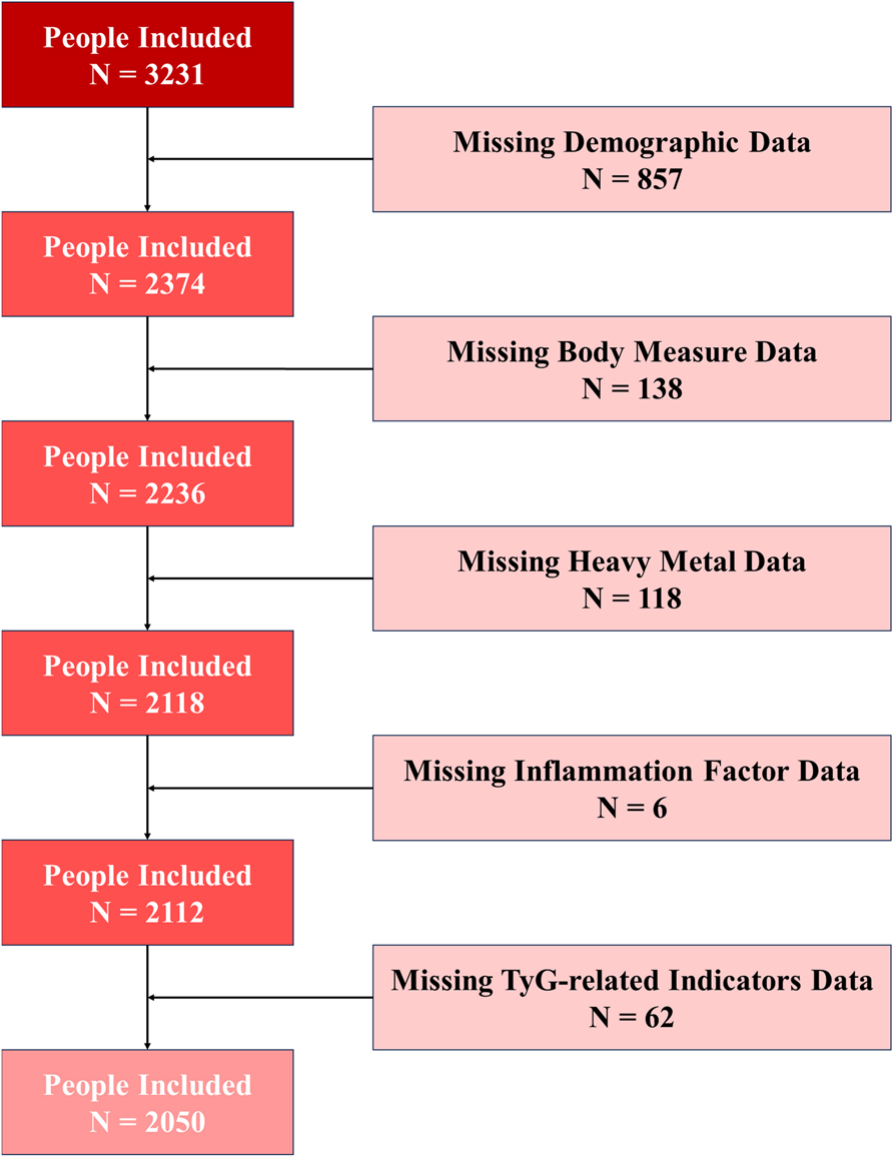
Study Flowchart

A total of 3231 participants with reliable NHANE data from 2011 to 2016 constituted the initial sample. 1181 participants were excluded because of various data missing. Eventually, 2050 participants were included in final analysis. (Fig. 1).

### Exposure Assessment

Cu and Zn concentrations were measured using inductively coupled plasma dynamic reaction cell mass spectrometry (ICP-DRC-MS), which precisely detects trace elements and isotopes like zinc, copper, and selenium. A standard calibration with gallium was employed, and serum samples were diluted for analysis standardization [30, 31].

For metals such as Cd, Mn, Hg, and Pb found primarily in red blood cells, blood samples were mixed thoroughly after adding EDTA to prevent clotting and ensure uniform distribution. The samples were then diluted and treated to release metals for accurate ICP-DRC-MS analysis, using specific internal standards for precision.

Mercury types, including inorganic and organic species, were differentiated and quantified using gas chromatography coupled with ICP-DRC-MS, which is critical for assessing mercury-related neuro and renal toxicity [29, 30].

### Outcome Assessment

The indexes were calculated by the following formula [32–34].

TyG = $$\text{ln}\frac{Blood Glucose \times Triglyceride}{2}$$;

TyG-WC = $$TyG \times Waist$$;

TyG-WHtR = $$TyG \times \frac{Waist}{Height}$$;

TyG-BMI = $$TyG \times BMI Index$$.

TyG Index: Utilized as a straightforward marker, combines triglyceride and fasting glucose levels. It's recognized for its broad applicability in assessing cardiovascular disease (CVD) risk.

TyG-WC (TyG and Waist Circumference): Integrates waist circumference into the TyG index, providing insights into how abdominal obesity interacts with lipid and glucose metabolism concerning CVD risk.

TyG-WHtR (TyG and Waist Height Ratio): Offers a nuanced perspective by adjusting for body proportion, which may be particularly beneficial in populations where height significantly influences health outcomes. It's believed that this ratio provides a more comprehensive assessment of CVD risk in relation to body shape.

TyG-BMI (TyG and Body Mass Index): Combines the TyG index with body mass index, offering an overview of general body adiposity rather than just abdominal. This index helps in examining the broad combined effects of overall obesity and lipidglucose metabolism on cardiovascular health outcomes.

### Covariates

This investigation incorporated a variety of significant clinical covariates identified from prior studies [35, 36]. These included the age of participants at the time of the interview, their sex, racial and ethnic backgrounds, levels of education, marital status, family income to poverty ratio (PIR), and key body measurements like weight, height, waist circumference, and body mass index (BMI).

NHANES categorizes racial and ethnic identities into several groups such as Mexican American, other Hispanic, non-Hispanic white, non-Hispanic black, non-Hispanic Asians, and multi-racial individuals. Education is classified from below ninth grade to college graduate or higher. Marital statuses are defined in broad terms, encompassing a variety of living arrangements. The PIR measures family income relative to poverty thresholds, which are adjusted according to family size.

### Statistical analysis

This study conducted a descriptive statistical analysis of baseline characteristics linked to TyG-related markers. For continuous variables, the Kruskal–Wallis test was used, whereas Fisher's exact test was applied for categorical variables with low expected counts. The concentrations of heavy metals were normalized through logarithmic transformation, and Pearson’s correlation assessed the interrelationships among nine distinct heavy metals.

Initial analysis involved both linear and nonlinear regression techniques to evaluate the influence of heavy metals on TyG markers.

Additionally, the study employed Weighted Quantile Sum (WQS) regression to investigate both combined and individual effects of heavy metals on TyG markers, creating a weighted index for each metal and utilizing significant indices for in-depth analysis. The dataset was divided, allocating 40% for training and 60% for validation.

Bayesian Kernel Machine Regression (BKMR) was used to perform an extensive analysis of heavy metal mixtures across different exposure quartiles. This approach determined the relevance of each metal through posterior inclusion probability (PIP) set at a threshold of 0.5, examining both single and combined metal effects at various percentiles.

A mediation analysis employing nonparametric bootstrapping (*n* = 1000) was conducted to ascertain both direct and indirect effects of inflammation.

Demographic factors were adjusted for, and analyses including WQS, BKMR, and mediation were carried out using R software, targeting a significance threshold of *P* < 0.05 [37, 38].

## Results

### General information

Table 1 displays the demographic characteristics of 2,050 participants from the NHANES 2011–2016 data, categorized by TyG index quartiles. Significant variations were noted across the groups in variables including gender, age, race, education, marital status, weight, BMI, waist measurements, and there is a significant upward trend in the TyG index among males, Non-Hispanic Whites, high school graduates/GED or equivalent, married, and divorced individuals (*P* < 0.01). In contrast, females, Non-Hispanic Blacks, and those with a college degree or higher show a significant negative correlation (*P* < 0.01). Other groups generally exhibit a positive correlation. Moreover, levels of Cu, Zn, Pb, along with all inflammation markers except for Baso (all *P* < 0.05). Similar patterns of correlation were also seen across other indices (referenced in Table S1 to S3). Figure S1 indicates a generally weak correlation among most metals.

**Table 1:** Characteristics of participants (Based on TyG quartiles)

	Quartile 1	Quartile 2	Quartile 3	Quartile 4	P-value
**Populaion**	513	513	512	512	
**Gender**					
Male	204 (39.77%)	248 (48.34%)	277 (54.10%)	299 (58.40%)	**0.0000**
Female	309 (60.23%)	265 (51.66%)	235 (45.90%)	213 (41.60%)	
**Age**	42.04 ± 16.85	50.04 ± 18.25	50.27 ± 16.93	52.95 ± 15.45	**0.0000**
**Race**					
Mexican American	50 (9.75%)	67 (13.06%)	78 (15.23%)	79 (15.43%)	**0.0000**
Other Hispanic	43 (8.38%)	56 (10.92%)	71 (13.87%)	67 (13.09%)	
Non-Hispanic White	190 (37.04%)	195 (38.01%)	209 (40.82%)	233 (45.51%)	
Non-Hispanic Black	153 (29.82%)	111 (21.64%)	76 (14.84%)	58 (11.33%)	
Other Race—Including Multi-Racial	77 (15.01%)	84 (16.37%)	78 (15.23%)	75 (14.65%)	
**Educational Level**					
Less than 9th grade	22 (4.29%)	51 (9.94%)	53 (10.35%)	61 (11.91%)	**0.0000**
9-11th grade (Includes 12th grade with no diploma)	44 (8.58%)	65 (12.67%)	64 (12.50%)	73 (14.26%)	
High school graduate/GED or equivalent	104 (20.27%)	104 (20.27%)	122 (23.83%)	122 (23.83%)	
Some college or AA degree	166 (32.36%)	148 (28.85%)	139 (27.15%)	153 (29.88%)	
College graduate or above	177 (34.50%)	145 (28.27%)	134 (26.17%)	103 (20.12%)	
**Marital Status**					
Married	242 (47.17%)	265 (51.66%)	272 (53.12%)	283 (55.27%)	**0.0000**
Widowed	13 (2.53%)	47 (9.16%)	32 (6.25%)	37 (7.23%)	
Divorced	46 (8.97%)	51 (9.94%)	56 (10.94%)	70 (13.67%)	
Separated	13 (2.53%)	13 (2.53%)	16 (3.12%)	20 (3.91%)	
Never married	145 (28.27%)	102 (19.88%)	88 (17.19%)	64 (12.50%)	
Living with partner	54 (10.53%)	35 (6.82%)	48 (9.38%)	38 (7.42%)	
**PIR**	2.61 ± 1.67	2.48 ± 1.65	2.41 ± 1.65	2.33 ± 1.50	0.0328
**Body Measure**					
Weight	74.59 ± 19.84	79.69 ± 21.78	84.64 ± 22.18	88.21 ± 21.48	**0.0000**
Height	166.94 ± 9.55	167.27 ± 9.70	167.41 ± 10.47	167.86 ± 10.31	0.5203
BMI	26.73 ± 6.71	28.36 ± 6.89	30.11 ± 7.04	31.20 ± 6.60	**0.0000**
Waist	91.36 ± 15.82	97.92 ± 16.02	102.48 ± 15.80	106.39 ± 14.76	**0.0000**
**Heavy Metal**					
Cu	118.53 ± 30.39	121.43 ± 29.55	121.57 ± 33.41	117.15 ± 28.37	**0.0476**
Zn	85.12 ± 13.98	87.54 ± 14.32	88.27 ± 14.91	89.94 ± 15.11	**0.0000**
IHg	0.27 ± 0.23	0.27 ± 0.20	0.27 ± 0.28	0.28 ± 0.25	0.9194
EtHg	0.11 ± 0.03	0.11 ± 0.01	0.11 ± 0.03	0.11 ± 0.03	0.3313
MeHg	1.47 ± 2.46	1.53 ± 2.65	1.22 ± 1.92	1.32 ± 2.54	0.1701
Pb	1.26 ± 1.32	1.54 ± 2.09	1.33 ± 1.01	1.43 ± 1.42	**0.0208**
Cd	0.46 ± 0.47	0.53 ± 0.65	0.48 ± 0.53	0.55 ± 0.64	0.0528
Hg	1.64 ± 2.51	1.71 ± 2.73	1.41 ± 1.97	1.53 ± 2.62	0.2322
Mn	10.19 ± 3.68	10.46 ± 4.64	10.15 ± 3.48	9.90 ± 3.55	0.1466
**Inflammation Factor**					
WBC	6.20 ± 1.90	6.58 ± 1.77	7.04 ± 2.14	7.41 ± 2.12	**0.0000**
Lym	31.91 ± 8.66	30.49 ± 8.75	30.64 ± 8.80	30.60 ± 8.77	**0.0296**
Mono	8.36 ± 2.41	8.06 ± 2.13	7.84 ± 2.15	7.64 ± 1.91	**0.0000**
Neu	56.27 ± 9.58	57.66 ± 9.41	57.66 ± 9.73	58.10 ± 9.67	**0.0142**
Eos	2.76 ± 2.12	3.09 ± 2.09	3.17 ± 2.40	2.98 ± 1.90	**0.0124**
Baso	0.77 ± 0.47	0.78 ± 0.51	0.75 ± 0.44	0.74 ± 0.34	0.5032

### Association between single heavy metal and TyG-related Indicators

**Fig. 2:**
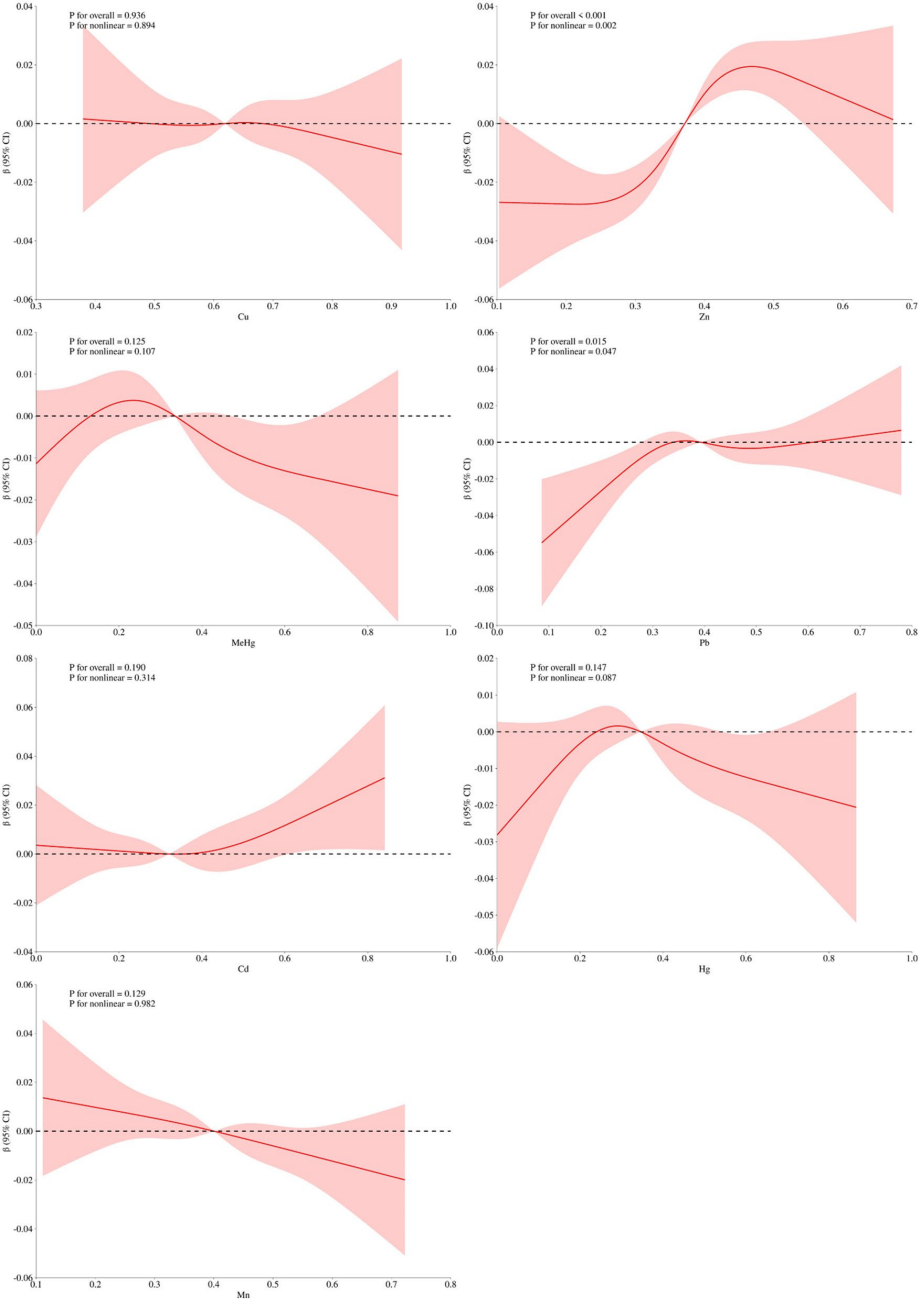
Weighted restricted cubic spline curve describing the non-linear association between heavy metal exposure and TyG index (Unadjusted)

In Fig. 2 and Figures S2 to S4, the analysis using weighted restricted cubic spline curves indicates a nonlinear connection between heavy metal exposure and TyG-related indices. Specifically, an inverted Z-shaped pattern is noted between copper (Cu) and the indices of TyGWC, TyGWHtR, and TyGBMI. Zinc (Zn) also shows an inverted Z-shaped pattern with the TyG index.

Mercury (MeHg: Organic mercury, primarily through exposure from consumption of contaminated fish and seafood), lead (Pb), and manganese (Mn) demonstrate an inverted U-shaped relationship with the TyGWC, TyGWHtR, and TyGBMI indices. Lead (Pb) in particular maintains this inverted U-shaped pattern consistently across various assessments with the TyG index.

Cadmium (Cd) exhibits a U-shaped curve with the TyGWC and TyGBMI indices, while mercury (Hg: Total mercury includes all forms of mercury through inhalation, ingestion and occupational exposure) presents an inverted U-shaped pattern with these indices. The TyGWC index is notably negatively associated with these metals.

Upon demographic adjustments (Figures S5 to S8), manganese's (Mn) association with the TyGWC index disappears. Further adjustments for demographic and immunization factors (Figures S9 to S12) alter the relationships of lead (Pb) with the TyG index, and both lead (Pb) and cadmium (Cd) with the TyGWC index, and lead (Pb) with the TyGWHtR index, into a Z-shaped pattern.

### Linear Association between Mixed heavy Metal and TyG-related Indicators

The relationship between mixed heavy metal exposure and TyG-related markers was analyzed using multiple linear regression. Initial unadjusted models revealed linear positive correlations of copper (Cu) with TyGWC, TyGWHtR, TyGBMI, and zinc (Zn) with TyG index. Conversely, linear negative correlations were observed for lead (Pb) with TyGBMI, cadmium (Cd) with TyGWC and TyGBMI, and manganese (Mn) with TyG, TyGWC, and TyGBMI (referenced in Table S3). After adjusting for various covariates, most of these associations persisted across the four TyG-related markers (referenced in Table S4).

### Weights and Effect Direction of Mixed Heavy Metals on TyG-related Indicators

The WQS model was utilized to explore the combined influence of nine heavy metals on TyG-related markers. According to Table S5, the WQS index showed a positive correlation between these metal mixtures and the TyG indicators, with the following estimates: TyG: 0.0316 (95% CI: 0.0158, 0.0473); TyGWC: 0.0470 (95% CI: 0.0244, 0.0695); TyGWHtR: 0.0699 (95% CI: 0.0510, 0.0888); TyGBMI: 0.0465 (95% CI: 0.0298, 0.0632). Zinc (Zn) was found to contribute the most to the TyG index with a weight of 0.4485, while copper (Cu) held the highest weights in the other indices: TyGWC (0.3773), TyGWHtR (0.5217), and TyGBMI (0.5333). Upon adjusting for all covariates, it was notable that Zn maintained the highest weight across the TyG, TyGWC, TyGWHtR, and TyGBMI indices (Tables S6 to S9).

### Non-linear Association between Mixed Heavy Metals and TyG-related Indicators

**Fig. 3:**
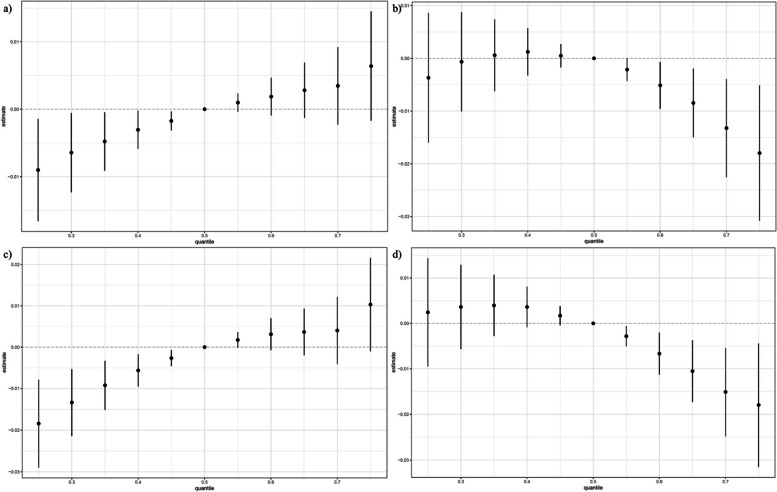
Overall effect of heavy metals mixtures on the TyG-related indicators in BKMR model where all heavy metals at specific percentiles were compared to their 50th percentile. (Unadjusted). *Note: a) TyG; b) TyGWC; c) TyGWHtR; d) TyGBMI*

**Fig. 4:**
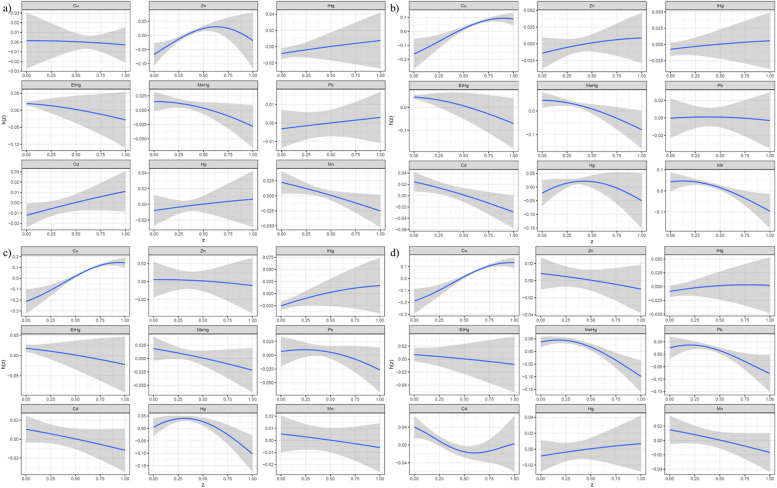
Univariate exposure–response function between each heavy metal and the TyG-related indicators when the other heavy metals were fixed at 50th percentiles. (Unadjusted). *Note: a) TyG; b) TyGWC; c) TyGWHtR; d) TyGBMI*

**Fig. 5:**
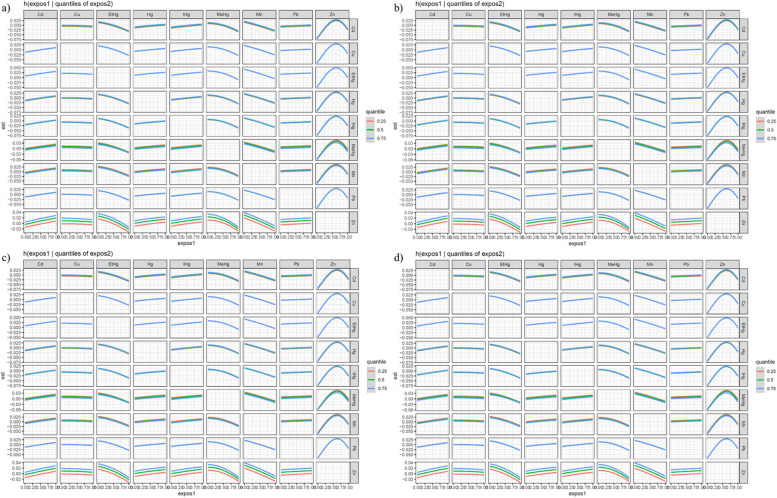
Single exposure–response functions for each heavy metal and the TyG-related indicators when a single heavy metal was at the 75th compared with the 50th percentile and the concentrations of all the other heavy metals were fixed at either the 25th, 50th, 75th percentile in the BKMR model. (Unadjusted). *Note: a) TyG; b) TyGWC; c) TyGWHtR; d) TyGBMI*

In the initial BKMR analysis, posterior inclusion probabilities (PIPs) were high for most heavy metals regarding TyG-related markers. Unadjusted models showed positive correlations with TyG and TyGWHtR, and inverted U-shaped correlations with TyGWC and TyGBMI (Fig. 3). Figure 4 illustrates varied relationships between individual metals and TyG markers when other metals are at median levels. Furthermore, Fig. 5 indicates that manganese (Mn) consistently negatively impacted TyG markers, whereas copper (Cu) generally had a positive influence, except for the TyG index.

Adjustments for covariates altered the relationships for TyGWC and TyGBMI from inverted U-shaped to positive (Figure S13 to S16). While the nature of most relationships remained stable after these adjustments, the confidence intervals broadened (Figure S17 to S20). Figures S21 to S24 confirmed that trends in heavy metal impacts on TyG markers did not significantly change with control for the 25th, 50th, and 75th percentiles of other metals. No significant interactions were observed between the metals across these percentiles (Figures S25 to S28). Post-adjustment, high PIP values for each metal persisted (Table S10).

### Mediating role of inflammation factors in the association between heavy metals and TyG-related Indicators

Table S11 indicates no correlation between Baso and any metals, a finding that persisted even after adjusting for covariates. Furthermore, Table S12 confirms a significant link between WBC and Eos across all four indices, which remained consistent after covariate adjustments.

According to Table S13, under an unadjusted model, six inflammatory factors mediated the impacts of multiple heavy metals (Pb, Cd, Hg, Cu, EtHg, MeHg) on TyG-related indicators, including TyG, TyGWC, and TyGWHtR. When adjusted merely for demographic factors (age, gender, race, education, marital status, PIR), these inflammatory mediators continued to affect these pathways, though some pathways weakened (Table S14). However, after comprehensive covariate adjustments, these inflammatory mediators no longer influenced the relationship between Cu and TyG (Table S15).

### Additional analysis

Multiple linear regression analysis was employed to analysis the association between heavy metal mixture and TG and BG. In unadjusted and adjusted models, Zn was identified as the independent predictors of TG and in the unadjusted model, Mn was identified as the independent predictors of BG. (Table 2).

**Table 2:** Association between Heavy Metal Exposure and BG and TG

Variables	Single Factor					Multiple Factor				
	β	S.E	t	*P*	β (95%CI)	β	S.E	t	*P*	β (95%CI)
**TG (Model I)**										
Cu	-45.2087	23.4845	-1.925	0.0544	-45.2087 (-91.2375 ~ 0.8201)	-44.1131	24.0096	-1.8373	0.0663	-44.1131 (-91.1710 ~ 2.9448)
Zn	97.0097	21.7857	4.4529	** < .0001**	97.0097 (54.3104 ~ 139.7090)	97.2952	21.8614	4.4505	** < .0001**	97.2952 (54.4477 ~ 140.1428)
IHg	16.5284	17.2874	0.9561	0.3391	16.5284 (-17.3543 ~ 50.4111)	23.0424	21.7787	1.058	0.2902	23.0424 (-19.6430 ~ 65.7279)
EtHg	-14.2832	34.6751	-0.4119	0.6804	-14.2832 (-82.2452 ~ 53.6788)	-17.2707	34.8061	-0.4962	0.6198	-17.2707 (-85.4895 ~ 50.9481)
MeHg	-2.49	10.694	-0.2328	0.8159	-2.4900 (-23.4499 ~ 18.4698)	-3.4766	36.3226	-0.0957	0.9238	-3.4766 (-74.6676 ~ 67.7144)
Pb	-1.4351	18.9073	-0.0759	0.9395	-1.4351 (-38.4927 ~ 35.6226)	-20.5084	20.4556	-1.0026	0.3162	-20.5084 (-60.6007 ~ 19.5839)
Cd	9.454	12.4479	0.7595	0.4477	9.4540 (-14.9435 ~ 33.8515)	19.5705	13.3854	1.4621	0.1439	19.5705 (-6.6644 ~ 45.8055)
Hg	-0.87	13.0529	-0.0667	0.9469	-0.8700 (-26.4532 ~ 24.7132)	-5.8442	47.5625	-0.1229	0.9022	-5.8442 (-99.0650 ~ 87.3766)
Mn	-36.8725	19.4288	-1.8978	0.0579	-36.8725 (-74.9522 ~ 1.2073)	-37.6719	19.7075	-1.9115	0.0561	-37.6719 (-76.2979 ~ 0.9541)
**BG (Model I)**										
Cu	10.1379	8.2679	1.2262	0.2203	10.1379 (-6.0669 ~ 26.3426)	14.0545	8.4672	1.6599	0.0971	14.0545 (-2.5410 ~ 30.6500)
Zn	7.9623	7.7007	1.034	0.3013	7.9623 (-7.1308 ~ 23.0554)	9.134	7.7097	1.1847	0.2363	9.1340 (-5.9767 ~ 24.2446)
IHg	-0.6992	6.0842	-0.1149	0.9085	-0.6992 (-12.6240 ~ 11.2257)	-3.1692	7.6805	-0.4126	0.6799	-3.1692 (-18.2227 ~ 11.8843)
EtHg	-14.8679	12.1971	-1.219	0.223	-14.8679 (-38.7739 ~ 9.0380)	-13.8387	12.2748	-1.1274	0.2597	-13.8387 (-37.8968 ~ 10.2195)
MeHg	-4.4713	3.7616	-1.1887	0.2347	-4.4713 (-11.8439 ~ 2.9014)	-14.2496	12.8096	-1.1124	0.2661	-14.2496 (-39.3559 ~ 10.8567)
Pb	7.5791	6.6508	1.1396	0.2546	7.5791 (-5.4562 ~ 20.6144)	8.5048	7.2139	1.1789	0.2386	8.5048 (-5.6342 ~ 22.6437)
Cd	-0.92	4.3806	-0.21	0.8337	-0.9200 (-9.5058 ~ 7.6658)	-2.3214	4.7205	-0.4918	0.6229	-2.3214 (-11.5734 ~ 6.9307)
Hg	-4.2687	4.5919	-0.9296	0.3527	-4.2687 (-13.2687 ~ 4.7313)	15.2809	16.7735	0.911	0.3624	15.2809 (-17.5944 ~ 48.1563)
Mn	-24.6377	6.8207	-3.6122	**0.0003**	-24.6377 (-38.0060 ~ -11.2695)	-24.6951	6.9501	-3.5532	**0.0004**	-24.6951 (-38.3170 ~ -11.0732)
**TG (Model II)**										
Cu	-45.2087	23.4845	-1.925	0.0544	-45.2087 (-91.2375 ~ 0.8201)	47.0986	26.8676	1.753	0.0798	47.0986 (-5.5609 ~ 99.7581)
Zn	97.0097	21.7857	4.4529	** < .0001**	97.0097 (54.3104 ~ 139.7090)	69.5027	21.8086	3.1869	**0.0015**	69.5027 (26.7587 ~ 112.2467)
IHg	16.5284	17.2874	0.9561	0.3391	16.5284 (-17.3543 ~ 50.4111)	19.7921	21.4952	0.9208	0.3573	19.7921 (-22.3377 ~ 61.9220)
EtHg	-14.2832	34.6751	-0.4119	0.6804	-14.2832 (-82.2452 ~ 53.6788)	-10.9429	34.2313	-0.3197	0.7492	-10.9429 (-78.0351 ~ 56.1493)
MeHg	-2.49	10.694	-0.2328	0.8159	-2.4900 (-23.4499 ~ 18.4698)	6.1652	35.9218	0.1716	0.8637	6.1652 (-64.2402 ~ 76.5706)
Pb	-1.4351	18.9073	-0.0759	0.9395	-1.4351 (-38.4927 ~ 35.6226)	-85.2146	22.6581	-3.7609	**0.0002**	-85.2146 (-129.6236 ~ -40.8055)
Cd	9.454	12.4479	0.7595	0.4477	9.4540 (-14.9435 ~ 33.8515)	26.342	13.9587	1.8871	0.0593	26.3420 (-1.0165 ~ 53.7005)
Hg	-0.87	13.0529	-0.0667	0.9469	-0.8700 (-26.4532 ~ 24.7132)	-1.4412	47.37	-0.0304	0.9757	-1.4412 (-94.2847 ~ 91.4023)
Mn	-36.8725	19.4288	-1.8978	0.0579	-36.8725 (-74.9522 ~ 1.2073)	-38.2789	20.8697	-1.8342	0.0668	-38.2789 (-79.1828 ~ 2.6249)
**BG (Model II)**										
Cu	10.1379	8.2679	1.2262	0.2203	10.1379 (-6.0669 ~ 26.3426)	24.3845	9.3392	2.611	**0.0091**	24.3845 (6.0801 ~ 42.6890)
Zn	7.9623	7.7007	1.034	0.3013	7.9623 (-7.1308 ~ 23.0554)	8.9202	7.5807	1.1767	0.2395	8.9202 (-5.9376 ~ 23.7781)
IHg	-0.6992	6.0842	-0.1149	0.9085	-0.6992 (-12.6240 ~ 11.2257)	-6.3306	7.4718	-0.8473	0.3969	-6.3306 (-20.9749 ~ 8.3138)
EtHg	-14.8679	12.1971	-1.219	0.223	-14.8679 (-38.7739 ~ 9.0380)	-10.2666	11.8988	-0.8628	0.3883	-10.2666 (-33.5879 ~ 13.0547)
MeHg	-4.4713	3.7616	-1.1887	0.2347	-4.4713 (-11.8439 ~ 2.9014)	-11.7876	12.4864	-0.944	0.3453	-11.7876 (-36.2605 ~ 12.6854)
Pb	7.5791	6.6508	1.1396	0.2546	7.5791 (-5.4562 ~ 20.6144)	-32.8982	7.876	-4.177	** < .0001**	-32.8982 (-48.3348 ~ -17.4616)
Cd	-0.92	4.3806	-0.21	0.8337	-0.9200 (-9.5058 ~ 7.6658)	-2.1161	4.852	-0.4361	0.6628	-2.1161 (-11.6260 ~ 7.3937)
Hg	-4.2687	4.5919	-0.9296	0.3527	-4.2687 (-13.2687 ~ 4.7313)	15.7486	16.4659	0.9564	0.339	15.7486 (-16.5239 ~ 48.0211)
Mn	-24.6377	6.8207	-3.6122	**0.0003**	-24.6377 (-38.0060 ~ -11.2695)	-13.0949	7.2543	-1.8051	0.0712	-13.0949 (-27.3131 ~ 1.1233)

**Fig. 6:**
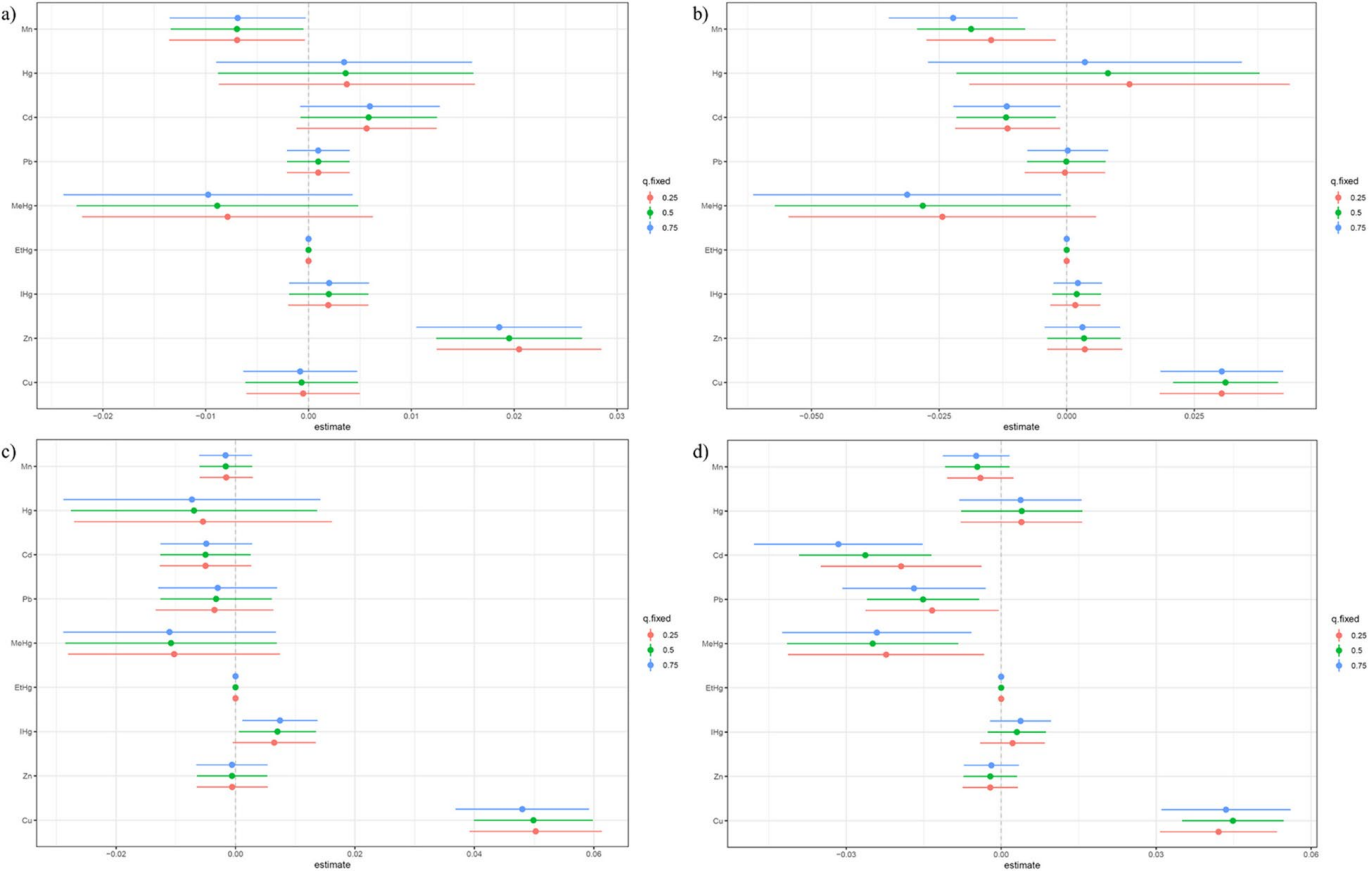
Bivariate exposure–response functions for each heavy metal and the TyG-related indicators when one heavy metal was fixed at 25th, 50th, 75th percentiles and other heavy metals were fixed at the median in the BKMR model. (Unadjusted). *Note: a) TyG; b) TyGWC; c) TyGWHtR; d) TyGBMI*

Employing BKMR model, it was found that heavy metal exposure has a positive effect on TG and a negative effect on BG. Different heavy metals showed different associations with TG and BG. No interaction effects were observed. (Figure S29 ~ Figure S30) Fig. 6.

## Discussion

Cardiovascular ailments are among the leading causes of death and disability, severely affecting life expectancy and overall well-being. The exposure to heavy metals in the bloodstream is identified as a contributing factor to these conditions [11]. Studies have indicated that lead exacerbates the risk of cardiovascular diseases [39], whereas elements like zinc and selenium are believed to have protective effects [40, 41]. Additionally, the TyG index and associated measures are recognized as robust indicators for predicting cardiovascular mortality [23], underscoring the importance of studying how heavy metals interact with the TyG index.

Recent research has predominantly examined the impacts of specific heavy metals such as lead (Pb) and cadmium (Cd) on TyG metrics, revealing significant correlations in subjects participating in HOMA-IR studies [10]. Despite these findings, there remains a notable lack of comprehensive analysis on the effects of combined heavy metal exposures on TyG indices, suggesting an area ripe for further investigation [10].

The current research has established that a combination of heavy metals can elevate the risks associated with TyG and related markers, indicating potential cumulative or synergistic effects that warrant deeper exploration.

Research increasingly highlights that inflammation and oxidative stress play critical roles in the onset and progression of cardiovascular diseases through various mechanisms. TNF-α and IL-6 notably disrupt endothelial function [42], promoting the migration of monocytes and macrophages to arterial lesions [43]. These cells eventually transform into lipid-laden foamy cells, initiating plaque formation [44]. Additionally, under the influence of PDGF, vascular smooth muscle cells proliferate and migrate, forming a fibrous cap that significantly heightens the risk of plaque instability and potentially leading to severe cardiovascular incidents like myocardial infarction [45, 46]. Chronic inflammation is also implicated in causing structural changes in blood vessels, exacerbating the progression of atherosclerosis [47].

Metals have been identified as catalysts for both inflammatory responses and oxidative stress through several pathways [12]. They might indirectly initiate inflammation by producing free radicals that cause oxidative stress and cellular damage [22], or directly by prompting apoptosis and necrosis via the release of DAMPs [48]. Additionally, heavy metals are capable of activating the NF-κB pathway, which enhances the production of pro-inflammatory cytokines such as IL-1β, IL-6, and TNF-α [49].

This research aimed to determine whether blood levels of heavy metals could increase the TyG index through pathways of inflammation and oxidative stress. It was found that white blood cells (WBC) mainly mediated the positive associations found between heavy metal concentrations and rising TyG index levels, with both monocytes (Mono) and lymphocytes (Lym) also showing mediation effects for various metals and indices. This suggests that exposure to heavy metals may increase the TyG index by fostering inflammatory responses.

In multi-group sensitivity analyses, adjustments for various covariates revealed that lead (Pb) might be inversely associated with the TyG index, contrasting with its slight positive association in models without adjustments. This inverse relationship might stem from lead's interference with glucose metabolism and insulin signaling pathways, which could prompt insulin resistance, and its effects on lipid metabolism, potentially impacting liver functions and serum triglyceride concentrations [50, 51]. Future studies should delve into the metabolic pathways of lead and its prolonged effects on the TyG index.

Furthermore, after accounting for demographic factors, the association between manganese (Mn) and the TyG index became non-significant, suggesting that variables like age, socioeconomic status, and cultural factors could influence Mn's metabolic impact due to their effects on dietary choices and physical activity [52].

Post-adjustment analyses showed that no inflammatory markers mediated the relationship between copper (Cu) and the TyG index. It appears that controlling for covariates associated with Cu, inflammatory markers, and TyG indices negated the role of inflammation in this connection [53]. The adjustments for demographic and physical characteristics likely emphasized these variables' primary influence over the TyG index, reducing the apparent impact of inflammatory processes [54].

### Study strength and limitations

This research offers multiple advantages. It pioneers the investigation into how inflammatory markers might influence the link between mixed heavy metal exposures and the TyG index, applying various statistical techniques and adjusting for numerous confounders to enhance the study's credibility and thoroughness. The data were sourced from the extensive NHANES database, known for its stringent quality assurance processes, which contribute to the trustworthiness of the findings.

Despite these strengths, the study faces certain limitations. Its cross-sectional nature limits the ability to establish causative links between exposure to heavy metals and changes in the TyG index. Furthermore, the research does not account for the effects of chronic exposure to heavy metals nor does it consider individual exposures to other environmental pollutants or lifestyle factors, which could influence the precision of the results.

## Conclusion

Generalized linear regression, WQS regression, and BKMR regression models were utilized to assess the association between mixtures of nine heavy metals and the TyG, along with its related indices, using inflammatory factors as mediating variables. By synthesizing the results from these three models, it can be concluded that there is a positive correlation between heavy metal mixtures and the TyG, with Zn having the strongest effect. Among the individual components, all metals except manganese showed a positive correlation. In subsequent mediation analysis, WBC demonstrated the strongest mediating effect among all inflammatory factors. The study provides a detailed analysis of the significant impact of mixed exposure to several common heavy metal components on the TyG. It also reveals the importance of considering whether patients are affected by internal inflammation when using the TyG in clinical applications.

## Supplementary Information

Supplementary Material 1.

Supplementary Material 2.

## Data Availability

Data available on request.
