# Supplementary material for: Association between Heavy metals and triglyceride-glucose-related index: a mediation analysis of inflammation indicators
Source: Lipids Health Dis. 2025 Feb 13;24:46. doi: 10.1186/s12944-025-02441-9 (PMC11823045; doi:10.1186/s12944-025-02441-9)
Supplement: Supplementary file 2 — Supplementary Material 2. [file 12944_2025_2441_MOESM2_ESM.docx]

Table S1 Characteristics of participants (Based on TyGWC quartiles)

|  | Quartile 1 | Quartile 2 | Quartile 3 | Quartile 4 | P-value |
| --- | --- | --- | --- | --- | --- |
| **Populaion** | 513 | 513 | 512 | 512 |  |
| **Gender** |  |  |  |  |  |
| Male | 197 (38.40%) | 259 (50.49%) | 290 (56.64%) | 282 (55.08%) | **0.0000** |
| Female | 316 (61.60%) | 254 (49.51%) | 222 (43.36%) | 230 (44.92%) |  |
| **Age** | 40.56 ± 16.74 | 49.90 ± 17.77 | 52.10 ± 16.47 | 52.73 ± 15.69 | **0.0000** |
| **Race** |  |  |  |  |  |
| Mexican American | 48 (9.36%) | 68 (13.26%) | 77 (15.04%) | 81 (15.82%) | **0.0000** |
| Other Hispanic | 53 (10.33%) | 62 (12.09%) | 65 (12.70%) | 57 (11.13%) |  |
| Non-Hispanic White | 190 (37.04%) | 197 (38.40%) | 202 (39.45%) | 238 (46.48%) |  |
| Non-Hispanic Black | 92 (17.93%) | 100 (19.49%) | 95 (18.55%) | 111 (21.68%) |  |
| Other Race - Including Multi-Racial | 130 (25.34%) | 86 (16.76%) | 73 (14.26%) | 25 (4.88%) |  |
| **Educational Level** |  |  |  |  |  |
| Less than 9th grade | 29 (5.65%) | 48 (9.36%) | 51 (9.96%) | 59 (11.52%) | **0.0000** |
| 9-11th grade (Includes 12th grade with no diploma) | 55 (10.72%) | 60 (11.70%) | 64 (12.50%) | 67 (13.09%) |  |
| High school graduate/GED or equivalent | 92 (17.93%) | 114 (22.22%) | 120 (23.44%) | 126 (24.61%) |  |
| Some college or AA degree | 145 (28.27%) | 140 (27.29%) | 163 (31.84%) | 158 (30.86%) |  |
| College graduate or above | 192 (37.43%) | 151 (29.43%) | 114 (22.27%) | 102 (19.92%) |  |
| **Marital Status** |  |  |  |  |  |
| Married | 234 (45.61%) | 269 (52.44%) | 292 (57.03%) | 267 (52.15%) | **0.0000** |
| Widowed | 16 (3.12%) | 40 (7.80%) | 32 (6.25%) | 41 (8.01%) |  |
| Divorced | 40 (7.80%) | 52 (10.14%) | 67 (13.09%) | 64 (12.50%) |  |
| Separated | 15 (2.92%) | 13 (2.53%) | 21 (4.10%) | 13 (2.54%) |  |
| Never married | 159 (30.99%) | 91 (17.74%) | 60 (11.72%) | 89 (17.38%) |  |
| Living with partner | 49 (9.55%) | 48 (9.36%) | 40 (7.81%) | 38 (7.42%) |  |
| **PIR** | 2.59 ± 1.68 | 2.49 ± 1.66 | 2.53 ± 1.60 | 2.23 ± 1.52 | **0.0022** |
| **Body Measure** |  |  |  |  |  |
| Weight | 62.02 ± 10.21 | 74.08 ± 11.16 | 84.67 ± 12.88 | 106.39 ± 21.61 | **0.0000** |
| Height | 165.48 ± 9.09 | 166.43 ± 9.87 | 168.28 ± 10.28 | 169.30 ± 10.33 | **0.0000** |
| BMI | 22.61 ± 3.04 | 26.74 ± 3.30 | 29.92 ± 3.94 | 37.14 ± 6.95 | **0.0000** |
| Waist | 80.94 ± 6.95 | 93.67 ± 5.80 | 103.47 ± 6.42 | 120.11 ± 12.52 | **0.0000** |
| **Heavy Metal** |  |  |  |  |  |
| Cu | 115.45 ± 30.58 | 119.25 ± 30.64 | 119.76 ± 28.74 | 124.23 ± 31.53 | **0.0001** |
| Zn | 87.47 ± 15.45 | 87.25 ± 13.57 | 88.63 ± 16.33 | 87.52 ± 13.13 | 0.4328 |
| IHg | 0.27 ± 0.27 | 0.27 ± 0.25 | 0.27 ± 0.19 | 0.26 ± 0.24 | 0.9261 |
| EtHg | 0.11 ± 0.03 | 0.11 ± 0.04 | 0.11 ± 0.02 | 0.11 ± 0.01 | 0.1193 |
| MeHg | 1.88 ± 3.01 | 1.34 ± 1.94 | 1.41 ± 2.95 | 0.91 ± 1.13 | **0.0000** |
| Pb | 1.43 ± 1.99 | 1.47 ± 1.57 | 1.36 ± 0.99 | 1.29 ± 1.33 | 0.2379 |
| Cd | 0.53 ± 0.58 | 0.50 ± 0.51 | 0.50 ± 0.64 | 0.49 ± 0.57 | 0.7533 |
| Hg | 2.04 ± 3.06 | 1.52 ± 1.97 | 1.62 ± 3.09 | 1.11 ± 1.13 | **0.0000** |
| Mn | 10.67 ± 4.23 | 10.39 ± 3.71 | 9.85 ± 3.98 | 9.79 ± 3.46 | **0.0004** |
| **Inflammation Factor** |  |  |  |  |  |
| WBC | 6.16 ± 1.84 | 6.59 ± 1.85 | 6.99 ± 2.05 | 7.49 ± 2.16 | **0.0000** |
| Lym | 32.33 ± 8.46 | 31.08 ± 8.62 | 30.34 ± 9.34 | 29.89 ± 8.41 | **0.0000** |
| Mono | 8.13 ± 2.21 | 8.07 ± 2.18 | 7.99 ± 2.18 | 7.71 ± 2.10 | **0.0114** |
| Neu | 55.92 ± 9.38 | 57.03 ± 9.44 | 58.02 ± 10.11 | 58.71 ± 9.31 | **0.0000** |
| Eos | 2.92 ± 2.56 | 3.11 ± 2.19 | 2.99 ± 1.83 | 2.99 ± 1.91 | 0.5880 |
| Baso | 0.76 ± 0.48 | 0.77 ± 0.50 | 0.74 ± 0.40 | 0.77 ± 0.38 | 0.7329 |

Table S2 Characteristics of participants (Based on TyGWHtR quartiles)

|  | Quartile 1 | Quartile 2 | Quartile 3 | Quartile 4 | P-value |
| --- | --- | --- | --- | --- | --- |
| **Populaion** | 513 | 513 | 512 | 512 |  |
| **Gender** |  |  |  |  |  |
| Male | 252 (49.12%) | 284 (55.36%) | 280 (54.69%) | 212 (41.41%) | **0.0000** |
| Female | 261 (50.88%) | 229 (44.64%) | 232 (45.31%) | 300 (58.59%) |  |
| **Age** | 40.64 ± 16.71 | 48.83 ± 17.44 | 52.77 ± 16.70 | 53.06 ± 15.69 | **0.0000** |
| **Race** |  |  |  |  |  |
| Mexican American | 39 (7.60%) | 66 (12.87%) | 76 (14.84%) | 93 (18.16%) | **0.0000** |
| Other Hispanic | 52 (10.14%) | 53 (10.33%) | 68 (13.28%) | 64 (12.50%) |  |
| Non-Hispanic White | 202 (39.38%) | 206 (40.16%) | 201 (39.26%) | 218 (42.58%) |  |
| Non-Hispanic Black | 98 (19.10%) | 104 (20.27%) | 92 (17.97%) | 104 (20.31%) |  |
| Other Race - Including Multi-Racial | 122 (23.78%) | 84 (16.37%) | 75 (14.65%) | 33 (6.45%) |  |
| **Educational Level** |  |  |  |  |  |
| Less than 9th grade | 23 (4.48%) | 43 (8.38%) | 48 (9.38%) | 73 (14.26%) | **0.0000** |
| 9-11th grade (Includes 12th grade with no diploma) | 55 (10.72%) | 57 (11.11%) | 67 (13.09%) | 67 (13.09%) |  |
| High school graduate/GED or equivalent | 96 (18.71%) | 105 (20.47%) | 136 (26.56%) | 115 (22.46%) |  |
| Some college or AA degree | 139 (27.10%) | 157 (30.60%) | 147 (28.71%) | 163 (31.84%) |  |
| College graduate or above | 200 (38.99%) | 151 (29.43%) | 114 (22.27%) | 94 (18.36%) |  |
| **Marital Status** |  |  |  |  |  |
| Married | 239 (46.59%) | 283 (55.17%) | 283 (55.27%) | 257 (50.20%) | **0.0000** |
| Widowed | 10 (1.95%) | 31 (6.04%) | 35 (6.84%) | 53 (10.35%) |  |
| Divorced | 39 (7.60%) | 50 (9.75%) | 67 (13.09%) | 67 (13.09%) |  |
| Separated | 12 (2.34%) | 16 (3.12%) | 18 (3.52%) | 16 (3.12%) |  |
| Never married | 166 (32.36%) | 82 (15.98%) | 67 (13.09%) | 84 (16.41%) |  |
| Living with partner | 47 (9.16%) | 51 (9.94%) | 42 (8.20%) | 35 (6.84%) |  |
| **PIR** | 2.59 ± 1.66 | 2.66 ± 1.69 | 2.47 ± 1.58 | 2.11 ± 1.50 | **0.0000** |
| **Body Measure** |  |  |  |  |  |
| Weight | 64.44 ± 12.21 | 76.22 ± 13.99 | 83.84 ± 15.13 | 102.66 ± 24.10 | **0.0000** |
| Height | 168.34 ± 9.49 | 168.47 ± 9.97 | 167.19 ± 9.68 | 165.48 ± 10.62 | **0.0000** |
| BMI | 22.61 ± 3.00 | 26.68 ± 3.16 | 29.86 ± 3.85 | 37.26 ± 6.95 | **0.0000** |
| Waist | 81.83 ± 7.94 | 94.20 ± 7.45 | 103.12 ± 8.16 | 119.03 ± 13.38 | **0.0000** |
| **Heavy Metal** |  |  |  |  |  |
| Cu | 112.30 ± 29.20 | 118.58 ± 30.84 | 119.13 ± 28.76 | 128.70 ± 31.05 | **0.0000** |
| Zn | 87.57 ± 14.92 | 88.21 ± 14.66 | 88.03 ± 15.24 | 87.05 ± 13.87 | 0.5886 |
| IHg | 0.26 ± 0.17 | 0.27 ± 0.31 | 0.28 ± 0.21 | 0.27 ± 0.25 | 0.6946 |
| EtHg | 0.12 ± 0.04 | 0.11 ± 0.03 | 0.11 ± 0.02 | 0.11 ± 0.01 | 0.0547 |
| MeHg | 1.84 ± 2.95 | 1.42 ± 2.26 | 1.39 ± 2.77 | 0.90 ± 1.17 | **0.0000** |
| Pb | 1.44 ± 2.02 | 1.42 ± 1.10 | 1.45 ± 1.52 | 1.25 ± 1.25 | 0.1001 |
| Cd | 0.52 ± 0.58 | 0.50 ± 0.52 | 0.47 ± 0.53 | 0.53 ± 0.67 | 0.4000 |
| Hg | 2.00 ± 3.02 | 1.59 ± 2.22 | 1.60 ± 2.95 | 1.09 ± 1.18 | **0.0000** |
| Mn | 10.35 ± 4.15 | 10.28 ± 3.64 | 10.04 ± 4.04 | 10.04 ± 3.62 | 0.4563 |
| **Inflammation Factor** |  |  |  |  |  |
| WBC | 6.17 ± 1.86 | 6.52 ± 1.82 | 6.95 ± 2.02 | 7.59 ± 2.16 | **0.0000** |
| Lym | 32.35 ± 8.42 | 30.88 ± 8.85 | 30.34 ± 9.08 | 30.07 ± 8.51 | **0.0001** |
| Mono | 8.20 ± 2.26 | 8.18 ± 2.19 | 7.92 ± 2.12 | 7.60 ± 2.06 | **0.0000** |
| Neu | 55.77 ± 9.41 | 57.13 ± 9.67 | 58.20 ± 9.73 | 58.58 ± 9.43 | **0.0000** |
| Eos | 2.97 ± 2.62 | 3.11 ± 2.05 | 2.89 ± 1.88 | 3.05 ± 1.93 | 0.3828 |
| Baso | 0.78 ± 0.51 | 0.77 ± 0.49 | 0.72 ± 0.38 | 0.78 ± 0.38 | 0.0659 |

Table S3 Characteristics of participants (Based on TyGBMI quartiles)

|  | Quartile 1 | Quartile 2 | Quartile 3 | Quartile 4 | P-value |
| --- | --- | --- | --- | --- | --- |
| **Populaion** | 513 | 513 | 512 | 512 |  |
| **Gender** |  |  |  |  |  |
| Male | 230 (44.83%) | 278 (54.19%) | 285 (55.66%) | 235 (45.90%) | **0.0003** |
| Female | 283 (55.17%) | 235 (45.81%) | 227 (44.34%) | 277 (54.10%) |  |
| **Age** | 43.39 ± 18.23 | 51.33 ± 17.92 | 50.76 ± 16.83 | 49.81 ± 15.21 | **0.0000** |
| **Race** |  |  |  |  |  |
| Mexican American | 47 (9.16%) | 59 (11.50%) | 82 (16.02%) | 86 (16.80%) | **0.0000** |
| Other Hispanic | 48 (9.36%) | 59 (11.50%) | 71 (13.87%) | 59 (11.52%) |  |
| Non-Hispanic White | 207 (40.35%) | 215 (41.91%) | 196 (38.28%) | 209 (40.82%) |  |
| Non-Hispanic Black | 74 (14.42%) | 96 (18.71%) | 96 (18.75%) | 132 (25.78%) |  |
| Other Race - Including Multi-Racial | 137 (26.71%) | 84 (16.37%) | 67 (13.09%) | 26 (5.08%) |  |
| **Educational Level** |  |  |  |  |  |
| Less than 9th grade | 37 (7.21%) | 44 (8.58%) | 50 (9.77%) | 56 (10.94%) | **0.0000** |
| 9-11th grade (Includes 12th grade with no diploma) | 56 (10.92%) | 60 (11.70%) | 65 (12.70%) | 65 (12.70%) |  |
| High school graduate/GED or equivalent | 97 (18.91%) | 100 (19.49%) | 132 (25.78%) | 123 (24.02%) |  |
| Some college or AA degree | 139 (27.10%) | 146 (28.46%) | 154 (30.08%) | 167 (32.62%) |  |
| College graduate or above | 184 (35.87%) | 163 (31.77%) | 111 (21.68%) | 101 (19.73%) |  |
| **Marital Status** |  |  |  |  |  |
| Married | 242 (47.17%) | 284 (55.36%) | 283 (55.27%) | 253 (49.41%) | **0.0000** |
| Widowed | 24 (4.68%) | 35 (6.82%) | 33 (6.45%) | 37 (7.23%) |  |
| Divorced | 43 (8.38%) | 52 (10.14%) | 62 (12.11%) | 66 (12.89%) |  |
| Separated | 13 (2.53%) | 17 (3.31%) | 17 (3.32%) | 15 (2.93%) |  |
| Never married | 149 (29.04%) | 79 (15.40%) | 72 (14.06%) | 99 (19.34%) |  |
| Living with partner | 42 (8.19%) | 46 (8.97%) | 45 (8.79%) | 42 (8.20%) |  |
| **PIR** | 2.52 ± 1.67 | 2.61 ± 1.65 | 2.47 ± 1.58 | 2.24 ± 1.56 | **0.0020** |
| **Body Measure** |  |  |  |  |  |
| Weight | 61.14 ± 9.33 | 74.25 ± 10.75 | 84.29 ± 11.15 | 107.49 ± 21.17 | **0.0000** |
| Height | 166.75 ± 9.21 | 167.78 ± 10.31 | 167.56 ± 9.88 | 167.39 ± 10.59 | 0.3902 |
| BMI | 21.91 ± 2.21 | 26.27 ± 1.91 | 29.96 ± 2.39 | 38.27 ± 6.18 | **0.0000** |
| Waist | 81.53 ± 7.62 | 94.23 ± 7.03 | 102.75 ± 7.48 | 119.68 ± 12.91 | **0.0000** |
| **Heavy Metal** |  |  |  |  |  |
| Cu | 114.18 ± 30.24 | 118.57 ± 30.47 | 117.75 ± 27.80 | 128.20 ± 31.77 | **0.0000** |
| Zn | 87.91 ± 15.50 | 87.55 ± 14.04 | 88.76 ± 15.91 | 86.64 ± 13.05 | 0.1401 |
| IHg | 0.27 ± 0.27 | 0.28 ± 0.27 | 0.27 ± 0.17 | 0.26 ± 0.24 | 0.7407 |
| EtHg | 0.12 ± 0.04 | 0.11 ± 0.02 | 0.11 ± 0.02 | 0.11 ± 0.01 | **0.0196** |
| MeHg | 1.90 ± 3.11 | 1.44 ± 2.32 | 1.29 ± 2.52 | 0.92 ± 1.20 | **0.0000** |
| Pb | 1.51 ± 2.04 | 1.53 ± 1.53 | 1.37 ± 1.10 | 1.15 ± 1.17 | **0.0001** |
| Cd | 0.55 ± 0.61 | 0.51 ± 0.49 | 0.45 ± 0.56 | 0.50 ± 0.64 | **0.0460** |
| Hg | 2.07 ± 3.17 | 1.62 ± 2.31 | 1.49 ± 2.70 | 1.11 ± 1.21 | **0.0000** |
| Mn | 10.49 ± 4.13 | 10.37 ± 3.74 | 9.86 ± 3.99 | 9.98 ± 3.56 | **0.0254** |
| **Inflammation Factor** |  |  |  |  |  |
| WBC | 6.30 ± 1.94 | 6.49 ± 1.84 | 6.97 ± 1.97 | 7.47 ± 2.18 | **0.0000** |
| Lym | 31.99 ± 8.77 | 30.21 ± 8.76 | 31.00 ± 9.01 | 30.44 ± 8.41 | **0.0056** |
| Mono | 8.14 ± 2.15 | 8.18 ± 2.27 | 7.93 ± 2.10 | 7.66 ± 2.13 | **0.0004** |
| Neu | 56.29 ± 9.57 | 57.73 ± 9.75 | 57.40 ± 9.68 | 58.26 ± 9.39 | **0.0090** |
| Eos | 2.89 ± 2.49 | 3.18 ± 2.20 | 3.01 ± 1.92 | 2.94 ± 1.89 | 0.1493 |
| Baso | 0.76 ± 0.49 | 0.78 ± 0.50 | 0.74 ± 0.38 | 0.77 ± 0.40 | 0.6742 |

Table S3 Association between heavy metal exposure and TyG-related indicators (Unadjusted)

| Independent Variable | TyG | TyGWC | TyGWHtR | TyGBMI |
| --- | --- | --- | --- | --- |
| Cu | -0.004 (-0.061, 0.053)  p = 0.8829 | 0.241 (0.163, 0.319)  p = 0.0000 | 0.382 (0.306, 0.459)  p = 0.0000 | 0.317 (0.250, 0.384)  p = 0.0000 |
| Zn | 0.142 (0.090, 0.193)  p = 0.0000 | 0.042 (-0.029, 0.113)  p = 0.2517 | -0.008 (-0.077, 0.062)  p = 0.8257 | -0.027 (-0.089, 0.034)  p = 0.3842 |
| IHg | 0.019 (-0.033, 0.071)  p = 0.4736 | 0.033 (-0.037, 0.104)  p = 0.3552 | 0.042 (-0.027, 0.112)  p = 0.2304 | 0.010 (-0.051, 0.071)  p = 0.7459 |
| EtHg | -0.037 (-0.119, 0.046)  p = 0.3848 | -0.089 (-0.202, 0.024)  p = 0.1223 | -0.093 (-0.204, 0.018)  p = 0.1002 | -0.082 (-0.180, 0.016)  p = 0.1004 |
| MeHg | -0.050 (-0.136, 0.036)  p = 0.2524 | -0.062 (-0.180, 0.056)  p = 0.3051 | -0.106 (-0.221, 0.010)  p = 0.0737 | -0.065 (-0.167, 0.037)  p = 0.2100 |
| Pb | 0.032 (-0.017, 0.080)  p = 0.1998 | 0.007 (-0.059, 0.074)  p = 0.8313 | -0.018 (-0.083, 0.048)  p = 0.5963 | -0.107 (-0.165, -0.050)  p = 0.0003 |
| Cd | 0.022 (-0.010, 0.053)  p = 0.1817 | -0.065 (-0.108, -0.021)  p = 0.0036 | -0.037 (-0.080, 0.006)  p = 0.0902 | -0.071 (-0.108, -0.033)  p = 0.0002 |
| Hg | 0.037 (-0.076, 0.150)  p = 0.5182 | -0.032 (-0.186, 0.123)  p = 0.6871 | 0.039 (-0.112, 0.191)  p = 0.6108 | 0.015 (-0.119, 0.148)  p = 0.8309 |
| Mn | -0.055 (-0.101, -0.008)  p = 0.0218 | -0.134 (-0.198, -0.070)  p = 0.0000 | -0.062 (-0.125, 0.001)  p = 0.0522 | -0.074 (-0.129, -0.019)  p = 0.0087 |

Table S4 Association between mixed heavy metal exposure and TyG-related indicators (Adjusted)

|  | TyG | | |  | TyGWC | | |
| --- | --- | --- | --- | --- | --- | --- | --- |
|  | Model I | Model II | Model III |  | Model I | Model II | Model III |
| Cu | 0.069 (0.008, 0.129)  p = 0.0258 | -0.023 (-0.082, 0.036)  p = 0.4496 | -0.037 (-0.095, 0.021)  p = 0.2123 |  | 0.385 (0.304, 0.465)  p = 0.0000 | -0.022 (-0.055, 0.011)  p = 0.1917 | -0.030 (-0.062, 0.003)  p = 0.0752 |
| Zn | 0.121 (0.071, 0.172)  p = 0.0000 | 0.136 (0.088, 0.184)  p = 0.0000 | 0.127 (0.080, 0.174)  p = 0.0000 |  | 0.004 (-0.063, 0.071)  p = 0.8998 | 0.075 (0.048, 0.102)  p = 0.0000 | 0.069 (0.043, 0.096)  p = 0.0000 |
| IHg | 0.008 (-0.042, 0.058)  p = 0.7516 | 0.003 (-0.044, 0.050)  p = 0.9052 | 0.012 (-0.034, 0.058)  p = 0.6095 |  | 0.017 (-0.049, 0.083)  p = 0.6155 | -0.000 (-0.027, 0.026)  p = 0.9738 | 0.004 (-0.021, 0.030)  p = 0.7388 |
| EtHg | -0.019 (-0.098, 0.060)  p = 0.6421 | -0.012 (-0.087, 0.063)  p = 0.7582 | 0.001 (-0.072, 0.074)  p = 0.9778 |  | -0.056 (-0.162, 0.049)  p = 0.2941 | -0.007 (-0.050, 0.035)  p = 0.7357 | -0.000 (-0.041, 0.041)  p = 0.9932 |
| MeHg | -0.052 (-0.134, 0.031)  p = 0.2190 | -0.034 (-0.113, 0.044)  p = 0.3896 | -0.038 (-0.114, 0.038)  p = 0.3228 |  | -0.066 (-0.176, 0.045)  p = 0.2431 | -0.021 (-0.065, 0.024)  p = 0.3604 | -0.023 (-0.065, 0.020)  p = 0.2995 |
| Pb | -0.125 (-0.178, -0.073)  p = 0.0000 | -0.076 (-0.126, -0.026)  p = 0.0031 | -0.070 (-0.119, -0.021)  p = 0.0049 |  | -0.270 (-0.340, -0.200)  p = 0.0000 | -0.043 (-0.072, -0.015)  p = 0.0028 | -0.040 (-0.067, -0.012)  p = 0.0047 |
| Cd | 0.032 (0.000, 0.064)  p = 0.0481 | 0.045 (0.014, 0.075)  p = 0.0042 | 0.020 (-0.010, 0.050)  p = 0.1859 |  | -0.040 (-0.083, 0.002)  p = 0.0646 | 0.025 (0.008, 0.042)  p = 0.0047 | 0.011 (-0.006, 0.028)  p = 0.1890 |
| Hg | 0.065 (-0.044, 0.173)  p = 0.2447 | 0.063 (-0.041, 0.166)  p = 0.2347 | 0.063 (-0.037, 0.163)  p = 0.2165 |  | 0.010 (-0.135, 0.155)  p = 0.8934 | 0.037 (-0.022, 0.095)  p = 0.2177 | 0.037 (-0.020, 0.093)  p = 0.2022 |
| Mn | 0.003 (-0.042, 0.049)  p = 0.8819 | -0.009 (-0.053, 0.036)  p = 0.7030 | -0.013 (-0.056, 0.030)  p = 0.5552 |  | -0.027 (-0.088, 0.035)  p = 0.3963 | -0.011 (-0.036, 0.014)  p = 0.3935 | -0.013 (-0.037, 0.011)  p = 0.2824 |
|  | TyGWHtR | | |  | TyGBMI | | |
|  | Model I | Model II | Model III |  | Model I | Model II | Model III |
| Cu | 0.368 (0.289, 0.447)  p = 0.0000 | -0.025 (-0.058, 0.008)  p = 0.1435 | -0.031 (-0.064, 0.001)  p = 0.0579 |  | 0.365 (0.292, 0.438)  p = 0.0000 | -0.016 (-0.039, 0.007)  p = 0.1627 | -0.021 (-0.043, 0.002)  p = 0.0688 |
| Zn | -0.005 (-0.070, 0.061)  p = 0.8880 | 0.077 (0.050, 0.104)  p = 0.0000 | 0.072 (0.046, 0.098)  p = 0.0000 |  | -0.041 (-0.102, 0.019)  p = 0.1817 | 0.051 (0.032, 0.069)  p = 0.0000 | 0.047 (0.029, 0.065)  p = 0.0000 |
| IHg | 0.010 (-0.055, 0.075)  p = 0.7722 | 0.001 (-0.026, 0.027)  p = 0.9544 | 0.006 (-0.020, 0.031)  p = 0.6700 |  | -0.004 (-0.064, 0.056)  p = 0.9069 | -0.001 (-0.019, 0.017)  p = 0.9078 | 0.002 (-0.015, 0.020)  p = 0.8124 |
| EtHg | -0.054 (-0.158, 0.049)  p = 0.3036 | -0.009 (-0.051, 0.033)  p = 0.6758 | -0.002 (-0.043, 0.039)  p = 0.9138 |  | -0.063 (-0.159, 0.032)  p = 0.1958 | -0.005 (-0.034, 0.024)  p = 0.7347 | -0.000 (-0.028, 0.027)  p = 0.9758 |
| MeHg | -0.091 (-0.199, 0.017)  p = 0.0986 | -0.021 (-0.065, 0.023)  p = 0.3540 | -0.023 (-0.065, 0.020)  p = 0.2972 |  | -0.067 (-0.166, 0.033)  p = 0.1905 | -0.012 (-0.042, 0.018)  p = 0.4322 | -0.013 (-0.042, 0.016)  p = 0.3688 |
| Pb | -0.252 (-0.320, -0.184)  p = 0.0000 | -0.044 (-0.072, -0.016)  p = 0.0023 | -0.041 (-0.068, -0.013)  p = 0.0037 |  | -0.240 (-0.303, -0.177)  p = 0.0000 | -0.030 (-0.049, -0.011)  p = 0.0023 | -0.028 (-0.046, -0.009)  p = 0.0038 |
| Cd | -0.048 (-0.090, -0.007)  p = 0.0227 | 0.023 (0.006, 0.040)  p = 0.0077 | 0.010 (-0.007, 0.027)  p = 0.2311 |  | -0.065 (-0.104, -0.027)  p = 0.0009 | 0.017 (0.005, 0.028)  p = 0.0055 | 0.008 (-0.004, 0.019)  p = 0.1863 |
| Hg | 0.061 (-0.081, 0.203)  p = 0.3996 | 0.037 (-0.021, 0.095)  p = 0.2065 | 0.037 (-0.019, 0.094)  p = 0.1942 |  | 0.046 (-0.085, 0.178)  p = 0.4900 | 0.023 (-0.017, 0.063)  p = 0.2555 | 0.023 (-0.015, 0.061)  p = 0.2419 |
| Mn | 0.008 (-0.052, 0.068)  p = 0.7847 | -0.012 (-0.036, 0.013)  p = 0.3554 | -0.014 (-0.038, 0.010)  p = 0.2520 |  | -0.025 (-0.080, 0.031)  p = 0.3792 | -0.008 (-0.024, 0.009)  p = 0.3788 | -0.009 (-0.026, 0.007)  p = 0.2667 |

*Note: Model I was adjusted for age, gender, race, educational level, marital status and PIR; Model II was adjusted for age, gender, race, educational level, marital status, PIR, weight, height, waist and BMI; Model III was adjusted for age, gender, race, educational level, marital status, PIR, weight, height, waist, BMI, WBC, Lym, Mono, Neu, Eos and Baso.*

Table S5 Association between heavy metal exposure and TyG-related indicators (Unadjusted)

|  | TyG | TyGWC | TyGWHtR | TyGBMI |
| --- | --- | --- | --- | --- |
| Estimate | 0.0316 (0.0158, 0.0473) | 0.0470 (0.0244, 0.0695) | 0.0699 (0.0510, 0.0888) | 0.0465 (0.0298, 0.0632) |
| P - value | 0.0001 | 0.0000 | 0.0000 | 0.0000 |
| Cu | 0.0065 | 0.3773 | 0.5217 | 0.5333 |
| Zn | 0.4485 | 0.2759 | 0.0833 | 0.1216 |
| IHg | 0.2406 | 0.1308 | 0.1901 | 0.0000 |
| EtHg | 0.1264 | 0.1751 | 0.1677 | 0.2464 |
| MeHg | 0.0458 | 0.0160 | 0.0090 | 0.0954 |
| Pb | 0.0333 | 0.0248 | 0.0202 | 0.0000 |
| Cd | 0.0707 | 0.0000 | 0.0000 | 0.0000 |
| Hg | 0.0116 | 0.0000 | 0.0007 | 0.0000 |
| Mn | 0.0167 | 0.0000 | 0.0073 | 0.0032 |

Table S6 Association between heavy metal exposure and TyG (Adjusted)

|  | Model I | Model II | Model III |
| --- | --- | --- | --- |
| Estimate | 0.0283 (0.0128, 0.0437) | 0.0300 (0.0161, 0.0439) | 0.0262 (0.0121, 0.0402) |
| P - value | 0.0004 | 0.0000 | 0.0003 |
| Cu | 0.0199 | 0.0014 | 0.0000 |
| Zn | 0.4257 | 0.4601 | 0.4355 |
| IHg | 0.2165 | 0.1684 | 0.1962 |
| EtHg | 0.1123 | 0.1307 | 0.1184 |
| MeHg | 0.0484 | 0.0632 | 0.0764 |
| Pb | 0.0000 | 0.0001 | 0.0000 |
| Cd | 0.0268 | 0.0528 | 0.0141 |
| Hg | 0.0119 | 0.0361 | 0.0438 |
| Mn | 0.1385 | 0.0872 | 0.1157 |

*Note: Model I was adjusted for age, gender, race, educational level, marital status and PIR; Model II was adjusted for age, gender, race, educational level, marital status, PIR, weight, height, waist and BMI; Model III was adjusted for age, gender, race, educational level, marital status, PIR, weight, height, waist, BMI, WBC, Lym, Mono, Neu, Eos and Baso.*

Table S7 Association between heavy metal exposure and TyGWC (Adjusted)

|  | Model I | Model II | Model III |
| --- | --- | --- | --- |
| Estimate | 0.2468 (0.1665, 0.3270) | 0.0167 (0.0091, 0.0242) | 0.0144 (0.0069, 0.0220) |
| P - value | 0.0000 | 0.0000 | 0.0002 |
| Cu | 0.1231 | 0.0014 | 0.0000 |
| Zn | 0.0573 | 0.4834 | 0.4686 |
| IHg | 0.0000 | 0.1669 | 0.1868 |
| EtHg | 0.8113 | 0.1124 | 0.1082 |
| MeHg | 0.0051 | 0.0827 | 0.0950 |
| Pb | 0.0000 | 0.0002 | 0.0000 |
| Cd | 0.0000 | 0.0601 | 0.0183 |
| Hg | 0.0008 | 0.0347 | 0.0448 |
| Mn | 0.0024 | 0.0582 | 0.0784 |

*Note: Model I was adjusted for age, gender, race, educational level, marital status and PIR; Model II was adjusted for age, gender, race, educational level, marital status, PIR, weight, height, waist and BMI; Model III was adjusted for age, gender, race, educational level, marital status, PIR, weight, height, waist, BMI, WBC, Lym, Mono, Neu, Eos and Baso.*

Table S8 Association between heavy metal exposure and TyGWHtR (Adjusted)

|  | Model I | Model II | Model III |
| --- | --- | --- | --- |
| Estimate | 0.0673 (0.0433, 0.0914) | 0.0174 (0.0098, 0.0250) | 0.0152 (0.0075, 0.0229) |
| P - value | 0.0000 | 0.0000 | 0.0001 |
| Cu | 0.4036 | 0.0013 | 0.0000 |
| Zn | 0.1703 | 0.4720 | 0.4524 |
| IHg | 0.1247 | 0.1776 | 0.1989 |
| EtHg | 0.2221 | 0.1124 | 0.1082 |
| MeHg | 0.0324 | 0.0799 | 0.0893 |
| Pb | 0.0000 | 0.0003 | 0.0000 |
| Cd | 0.0000 | 0.0518 | 0.0139 |
| Hg | 0.0000 | 0.0396 | 0.0505 |
| Mn | 0.0469 | 0.0651 | 0.0868 |

*Note: Model I was adjusted for age, gender, race, educational level, marital status and PIR; Model II was adjusted for age, gender, race, educational level, marital status, PIR, weight, height, waist and BMI; Model III was adjusted for age, gender, race, educational level, marital status, PIR, weight, height, waist, BMI, WBC, Lym, Mono, Neu, Eos and Baso.*

Table S9Association between heavy metal exposure and TyGBMI (Adjusted)

|  | Model I | Model II | Model III |
| --- | --- | --- | --- |
| Estimate | 0.0546 (0.0338, 0.0754) | 0.0116 (0.0065, 0.0166) | 0.0098 (0.0049, 0.0147) |
| P - value | 0.0000 | 0.0000 | 0.0001 |
| Cu | 0.4640 | 0.0012 | 0.0000 |
| Zn | 0.1093 | 0.4933 | 0.4959 |
| IHg | 0.0000 | 0.1536 | 0.1618 |
| EtHg | 0.2623 | 0.1029 | 0.0904 |
| MeHg | 0.1119 | 0.0850 | 0.1010 |
| Pb | 0.0000 | 0.0002 | 0.0000 |
| Cd | 0.0000 | 0.0605 | 0.0185 |
| Hg | 0.0000 | 0.0418 | 0.0542 |
| Mn | 0.0524 | 0.0615 | 0.0782 |

*Note: Model I was adjusted for age, gender, race, educational level, marital status and PIR; Model II was adjusted for age, gender, race, educational level, marital status, PIR, weight, height, waist and BMI; Model III was adjusted for age, gender, race, educational level, marital status, PIR, weight, height, waist, BMI, WBC, Lym, Mono, Neu, Eos and Baso.*

Table S10 PIPs of each heavy metal for TyG-related indicators in all BKMR model

|  | TyG | | | TyGWC | | | TyGWHtR | | | TyGBMI | | |
| --- | --- | --- | --- | --- | --- | --- | --- | --- | --- | --- | --- | --- |
|  | Model I | Model II | Model III | Model I | Model II | Model III | Model I | Model II | Model III | Model I | Model II | Model III |
| Cu | 0.60 | 0.48 | 0.42 | 1.00 | 0.22 | 1.00 | 1.00 | 0.82 | 1.00 | 1.00 | 0.22 | 1.00 |
| Zn | 1.00 | 1.00 | 1.00 | 0.26 | 1.00 | 1.00 | 0.68 | 1.00 | 1.00 | 1.00 | 1.00 | 1.00 |
| IHg | 0.78 | 0.16 | 1.00 | 0.00 | 0.20 | 0.78 | 0.58 | 0.60 | 1.00 | 0.04 | 0.20 | 0.78 |
| EtHg | 0.64 | 0.72 | 1.00 | 0.78 | 0.00 | 1.00 | 0.78 | 0.00 | 0.86 | 0.88 | 0.00 | 1.00 |
| MeHg | 0.00 | 1.00 | 0.58 | 1.00 | 0.00 | 0.00 | 1.00 | 1.00 | 0.92 | 0.98 | 0.00 | 0.00 |
| Pb | 1.00 | 0.44 | 1.00 | 1.00 | 0.82 | 1.00 | 1.00 | 1.00 | 1.00 | 1.00 | 0.82 | 1.00 |
| Cd | 1.00 | 0.44 | 0.96 | 0.46 | 0.50 | 0.72 | 1.00 | 0.62 | 0.96 | 1.00 | 0.50 | 0.72 |
| Hg | 0.64 | 0.64 | 0.84 | 0.96 | 0.00 | 1.00 | 0.50 | 0.24 | 0.90 | 0.56 | 0.00 | 1.00 |
| Mn | 0.60 | 0.30 | 1.00 | 1.00 | 0.14 | 0.80 | 0.66 | 0.22 | 1.00 | 0.76 | 0.14 | 0.80 |

*Note: Model I was adjusted for age, gender, race, educational level, marital status and PIR; Model II was adjusted for age, gender, race, educational level, marital status, PIR, weight, height, waist and BMI; Model III was adjusted for age, gender, race, educational level, marital status, PIR, weight, height, waist, BMI, WBC, Lym, Mono, Neu, Eos and Baso.*

Table S11 Association between heavy metal exposure and inflammation factors

|  | WBC | | |  | Lym | | |
| --- | --- | --- | --- | --- | --- | --- | --- |
|  | Model I | Model II | Model III |  | Model I | Model II | Model III |
| Cu | 0.236 (0.169, 0.302)  p = 0.0000 | 0.319 (0.246, 0.392)  p = 0.0000 | 0.234 (0.162, 0.307)  p = 0.0000 |  | -0.112 (-0.173, -0.051)  p = 0.0003 | -0.190 (-0.256, -0.125)  p = 0.0000 | -0.181 (-0.248, -0.113)  p = 0.0000 |
| Zn | -0.041 (-0.102, 0.019)  p = 0.1832 | -0.069 (-0.129, -0.008)  p = 0.0257 | -0.054 (-0.113, 0.005)  p = 0.0710 |  | 0.066 (0.010, 0.121)  p = 0.0204 | 0.086 (0.032, 0.141)  p = 0.0019 | 0.090 (0.036, 0.144)  p = 0.0012 |
| IHg | -0.033 (-0.093, 0.028)  p = 0.2898 | -0.035 (-0.095, 0.025)  p = 0.2484 | -0.041 (-0.099, 0.017)  p = 0.1626 |  | 0.001 (-0.055, 0.056)  p = 0.9775 | 0.010 (-0.044, 0.063)  p = 0.7274 | 0.015 (-0.039, 0.068)  p = 0.5915 |
| EtHg | -0.138 (-0.235, -0.042)  p = 0.0051 | -0.135 (-0.230, -0.040)  p = 0.0053 | -0.129 (-0.221, -0.037)  p = 0.0060 |  | 0.078 (-0.011, 0.166)  p = 0.0844 | 0.057 (-0.028, 0.143)  p = 0.1890 | 0.059 (-0.026, 0.145)  p = 0.1742 |
| MeHg | 0.013 (-0.088, 0.113)  p = 0.8040 | -0.002 (-0.102, 0.097)  p = 0.9626 | 0.017 (-0.079, 0.113)  p = 0.7316 |  | 0.022 (-0.070, 0.115)  p = 0.6344 | 0.016 (-0.073, 0.106)  p = 0.7196 | 0.017 (-0.072, 0.106)  p = 0.7119 |
| Pb | -0.081 (-0.137, -0.024)  p = 0.0053 | -0.100 (-0.163, -0.037)  p = 0.0019 | -0.062 (-0.123, -0.000)  p = 0.0499 |  | -0.106 (-0.158, -0.054)  p = 0.0001 | 0.034 (-0.023, 0.090)  p = 0.2451 | 0.030 (-0.027, 0.087)  p = 0.3028 |
| Cd | 0.132 (0.094, 0.169)  p = 0.0000 | 0.151 (0.113, 0.189)  p = 0.0000 | 0.164 (0.126, 0.201)  p = 0.0000 |  | -0.014 (-0.048, 0.020)  p = 0.4110 | -0.046 (-0.081, -0.012)  p = 0.0085 | -0.044 (-0.078, -0.009)  p = 0.0137 |
| Hg | -0.085 (-0.217, 0.046)  p = 0.2047 | -0.025 (-0.156, 0.105)  p = 0.7026 | -0.029 (-0.156, 0.098)  p = 0.6547 |  | 0.038 (-0.083, 0.158)  p = 0.5432 | 0.034 (-0.084, 0.152)  p = 0.5733 | 0.023 (-0.094, 0.141)  p = 0.6989 |
| Mn | -0.005 (-0.059, 0.050)  p = 0.8686 | 0.017 (-0.038, 0.073)  p = 0.5352 | 0.005 (-0.049, 0.059)  p = 0.8540 |  | -0.022 (-0.072, 0.028)  p = 0.3905 | -0.079 (-0.128, -0.029)  p = 0.0020 | -0.077 (-0.128, -0.027)  p = 0.0024 |
|  | Mono | | |  | Neu | | |
|  | Model I | Model II | Model III |  | Model I | Model II | Model III |
| Cu | -0.139 (-0.192, -0.086)  p = 0.0000 | -0.053 (-0.111, 0.005)  p = 0.0719 | -0.028 (-0.087, 0.032)  p = 0.3630 |  | 0.122 (0.066, 0.178)  p = 0.0000 | 0.152 (0.091, 0.213)  p = 0.0000 | 0.138 (0.076, 0.201)  p = 0.0000 |
| Zn | 0.009 (-0.040, 0.057)  p = 0.7243 | -0.009 (-0.057, 0.039)  p = 0.7095 | -0.012 (-0.060, 0.036)  p = 0.6288 |  | -0.023 (-0.074, 0.027)  p = 0.3669 | -0.036 (-0.086, 0.015)  p = 0.1658 | -0.038 (-0.089, 0.012)  p = 0.1356 |
| IHg | 0.004 (-0.044, 0.052)  p = 0.8796 | 0.009 (-0.039, 0.057)  p = 0.7090 | 0.012 (-0.035, 0.059)  p = 0.6212 |  | 0.027 (-0.024, 0.077)  p = 0.3011 | 0.015 (-0.035, 0.065)  p = 0.5656 | 0.010 (-0.040, 0.060)  p = 0.6989 |
| EtHg | -0.002 (-0.079, 0.075)  p = 0.9658 | 0.006 (-0.070, 0.082)  p = 0.8808 | 0.005 (-0.071, 0.080)  p = 0.9034 |  | -0.062 (-0.143, 0.019)  p = 0.1311 | -0.048 (-0.128, 0.031)  p = 0.2354 | -0.049 (-0.128, 0.030)  p = 0.2230 |
| MeHg | -0.003 (-0.084, 0.077)  p = 0.9353 | -0.007 (-0.086, 0.072)  p = 0.8683 | -0.011 (-0.090, 0.067)  p = 0.7761 |  | -0.017 (-0.101, 0.067)  p = 0.6949 | -0.009 (-0.092, 0.074)  p = 0.8240 | -0.009 (-0.092, 0.073)  p = 0.8247 |
| Pb | 0.120 (0.075, 0.165)  p = 0.0000 | 0.040 (-0.011, 0.090)  p = 0.1217 | 0.027 (-0.023, 0.078)  p = 0.2908 |  | 0.026 (-0.021, 0.073)  p = 0.2826 | -0.050 (-0.103, 0.003)  p = 0.0620 | -0.044 (-0.097, 0.009)  p = 0.1015 |
| Cd | -0.056 (-0.086, -0.027)  p = 0.0002 | -0.035 (-0.066, -0.004)  p = 0.0250 | -0.037 (-0.068, -0.007)  p = 0.0167 |  | 0.015 (-0.016, 0.046)  p = 0.3532 | 0.038 (0.006, 0.070)  p = 0.0212 | 0.036 (0.004, 0.068)  p = 0.0291 |
| Hg | 0.015 (-0.090, 0.120)  p = 0.7731 | 0.018 (-0.086, 0.122)  p = 0.7321 | 0.016 (-0.088, 0.120)  p = 0.7622 |  | -0.040 (-0.151, 0.070)  p = 0.4731 | -0.035 (-0.144, 0.074)  p = 0.5300 | -0.025 (-0.134, 0.084)  p = 0.6525 |
| Mn | -0.116 (-0.160, -0.073)  p = 0.0000 | -0.075 (-0.119, -0.031)  p = 0.0008 | -0.071 (-0.115, -0.027)  p = 0.0016 |  | 0.051 (0.005, 0.096)  p = 0.0296 | 0.078 (0.031, 0.124)  p = 0.0010 | 0.077 (0.030, 0.123)  p = 0.0012 |
|  | Eos | | |  | Baso | | |
|  | Model I | Model II | Model III |  | Model I | Model II | Model III |
| Cu | -0.078 (-0.121, -0.036)  p = 0.0003 | -0.050 (-0.096, -0.003)  p = 0.0383 | -0.064 (-0.112, -0.015)  p = 0.0096 |  | -0.005 (-0.062, 0.051)  p = 0.8494 | 0.003 (-0.059, 0.065)  p = 0.9240 | -0.004 (-0.068, 0.060)  p = 0.8932 |
| Zn | 0.040 (0.001, 0.078)  p = 0.0448 | 0.036 (-0.003, 0.075)  p = 0.0666 | 0.041 (0.002, 0.080)  p = 0.0391 |  | 0.027 (-0.025, 0.078)  p = 0.3098 | 0.022 (-0.030, 0.073)  p = 0.4120 | 0.025 (-0.027, 0.077)  p = 0.3418 |
| IHg | -0.054 (-0.093, -0.016)  p = 0.0058 | -0.051 (-0.089, -0.012)  p = 0.0097 | -0.049 (-0.088, -0.011)  p = 0.0120 |  | -0.028 (-0.080, 0.023)  p = 0.2744 | -0.033 (-0.085, 0.018)  p = 0.1991 | -0.032 (-0.083, 0.019)  p = 0.2165 |
| EtHg | 0.010 (-0.052, 0.071)  p = 0.7599 | 0.013 (-0.048, 0.074)  p = 0.6753 | 0.016 (-0.045, 0.077)  p = 0.6137 |  | -0.046 (-0.128, 0.035)  p = 0.2647 | -0.042 (-0.123, 0.040)  p = 0.3136 | -0.040 (-0.122, 0.041)  p = 0.3320 |
| MeHg | -0.040 (-0.104, 0.024)  p = 0.2198 | -0.043 (-0.107, 0.021)  p = 0.1852 | -0.041 (-0.105, 0.023)  p = 0.2137 |  | -0.068 (-0.153, 0.017)  p = 0.1177 | -0.060 (-0.145, 0.024)  p = 0.1625 | -0.058 (-0.143, 0.027)  p = 0.1836 |
| Pb | 0.086 (0.050, 0.122)  p = 0.0000 | 0.047 (0.007, 0.088)  p = 0.0219 | 0.057 (0.016, 0.098)  p = 0.0069 |  | 0.024 (-0.024, 0.072)  p = 0.3233 | -0.018 (-0.071, 0.036)  p = 0.5202 | -0.014 (-0.069, 0.040)  p = 0.6119 |
| Cd | -0.024 (-0.048, -0.000)  p = 0.0463 | -0.024 (-0.048, 0.001)  p = 0.0600 | -0.020 (-0.045, 0.005)  p = 0.1181 |  | 0.017 (-0.014, 0.048)  p = 0.2916 | 0.028 (-0.005, 0.061)  p = 0.0913 | 0.031 (-0.002, 0.064)  p = 0.0645 |
| Hg | 0.062 (-0.022, 0.146)  p = 0.1499 | 0.062 (-0.023, 0.146)  p = 0.1514 | 0.058 (-0.027, 0.142)  p = 0.1810 |  | 0.092 (-0.020, 0.203)  p = 0.1078 | 0.081 (-0.031, 0.192)  p = 0.1574 | 0.076 (-0.036, 0.188)  p = 0.1848 |
| Mn | 0.020 (-0.015, 0.055)  p = 0.2666 | 0.039 (0.003, 0.074)  p = 0.0332 | 0.039 (0.003, 0.074)  p = 0.0353 |  | -0.044 (-0.091, 0.002)  p = 0.0596 | -0.035 (-0.082, 0.013)  p = 0.1517 | -0.036 (-0.083, 0.012)  p = 0.1421 |

*Note: Model I was adjusted for age, gender, race, educational level, marital status and PIR; Model II was adjusted for age, gender, race, educational level, marital status, PIR, weight, height, waist and BMI; Model III was adjusted for age, gender, race, educational level, marital status, PIR, weight, height, waist, BMI, WBC, Lym, Mono, Neu, Eos and Baso.*

Table S12 Association between inflammation factors and TyG-related indicators

|  | TyG | | |  | TyGWC | | |
| --- | --- | --- | --- | --- | --- | --- | --- |
|  | Model I | Model II | Model III |  | Model I | Model II | Model III |
| WBC | 0.208 (0.169, 0.247)  p = 0.0000 | 0.217 (0.179, 0.255)  p = 0.0000 | 0.164 (0.126, 0.201)  p = 0.0000 |  | 0.320 (0.266, 0.374)  p = 0.0000 | 0.333 (0.281, 0.386)  p = 0.0000 | 0.089 (0.067, 0.110)  p = 0.0000 |
| Lym | 0.116 (0.015, 0.217)  p = 0.0246 | 0.213 (0.116, 0.310)  p = 0.0000 | 0.202 (0.108, 0.296)  p = 0.0000 |  | 0.070 (-0.069, 0.210)  p = 0.3219 | 0.196 (0.062, 0.330)  p = 0.0043 | 0.120 (0.067, 0.173)  p = 0.0000 |
| Mono | -0.052 (-0.104, 0.000)  p = 0.0515 | -0.128 (-0.179, -0.077)  p = 0.0000 | -0.120 (-0.169, -0.071)  p = 0.0000 |  | 0.010 (-0.062, 0.082)  p = 0.7934 | -0.090 (-0.161, -0.020)  p = 0.0115 | -0.069 (-0.096, -0.041)  p = 0.0000 |
| Neu | 0.089 (-0.030, 0.208)  p = 0.1430 | 0.097 (-0.017, 0.210)  p = 0.0946 | 0.085 (-0.024, 0.195)  p = 0.1272 |  | 0.128 (-0.036, 0.292)  p = 0.1261 | 0.125 (-0.032, 0.282)  p = 0.1178 | 0.056 (-0.006, 0.117)  p = 0.0771 |
| Eos | 0.155 (0.093, 0.217)  p = 0.0000 | 0.108 (0.049, 0.167)  p = 0.0003 | 0.090 (0.033, 0.147)  p = 0.0019 |  | 0.202 (0.117, 0.288)  p = 0.0000 | 0.142 (0.060, 0.223)  p = 0.0006 | 0.048 (0.016, 0.080)  p = 0.0035 |
| Baso | 0.020 (-0.023, 0.064)  p = 0.3641 | -0.005 (-0.046, 0.037)  p = 0.8200 | -0.010 (-0.050, 0.030)  p = 0.6344 |  | 0.061 (0.001, 0.121)  p = 0.0449 | 0.026 (-0.032, 0.083)  p = 0.3791 | -0.001 (-0.024, 0.021)  p = 0.9261 |
|  | TyGWHtR | | |  | TyGBMI | | |
|  | Model I | Model II | Model III |  | Model I | Model II | Model III |
| WBC | 0.324 (0.271, 0.378)  p = 0.0000 | 0.342 (0.291, 0.392)  p = 0.0000 | 0.085 (0.064, 0.106)  p = 0.0000 |  | 0.271 (0.224, 0.319)  p = 0.0000 | 0.272 (0.224, 0.320)  p = 0.0000 | 0.058 (0.043, 0.072)  p = 0.0000 |
| Lym | 0.109 (-0.029, 0.246)  p = 0.1216 | 0.198 (0.067, 0.329)  p = 0.0030 | 0.118 (0.065, 0.171)  p = 0.0000 |  | 0.157 (0.034, 0.280)  p = 0.0125 | 0.181 (0.058, 0.305)  p = 0.0040 | 0.082 (0.047, 0.118)  p = 0.0000 |
| Mono | -0.058 (-0.129, 0.013)  p = 0.1076 | -0.093 (-0.161, -0.025)  p = 0.0075 | -0.069 (-0.097, -0.041)  p = 0.0000 |  | -0.030 (-0.094, 0.034)  p = 0.3556 | -0.054 (-0.118, 0.011)  p = 0.1023 | -0.048 (-0.066, -0.029)  p = 0.0000 |
| Neu | 0.135 (-0.027, 0.297)  p = 0.1016 | 0.111 (-0.041, 0.264)  p = 0.1533 | 0.054 (-0.008, 0.115)  p = 0.0873 |  | 0.134 (-0.011, 0.279)  p = 0.0705 | 0.106 (-0.038, 0.250)  p = 0.1498 | 0.039 (-0.003, 0.081)  p = 0.0668 |
| Eos | 0.167 (0.083, 0.251)  p = 0.0001 | 0.144 (0.065, 0.223)  p = 0.0004 | 0.048 (0.016, 0.080)  p = 0.0032 |  | 0.143 (0.068, 0.218)  p = 0.0002 | 0.131 (0.057, 0.206)  p = 0.0006 | 0.033 (0.011, 0.055)  p = 0.0033 |
| Baso | 0.076 (0.016, 0.135)  p = 0.0124 | 0.030 (-0.026, 0.085)  p = 0.2978 | -0.000 (-0.023, 0.022)  p = 0.9997 |  | 0.048 (-0.005, 0.101)  p = 0.0743 | 0.029 (-0.024, 0.082)  p = 0.2830 | -0.000 (-0.016, 0.015)  p = 0.9783 |

*Note: Model I was adjusted for age, gender, race, educational level, marital status and PIR; Model II was adjusted for age, gender, race, educational level, marital status, PIR, weight, height, waist and BMI; Model III was adjusted for age, gender, race, educational level, marital status, PIR, weight, height, waist, BMI, WBC, Lym, Mono, Neu, Eos and Baso.*

Table S13 Mediating effect and proportions of inflammation factors between heavy metal exposure and TyG-related indexes (Unadjusted)

| Independent Variable | Intermediary Variable | Predictor Variable | Dierct Effects β (95% CI) | | | Indierct Effects β (95% CI) | | | Total Effects β (95% CI) | | | Mediated Proportion | P-value |
| --- | --- | --- | --- | --- | --- | --- | --- | --- | --- | --- | --- | --- | --- |
|  |  |  | Estimate | CI Lower | CI Upper | Estimate | CI Lower | CI Upper | Estimate | CI Lower | CI Upper |  |  |
| Pb | Eos | TyG | 0.0390 | -0.0044 | 0.0823 | 0.0072 | 0.0019 | 0.0126 | 0.0462 | 0.0030 | 0.0895 | 0.1568 | 0.0083 |
| Cd | WBC | TyG | -0.0007 | -0.0432 | 0.0418 | 0.0352 | 0.0233 | 0.0472 | 0.0345 | -0.0087 | 0.0778 | 1.0000 | 0.0000 |
| Cd | Mono | TyG | 0.0270 | -0.0161 | 0.0700 | 0.0076 | 0.0018 | 0.0133 | 0.0345 | -0.0087 | 0.0778 | 0.2193 | 0.0100 |
| Hg | WBC | TyG | 0.0124 | -0.0299 | 0.0548 | -0.0278 | -0.0392 | -0.0165 | -0.0154 | -0.0587 | 0.0279 | 1.0000 | 0.0000 |
| Hg | Neu | TyG | -0.0111 | -0.0544 | 0.0323 | -0.0044 | -0.0085 | -0.0002 | -0.0154 | -0.0587 | 0.0279 | 0.2832 | 0.0384 |
| Cu | Eos | TyG | -0.0023 | -0.0457 | 0.0411 | -0.0075 | -0.0129 | -0.0021 | -0.0098 | -0.0531 | 0.0335 | 0.7631 | 0.0067 |
| EtHg | WBC | TyG | -0.0017 | -0.0438 | 0.0405 | -0.0171 | -0.0278 | -0.0065 | -0.0188 | -0.0621 | 0.0245 | 0.9119 | 0.0016 |
| MeHg | WBC | TyG | 0.0003 | -0.0420 | 0.0426 | -0.0252 | -0.0364 | -0.0141 | -0.0249 | -0.0682 | 0.0183 | 1.0000 | 0.0000 |
| MeHg | Neu | TyG | -0.0204 | -0.0638 | 0.0229 | -0.0045 | -0.0088 | -0.0003 | -0.0249 | -0.0682 | 0.0183 | 0.1814 | 0.0371 |
| Pb | Mono | TyGBMI | -0.1297 | -0.1727 | -0.0867 | -0.0104 | -0.0167 | -0.0041 | -0.1401 | -0.1829 | -0.0972 | 0.0740 | 0.0013 |
| Hg | WBC | TyGBMI | -0.0812 | -0.1232 | -0.0391 | -0.0284 | -0.0399 | -0.0169 | -0.1096 | -0.1526 | -0.0666 | 0.2592 | 0.0000 |
| Hg | Neu | TyGBMI | -0.1048 | -0.1479 | -0.0617 | -0.0048 | -0.0091 | -0.0005 | -0.1096 | -0.1526 | -0.0666 | 0.0438 | 0.0275 |
| Cu | WBC | TyGBMI | 0.1642 | 0.1221 | 0.2062 | 0.0415 | 0.0292 | 0.0537 | 0.2056 | 0.1632 | 0.2480 | 0.2016 | 0.0000 |
| Cu | Mono | TyGBMI | 0.1945 | 0.1518 | 0.2372 | 0.0111 | 0.0040 | 0.0182 | 0.2056 | 0.1632 | 0.2480 | 0.0541 | 0.0021 |
| Cu | Neu | TyGBMI | 0.2002 | 0.1577 | 0.2428 | 0.0054 | 0.0002 | 0.0106 | 0.2056 | 0.1632 | 0.2480 | 0.0261 | 0.0422 |
| Zn | WBC | TyGBMI | -0.0269 | -0.0688 | 0.0150 | -0.0117 | -0.0228 | -0.0007 | -0.0387 | -0.0819 | 0.0046 | 0.3038 | 0.0369 |
| IHg | WBC | TyGBMI | 0.0069 | -0.0351 | 0.0489 | -0.0183 | -0.0296 | -0.0070 | -0.0114 | -0.0546 | 0.0319 | 1.0000 | 0.0016 |
| EtHg | WBC | TyGBMI | -0.0406 | -0.0826 | 0.0014 | -0.0181 | -0.0292 | -0.0069 | -0.0587 | -0.1019 | -0.0154 | 0.3079 | 0.0016 |
| MeHg | WBC | TyGBMI | -0.0919 | -0.1338 | -0.0499 | -0.0259 | -0.0372 | -0.0145 | -0.1177 | -0.1607 | -0.0747 | 0.2198 | 0.0000 |
| MeHg | Neu | TyGBMI | -0.1128 | -0.1558 | -0.0697 | -0.0050 | -0.0093 | -0.0006 | -0.1177 | -0.1607 | -0.0747 | 0.0421 | 0.0262 |
| Pb | Mono | TyGWC | -0.0277 | -0.0711 | 0.0157 | -0.0089 | -0.0149 | -0.0029 | -0.0366 | -0.0798 | 0.0067 | 0.2427 | 0.0039 |
| Hg | WBC | TyGWC | -0.0882 | -0.1299 | -0.0465 | -0.0310 | -0.0434 | -0.0186 | -0.1192 | -0.1622 | -0.0762 | 0.2600 | 0.0000 |
| Hg | Lym | TyGWC | -0.1104 | -0.1533 | -0.0676 | -0.0088 | -0.0145 | -0.0030 | -0.1192 | -0.1622 | -0.0762 | 0.0735 | 0.0031 |
| Hg | Neu | TyGWC | -0.1109 | -0.1538 | -0.0681 | -0.0082 | -0.0139 | -0.0026 | -0.1192 | -0.1622 | -0.0762 | 0.0692 | 0.0044 |
| Cu | WBC | TyGWC | 0.0804 | 0.0382 | 0.1226 | 0.0487 | 0.0351 | 0.0623 | 0.1291 | 0.0862 | 0.1720 | 0.3770 | 0.0000 |
| Cu | Lym | TyGWC | 0.1199 | 0.0770 | 0.1627 | 0.0092 | 0.0033 | 0.0151 | 0.1291 | 0.0862 | 0.1720 | 0.0713 | 0.0022 |
| Cu | Mono | TyGWC | 0.1200 | 0.0767 | 0.1633 | 0.0091 | 0.0022 | 0.0160 | 0.1291 | 0.0862 | 0.1720 | 0.0706 | 0.0097 |
| Cu | Neu | TyGWC | 0.1176 | 0.0747 | 0.1606 | 0.0115 | 0.0049 | 0.0180 | 0.1291 | 0.0862 | 0.1720 | 0.0888 | 0.0006 |
| IHg | WBC | TyGWC | 0.0107 | -0.0310 | 0.0525 | -0.0200 | -0.0323 | -0.0077 | -0.0092 | -0.0525 | 0.0341 | 1.0000 | 0.0015 |
| EtHg | WBC | TyGWC | -0.0341 | -0.0758 | 0.0076 | -0.0198 | -0.0319 | -0.0076 | -0.0539 | -0.0971 | -0.0107 | 0.3666 | 0.0014 |
| EtHg | Lym | TyGWC | -0.0485 | -0.0915 | -0.0055 | -0.0054 | -0.0107 | -0.0001 | -0.0539 | -0.0971 | -0.0107 | 0.1007 | 0.0456 |
| MeHg | WBC | TyGWC | -0.0966 | -0.1382 | -0.0550 | -0.0283 | -0.0405 | -0.0160 | -0.1249 | -0.1678 | -0.0819 | 0.2264 | 0.0000 |
| MeHg | Lym | TyGWC | -0.1162 | -0.1591 | -0.0734 | -0.0086 | -0.0144 | -0.0029 | -0.1249 | -0.1678 | -0.0819 | 0.0690 | 0.0033 |
| MeHg | Neu | TyGWC | -0.1163 | -0.1591 | -0.0734 | -0.0086 | -0.0143 | -0.0028 | -0.1249 | -0.1678 | -0.0819 | 0.0688 | 0.0034 |
| Pb | Mono | TyGWHtR | -0.0413 | -0.0845 | 0.0019 | -0.0142 | -0.0215 | -0.0069 | -0.0555 | -0.0988 | -0.0123 | 0.2560 | 0.0001 |
| Hg | WBC | TyGWHtR | -0.0682 | -0.1099 | -0.0266 | -0.0328 | -0.0458 | -0.0198 | -0.1010 | -0.1441 | -0.0579 | 0.3245 | 0.0000 |
| Hg | Lym | TyGWHtR | -0.0933 | -0.1363 | -0.0503 | -0.0077 | -0.0131 | -0.0023 | -0.1010 | -0.1441 | -0.0579 | 0.0763 | 0.0049 |
| Hg | Neu | TyGWHtR | -0.0926 | -0.1355 | -0.0497 | -0.0084 | -0.0142 | -0.0027 | -0.1010 | -0.1441 | -0.0579 | 0.0833 | 0.0042 |
| Cu | WBC | TyGWHtR | 0.1717 | 0.1302 | 0.2133 | 0.0479 | 0.0345 | 0.0613 | 0.2196 | 0.1774 | 0.2618 | 0.2181 | 0.0000 |
| Cu | Lym | TyGWHtR | 0.2124 | 0.1701 | 0.2546 | 0.0073 | 0.0021 | 0.0124 | 0.2196 | 0.1774 | 0.2618 | 0.0330 | 0.0057 |
| Cu | Mono | TyGWHtR | 0.2054 | 0.1629 | 0.2479 | 0.0142 | 0.0067 | 0.0217 | 0.2196 | 0.1774 | 0.2618 | 0.0649 | 0.0002 |
| Cu | Neu | TyGWHtR | 0.2092 | 0.1669 | 0.2515 | 0.0104 | 0.0042 | 0.0166 | 0.2196 | 0.1774 | 0.2618 | 0.0474 | 0.0010 |
| Zn | WBC | TyGWHtR | -0.0101 | -0.0516 | 0.0314 | -0.0135 | -0.0261 | -0.0009 | -0.0235 | -0.0668 | 0.0198 | 0.5722 | 0.0362 |
| Zn | Lym | TyGWHtR | -0.0178 | -0.0610 | 0.0253 | -0.0057 | -0.0107 | -0.0007 | -0.0235 | -0.0668 | 0.0198 | 0.2421 | 0.0252 |
| EtHg | WBC | TyGWHtR | -0.0362 | -0.0777 | 0.0054 | -0.0207 | -0.0334 | -0.0080 | -0.0569 | -0.1001 | -0.0136 | 0.3638 | 0.0014 |
| MeHg | WBC | TyGWHtR | -0.0866 | -0.1281 | -0.0450 | -0.0298 | -0.0426 | -0.0170 | -0.1163 | -0.1593 | -0.0733 | 0.2558 | 0.0000 |
| MeHg | Lym | TyGWHtR | -0.1088 | -0.1518 | -0.0659 | -0.0075 | -0.0128 | -0.0022 | -0.1163 | -0.1593 | -0.0733 | 0.0646 | 0.0055 |
| MeHg | Neu | TyGWHtR | -0.1076 | -0.1505 | -0.0647 | -0.0087 | -0.0145 | -0.0029 | -0.1163 | -0.1593 | -0.0733 | 0.0749 | 0.0033 |

Table S14 Mediating effect and proportions of inflammation factors between heavy metal exposure and TyG-related indexes

(Adjusted for age, gender, race, educational level, marital status and PIR)

| Independent Variable | Intermediary Variable | Predictor Variable | Dierct Effects β (95% CI) | | | Indierct Effects β (95% CI) | | | Total Effects β (95% CI) | | | Mediated Proportion | P-value |
| --- | --- | --- | --- | --- | --- | --- | --- | --- | --- | --- | --- | --- | --- |
|  |  |  | Estimate | CI Lower | CI Upper | Estimate | CI Lower | CI Upper | Estimate | CI Lower | CI Upper |  |  |
| Pb | Eos | TyG | -0.1091 | -0.1588 | -0.0593 | 0.0056 | 0.0006 | 0.0105 | -0.1035 | -0.1531 | -0.0539 | -0.0538 | 0.0285 |
| Cd | WBC | TyG | -0.0157 | -0.0455 | 0.0140 | 0.0243 | 0.0161 | 0.0324 | 0.0085 | -0.0218 | 0.0388 | 1.0000 | 0.0000 |
| Cd | Mono | TyG | 0.0051 | -0.0248 | 0.0351 | 0.0082 | 0.0025 | 0.0140 | 0.0134 | -0.0170 | 0.0437 | 0.6159 | 0.0052 |
| Hg | WBC | TyG | 0.0141 | -0.0161 | 0.0444 | -0.0198 | -0.0279 | -0.0118 | -0.0057 | -0.0366 | 0.0253 | 1.0000 | 0.0000 |
| Hg | Neu | TyG | 0.0025 | -0.0286 | 0.0336 | -0.0012 | -0.0035 | 0.0012 | 0.0014 | -0.0296 | 0.0324 | -0.8353 | **0.3429** |
| Cu | Eos | TyG | 0.0772 | 0.0165 | 0.1378 | -0.0066 | -0.0127 | -0.0006 | 0.0705 | 0.0101 | 0.1310 | -0.0941 | 0.0325 |
| EtHg | WBC | TyG | 0.0067 | -0.0703 | 0.0836 | -0.0324 | -0.0526 | -0.0123 | -0.0258 | -0.1049 | 0.0534 | 1.0000 | 0.0016 |
| MeHg | WBC | TyG | 0.0031 | -0.0216 | 0.0278 | -0.0147 | -0.0212 | -0.0083 | -0.0117 | -0.0369 | 0.0136 | 1.0000 | 0.0000 |
| MeHg | Neu | TyG | -0.0052 | -0.0306 | 0.0202 | -0.0010 | -0.0030 | 0.0011 | -0.0062 | -0.0315 | 0.0192 | 0.1547 | **0.3591** |
| Pb | Mono | TyGBMI | -0.2833 | -0.3444 | -0.2221 | -0.0163 | -0.0250 | -0.0077 | -0.2996 | -0.3607 | -0.2385 | 0.0545 | 0.0002 |
| Hg | WBC | TyGBMI | -0.0589 | -0.0967 | -0.0210 | -0.0242 | -0.0340 | -0.0143 | -0.0830 | -0.1217 | -0.0444 | 0.2912 | 0.0000 |
| Hg | Neu | TyGBMI | -0.0720 | -0.1108 | -0.0332 | -0.0025 | -0.0057 | 0.0007 | -0.0745 | -0.1132 | -0.0358 | 0.0339 | **0.1204** |
| Cu | WBC | TyGBMI | 0.2972 | 0.2237 | 0.3707 | 0.0611 | 0.0426 | 0.0796 | 0.3583 | 0.2845 | 0.4321 | 0.1706 | 0.0000 |
| Cu | Mono | TyGBMI | 0.3613 | 0.2870 | 0.4357 | 0.0245 | 0.0124 | 0.0365 | 0.3858 | 0.3118 | 0.4598 | 0.0634 | 0.0001 |
| Cu | Neu | TyGBMI | 0.3656 | 0.2911 | 0.4400 | 0.0033 | -0.0042 | 0.0107 | 0.3688 | 0.2947 | 0.4429 | 0.0089 | **0.3909** |
| Zn | WBC | TyGBMI | -0.0362 | -0.0976 | 0.0251 | -0.0165 | -0.0319 | -0.0010 | -0.0527 | -0.1158 | 0.0104 | 0.3124 | 0.0371 |
| IHg | WBC | TyGBMI | -0.0019 | -0.0503 | 0.0465 | -0.0202 | -0.0327 | -0.0076 | -0.0221 | -0.0718 | 0.0276 | 0.9135 | 0.0016 |
| EtHg | WBC | TyGBMI | -0.0731 | -0.1695 | 0.0232 | -0.0402 | -0.0652 | -0.0153 | -0.1134 | -0.2124 | -0.0144 | 0.3547 | 0.0016 |
| MeHg | WBC | TyGBMI | -0.0544 | -0.0852 | -0.0235 | -0.0181 | -0.0260 | -0.0101 | -0.0724 | -0.1040 | -0.0409 | 0.2494 | 0.0000 |
| MeHg | Neu | TyGBMI | -0.0637 | -0.0953 | -0.0320 | -0.0021 | -0.0049 | 0.0006 | -0.0658 | -0.0974 | -0.0342 | 0.0326 | **0.1208** |
| Pb | Mono | TyGWC | -0.3026 | -0.3696 | -0.2356 | -0.0237 | -0.0347 | -0.0126 | -0.3263 | -0.3936 | -0.2590 | 0.0726 | 0.0000 |
| Hg | WBC | TyGWC | -0.0827 | -0.1238 | -0.0416 | -0.0311 | -0.0434 | -0.0188 | -0.1137 | -0.1561 | -0.0713 | 0.2732 | 0.0000 |
| Hg | Lym | TyGWC | -0.0992 | -0.1418 | -0.0566 | -0.0031 | -0.0069 | 0.0006 | -0.1023 | -0.1448 | -0.0598 | 0.0306 | **0.0998** |
| Hg | Neu | TyGWC | -0.0980 | -0.1406 | -0.0555 | -0.0050 | -0.0091 | -0.0008 | -0.1030 | -0.1455 | -0.0605 | 0.0481 | 0.0205 |
| Cu | WBC | TyGWC | 0.3017 | 0.2216 | 0.3818 | 0.0814 | 0.0586 | 0.1042 | 0.3831 | 0.3020 | 0.4643 | 0.2124 | 0.0000 |
| Cu | Lym | TyGWC | 0.3926 | 0.3107 | 0.4744 | 0.0031 | -0.0034 | 0.0096 | 0.3957 | 0.3140 | 0.4773 | 0.0079 | **0.3492** |
| Cu | Mono | TyGWC | 0.3860 | 0.3045 | 0.4675 | 0.0359 | 0.0210 | 0.0508 | 0.4219 | 0.3404 | 0.5034 | 0.0851 | 0.0000 |
| Cu | Neu | TyGWC | 0.3865 | 0.3046 | 0.4685 | 0.0097 | 0.0008 | 0.0186 | 0.3962 | 0.3146 | 0.4778 | 0.0245 | 0.0329 |
| IHg | WBC | TyGWC | 0.0020 | -0.0507 | 0.0546 | -0.0260 | -0.0420 | -0.0100 | -0.0240 | -0.0788 | 0.0307 | 1.0000 | 0.0014 |
| EtHg | WBC | TyGWC | -0.0642 | -0.1690 | 0.0406 | -0.0519 | -0.0838 | -0.0201 | -0.1161 | -0.2251 | -0.0071 | 0.4471 | 0.0014 |
| EtHg | Lym | TyGWC | -0.1063 | -0.2152 | 0.0026 | -0.0057 | -0.0130 | 0.0016 | -0.1120 | -0.2209 | -0.0031 | 0.0509 | **0.1269** |
| MeHg | WBC | TyGWC | -0.0746 | -0.1081 | -0.0411 | -0.0232 | -0.0332 | -0.0132 | -0.0978 | -0.1324 | -0.0632 | 0.2375 | 0.0000 |
| MeHg | Lym | TyGWC | -0.0863 | -0.1211 | -0.0516 | -0.0025 | -0.0055 | 0.0005 | -0.0888 | -0.1235 | -0.0542 | 0.0284 | **0.1019** |
| MeHg | Neu | TyGWC | -0.0853 | -0.1200 | -0.0506 | -0.0042 | -0.0077 | -0.0007 | -0.0895 | -0.1242 | -0.0548 | 0.0472 | 0.0184 |
| Pb | Mono | TyGWHtR | -0.2855 | -0.3510 | -0.2199 | -0.0236 | -0.0346 | -0.0127 | -0.3091 | -0.3749 | -0.2433 | 0.0765 | 0.0000 |
| Hg | WBC | TyGWHtR | -0.0590 | -0.0991 | -0.0189 | -0.0315 | -0.0440 | -0.0191 | -0.0905 | -0.1320 | -0.0490 | 0.3486 | 0.0000 |
| Hg | Lym | TyGWHtR | -0.0763 | -0.1180 | -0.0346 | -0.0026 | -0.0062 | 0.0009 | -0.0789 | -0.1205 | -0.0373 | 0.0336 | **0.1445** |
| Hg | Neu | TyGWHtR | -0.0751 | -0.1168 | -0.0335 | -0.0044 | -0.0084 | -0.0005 | -0.0795 | -0.1211 | -0.0379 | 0.0555 | 0.0282 |
| Cu | WBC | TyGWHtR | 0.2781 | 0.2000 | 0.3562 | 0.0830 | 0.0601 | 0.1059 | 0.3611 | 0.2818 | 0.4404 | 0.2298 | 0.0000 |
| Cu | Lym | TyGWHtR | 0.3725 | 0.2925 | 0.4526 | 0.0021 | -0.0042 | 0.0084 | 0.3746 | 0.2948 | 0.4545 | 0.0056 | **0.5123** |
| Cu | Mono | TyGWHtR | 0.3643 | 0.2846 | 0.4440 | 0.0359 | 0.0212 | 0.0507 | 0.4002 | 0.3205 | 0.4799 | 0.0898 | 0.0000 |
| Cu | Neu | TyGWHtR | 0.3665 | 0.2863 | 0.4467 | 0.0082 | -0.0003 | 0.0168 | 0.3747 | 0.2949 | 0.4546 | 0.0220 | **0.0584** |
| Zn | WBC | TyGWHtR | 0.0057 | -0.0593 | 0.0707 | -0.0215 | -0.0416 | -0.0014 | -0.0158 | -0.0837 | 0.0521 | 1.0000 | 0.0360 |
| Zn | Lym | TyGWHtR | -0.0112 | -0.0792 | 0.0567 | -0.0036 | -0.0083 | 0.0011 | -0.0148 | -0.0827 | 0.0530 | 0.2443 | **0.1303** |
| EtHg | WBC | TyGWHtR | -0.0552 | -0.1572 | 0.0469 | -0.0524 | -0.0845 | -0.0203 | -0.1076 | -0.2141 | -0.0011 | 0.4872 | 0.0014 |
| MeHg | WBC | TyGWHtR | -0.0587 | -0.0914 | -0.0261 | -0.0235 | -0.0336 | -0.0135 | -0.0823 | -0.1161 | -0.0484 | 0.2861 | 0.0000 |
| MeHg | Lym | TyGWHtR | -0.0711 | -0.1051 | -0.0371 | -0.0021 | -0.0050 | 0.0008 | -0.0732 | -0.1071 | -0.0393 | 0.0287 | **0.1517** |
| MeHg | Neu | TyGWHtR | -0.0701 | -0.1040 | -0.0361 | -0.0037 | -0.0071 | -0.0004 | -0.0738 | -0.1077 | -0.0399 | 0.0508 | 0.0265 |

Table S15 Mediating effect and proportions of inflammation factors between heavy metal exposure and TyG-related indexes

(Adjusted for age, gender, race, educational level, marital status, PIR, weight, height, waist and BMI)

| Independent Variable | Intermediary Variable | Predictor Variable | Dierct Effects β (95% CI) | | | Indierct Effects β (95% CI) | | | Total Effects β (95% CI) | | | Mediated Proportion | P-value |
| --- | --- | --- | --- | --- | --- | --- | --- | --- | --- | --- | --- | --- | --- |
|  |  |  | Estimate | CI Lower | CI Upper | Estimate | CI Lower | CI Upper | Estimate | CI Lower | CI Upper |  |  |
| Pb | Eos | TyG | -0.0533 | -0.1016 | -0.0049 | 0.0048 | 0.0001 | 0.0094 | -0.0485 | -0.0967 | -0.0003 | -0.0986 | 0.0436 |
| Cd | WBC | TyG | 0.0091 | -0.0199 | 0.0381 | 0.0168 | 0.0105 | 0.0231 | 0.0259 | -0.0032 | 0.0550 | 0.6489 | 0.0000 |
| Cd | Mono | TyG | 0.0253 | -0.0035 | 0.0541 | 0.0067 | 0.0019 | 0.0115 | 0.0320 | 0.0029 | 0.0611 | 0.2102 | 0.0060 |
| Hg | WBC | TyG | 0.0318 | 0.0022 | 0.0613 | -0.0142 | -0.0204 | -0.0081 | 0.0175 | -0.0123 | 0.0473 | -0.8113 | 0.0000 |
| Hg | Neu | TyG | 0.0261 | -0.0038 | 0.0560 | 0.0005 | -0.0017 | 0.0027 | 0.0266 | -0.0033 | 0.0564 | 0.0185 | **0.6602** |
| Cu | Eos | TyG | -0.0150 | -0.0743 | 0.0443 | -0.0055 | -0.0111 | 0.0002 | -0.0205 | -0.0796 | 0.0386 | 0.2670 | **0.0565** |
| EtHg | WBC | TyG | 0.0147 | -0.0595 | 0.0889 | -0.0232 | -0.0380 | -0.0084 | -0.0085 | -0.0837 | 0.0668 | 1.0000 | 0.0022 |
| MeHg | WBC | TyG | 0.0188 | -0.0053 | 0.0428 | -0.0106 | -0.0155 | -0.0057 | 0.0082 | -0.0161 | 0.0325 | -1.2873 | 0.0000 |
| MeHg | Neu | TyG | 0.0153 | -0.0091 | 0.0397 | 0.0004 | -0.0014 | 0.0023 | 0.0157 | -0.0086 | 0.0400 | 0.0284 | **0.6425** |
| Pb | Mono | TyGBMI | -0.0203 | -0.0386 | -0.0021 | -0.0072 | -0.0104 | -0.0040 | -0.0275 | -0.0459 | -0.0092 | 0.2623 | 0.0000 |
| Hg | WBC | TyGBMI | 0.0114 | 0.0001 | 0.0227 | -0.0050 | -0.0073 | -0.0028 | 0.0064 | -0.0050 | 0.0177 | -0.7907 | 0.0000 |
| Hg | Neu | TyGBMI | 0.0094 | -0.0021 | 0.0208 | 0.0002 | -0.0006 | 0.0010 | 0.0096 | -0.0018 | 0.0209 | 0.0215 | **0.6300** |
| Cu | WBC | TyGBMI | -0.0281 | -0.0506 | -0.0055 | 0.0150 | 0.0100 | 0.0200 | -0.0131 | -0.0356 | 0.0095 | -1.0000 | 0.0000 |
| Cu | Mono | TyGBMI | -0.0168 | -0.0392 | 0.0057 | 0.0114 | 0.0071 | 0.0158 | -0.0053 | -0.0279 | 0.0172 | -1.0000 | 0.0000 |
| Cu | Neu | TyGBMI | -0.0141 | -0.0368 | 0.0086 | -0.0005 | -0.0027 | 0.0017 | -0.0146 | -0.0372 | 0.0080 | 0.0336 | **0.6602** |
| Zn | WBC | TyGBMI | 0.0531 | 0.0351 | 0.0711 | -0.0034 | -0.0067 | -0.0002 | 0.0497 | 0.0314 | 0.0679 | -0.0689 | 0.0402 |
| IHg | WBC | TyGBMI | 0.0094 | -0.0049 | 0.0236 | -0.0041 | -0.0068 | -0.0015 | 0.0053 | -0.0091 | 0.0197 | -0.7789 | 0.0024 |
| EtHg | WBC | TyGBMI | 0.0049 | -0.0235 | 0.0333 | -0.0082 | -0.0135 | -0.0029 | -0.0033 | -0.0320 | 0.0255 | 1.0000 | 0.0024 |
| MeHg | WBC | TyGBMI | 0.0069 | -0.0023 | 0.0162 | -0.0037 | -0.0055 | -0.0020 | 0.0032 | -0.0061 | 0.0125 | -1.0000 | 0.0000 |
| MeHg | Neu | TyGBMI | 0.0057 | -0.0036 | 0.0150 | 0.0002 | -0.0005 | 0.0009 | 0.0059 | -0.0034 | 0.0152 | 0.0315 | **0.6152** |
| Pb | Mono | TyGWC | -0.0290 | -0.0558 | -0.0021 | -0.0106 | -0.0153 | -0.0059 | -0.0395 | -0.0666 | -0.0125 | 0.2675 | 0.0000 |
| Hg | WBC | TyGWC | 0.0168 | 0.0002 | 0.0334 | -0.0077 | -0.0111 | -0.0043 | 0.0091 | -0.0077 | 0.0258 | -0.8518 | 0.0000 |
| Hg | Lym | TyGWC | 0.0123 | -0.0044 | 0.0291 | 0.0018 | 0.0002 | 0.0033 | 0.0141 | -0.0027 | 0.0309 | 0.1242 | 0.0313 |
| Hg | Neu | TyGWC | 0.0137 | -0.0031 | 0.0305 | 0.0003 | -0.0010 | 0.0015 | 0.0140 | -0.0028 | 0.0307 | 0.0180 | **0.6891** |
| Cu | WBC | TyGWC | -0.0405 | -0.0737 | -0.0074 | 0.0230 | 0.0155 | 0.0305 | -0.0175 | -0.0507 | 0.0156 | -1.0000 | 0.0000 |
| Cu | Lym | TyGWC | -0.0146 | -0.0479 | 0.0187 | -0.0034 | -0.0063 | -0.0004 | -0.0180 | -0.0512 | 0.0153 | 0.1868 | 0.0282 |
| Cu | Mono | TyGWC | -0.0231 | -0.0561 | 0.0100 | 0.0167 | 0.0103 | 0.0232 | -0.0063 | -0.0395 | 0.0268 | -1.0000 | 0.0000 |
| Cu | Neu | TyGWC | -0.0193 | -0.0526 | 0.0141 | -0.0006 | -0.0038 | 0.0026 | -0.0199 | -0.0531 | 0.0134 | 0.0302 | **0.7145** |
| IHg | WBC | TyGWC | 0.0155 | -0.0054 | 0.0364 | -0.0063 | -0.0104 | -0.0023 | 0.0092 | -0.0120 | 0.0304 | -0.6875 | 0.0023 |
| EtHg | WBC | TyGWC | 0.0076 | -0.0342 | 0.0493 | -0.0126 | -0.0207 | -0.0045 | -0.0050 | -0.0473 | 0.0373 | 1.0000 | 0.0023 |
| EtHg | Lym | TyGWC | -0.0043 | -0.0465 | 0.0379 | 0.0028 | -0.0004 | 0.0061 | -0.0015 | -0.0437 | 0.0408 | -1.0000 | **0.0883** |
| MeHg | WBC | TyGWC | 0.0098 | -0.0038 | 0.0233 | -0.0057 | -0.0084 | -0.0030 | 0.0040 | -0.0096 | 0.0177 | -1.0000 | 0.0000 |
| MeHg | Lym | TyGWC | 0.0068 | -0.0069 | 0.0205 | 0.0014 | 0.0001 | 0.0027 | 0.0083 | -0.0054 | 0.0219 | 0.1739 | 0.0304 |
| MeHg | Neu | TyGWC | 0.0079 | -0.0058 | 0.0216 | 0.0002 | -0.0008 | 0.0013 | 0.0081 | -0.0056 | 0.0218 | 0.0282 | **0.6713** |
| Pb | Mono | TyGWHtR | -0.0304 | -0.0572 | -0.0036 | -0.0104 | -0.0151 | -0.0057 | -0.0408 | -0.0677 | -0.0138 | 0.2551 | 0.0000 |
| Hg | WBC | TyGWHtR | 0.0176 | 0.0010 | 0.0342 | -0.0073 | -0.0106 | -0.0041 | 0.0103 | -0.0064 | 0.0270 | -0.7152 | 0.0000 |
| Hg | Lym | TyGWHtR | 0.0132 | -0.0035 | 0.0299 | 0.0018 | 0.0002 | 0.0035 | 0.0151 | -0.0016 | 0.0318 | 0.1228 | 0.0253 |
| Hg | Neu | TyGWHtR | 0.0146 | -0.0022 | 0.0313 | 0.0004 | -0.0009 | 0.0016 | 0.0149 | -0.0018 | 0.0317 | 0.0247 | **0.5573** |
| Cu | WBC | TyGWHtR | -0.0424 | -0.0755 | -0.0093 | 0.0220 | 0.0146 | 0.0293 | -0.0205 | -0.0535 | 0.0126 | -1.0000 | 0.0000 |
| Cu | Lym | TyGWHtR | -0.0172 | -0.0503 | 0.0160 | -0.0035 | -0.0066 | -0.0005 | -0.0207 | -0.0538 | 0.0124 | 0.1704 | 0.0228 |
| Cu | Mono | TyGWHtR | -0.0259 | -0.0588 | 0.0071 | 0.0165 | 0.0101 | 0.0228 | -0.0094 | -0.0424 | 0.0236 | -1.0000 | 0.0000 |
| Cu | Neu | TyGWHtR | -0.0218 | -0.0551 | 0.0115 | -0.0009 | -0.0041 | 0.0023 | -0.0227 | -0.0558 | 0.0104 | 0.0394 | **0.5857** |
| Zn | WBC | TyGWHtR | 0.0806 | 0.0542 | 0.1070 | -0.0050 | -0.0098 | -0.0002 | 0.0756 | 0.0489 | 0.1023 | -0.0661 | 0.0403 |
| Zn | Lym | TyGWHtR | 0.0747 | 0.0480 | 0.1014 | 0.0019 | -0.0002 | 0.0040 | 0.0766 | 0.0499 | 0.1033 | 0.0252 | **0.0709** |
| EtHg | WBC | TyGWHtR | 0.0055 | -0.0362 | 0.0471 | -0.0119 | -0.0197 | -0.0042 | -0.0065 | -0.0486 | 0.0357 | 1.0000 | 0.0025 |
| MeHg | WBC | TyGWHtR | 0.0103 | -0.0033 | 0.0238 | -0.0054 | -0.0080 | -0.0029 | 0.0048 | -0.0088 | 0.0184 | -1.0000 | 0.0000 |
| MeHg | Lym | TyGWHtR | 0.0073 | -0.0063 | 0.0210 | 0.0015 | 0.0002 | 0.0028 | 0.0089 | -0.0048 | 0.0225 | 0.1713 | 0.0247 |
| MeHg | Neu | TyGWHtR | 0.0084 | -0.0053 | 0.0221 | 0.0003 | -0.0007 | 0.0014 | 0.0087 | -0.0049 | 0.0224 | 0.0380 | **0.5403** |


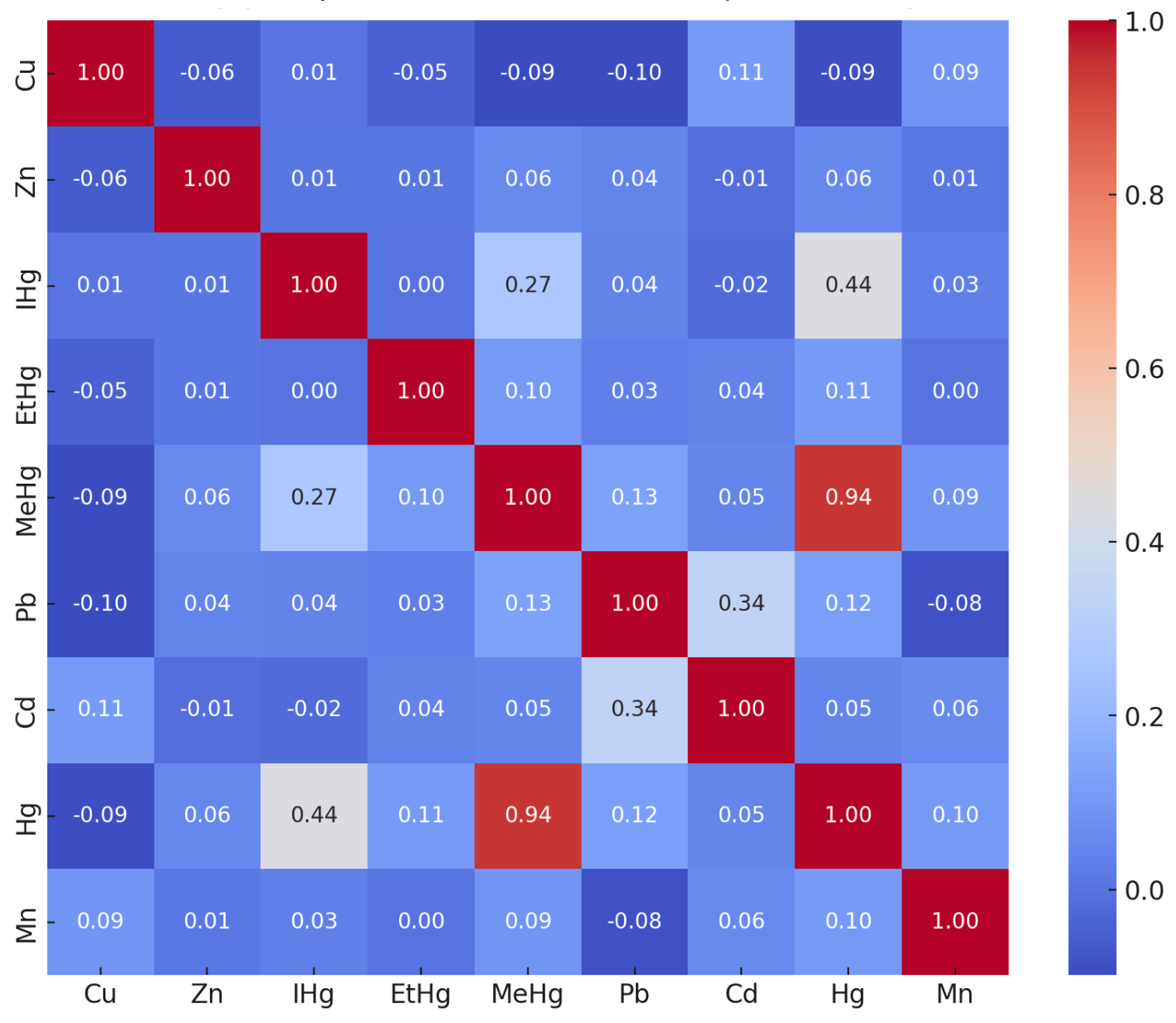


Figure S1 Pearson correlations among 9 heavy metals


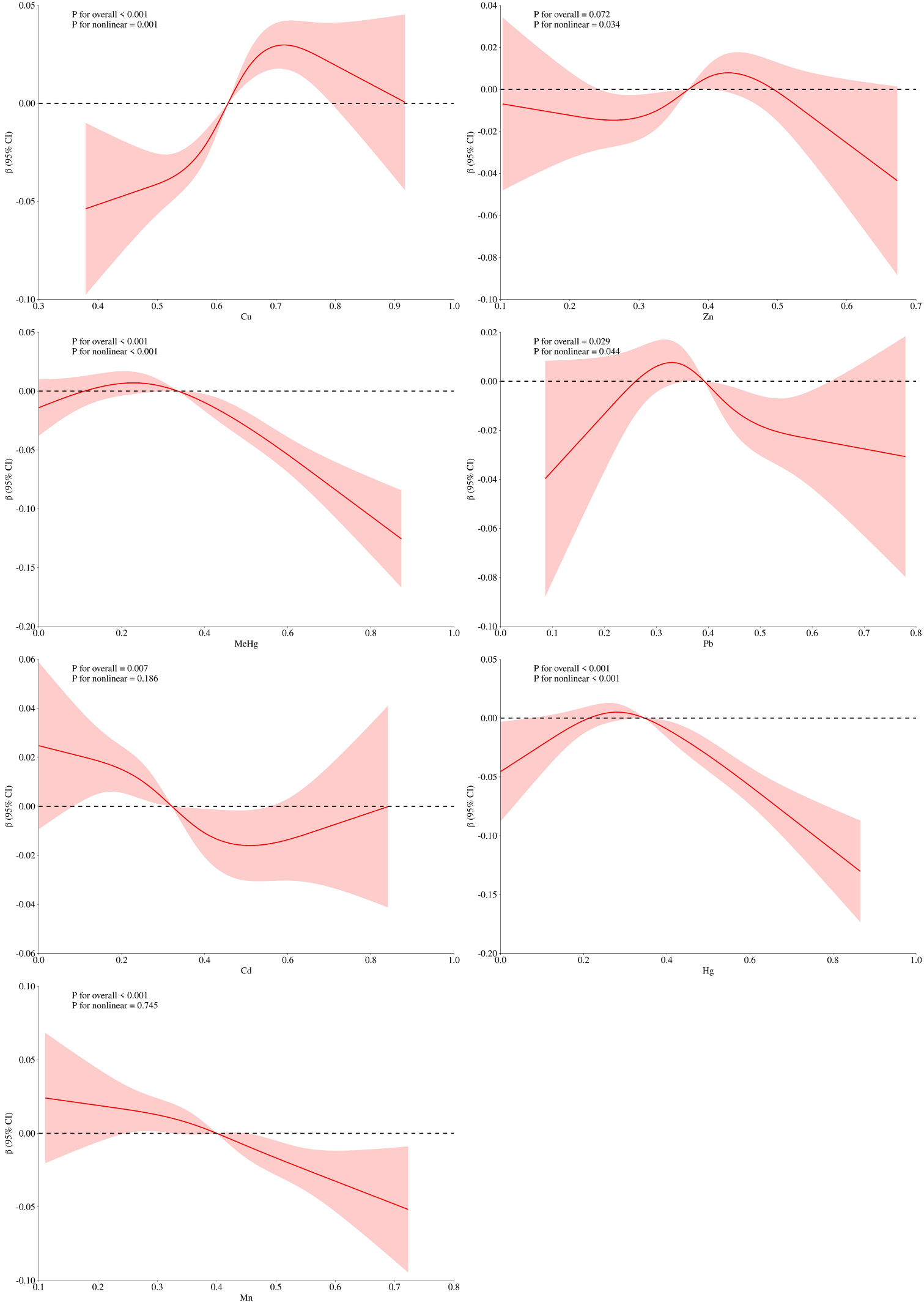


Figure S2 Weighted restricted cubic spline curve describing the non-linear association between heavy metal exposure and TyGWC index (Unadjusted)


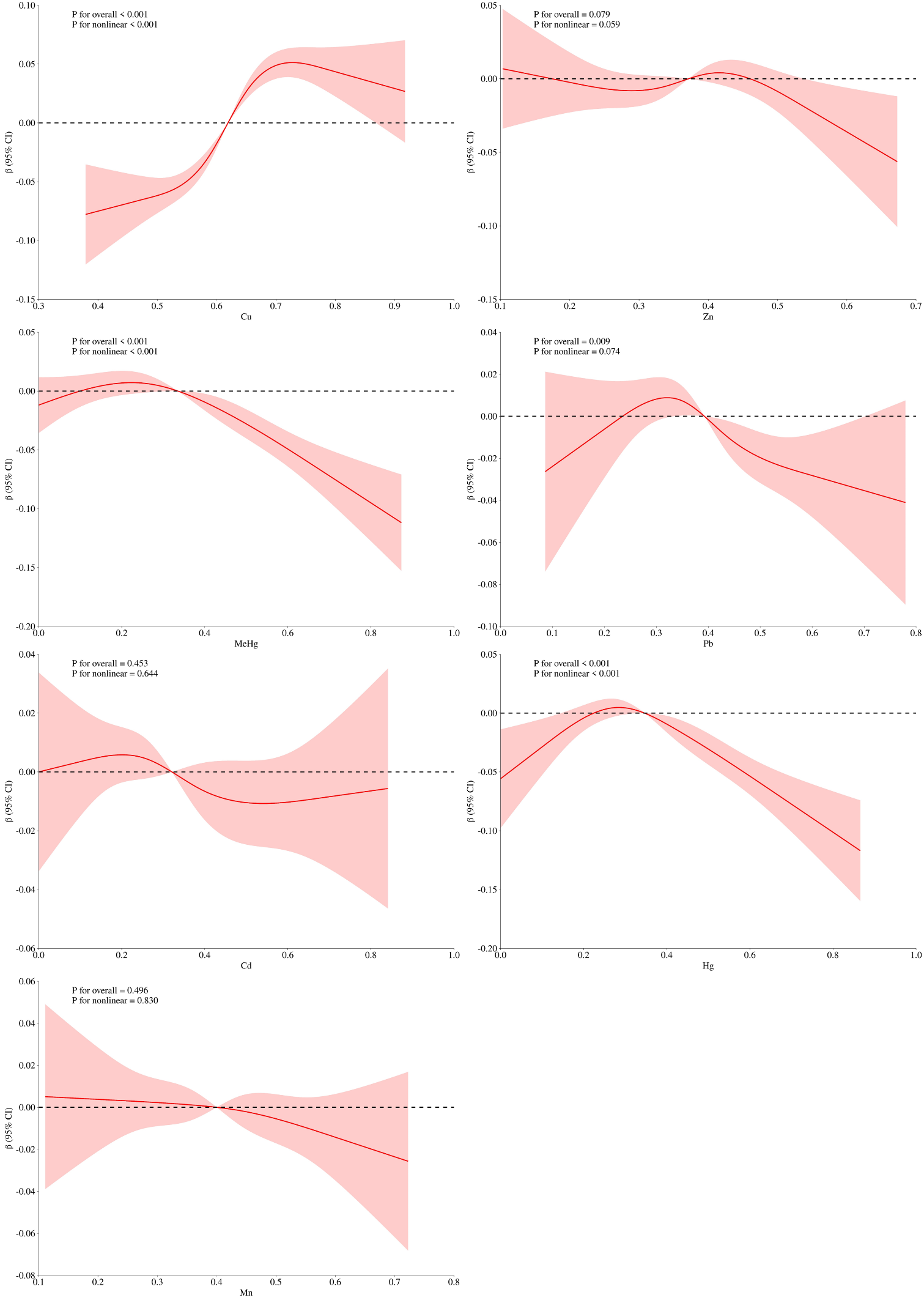


Figure S3 Weighted restricted cubic spline curve describing the non-linear association between heavy metal exposure and TyGWHtR index (Unadjusted)


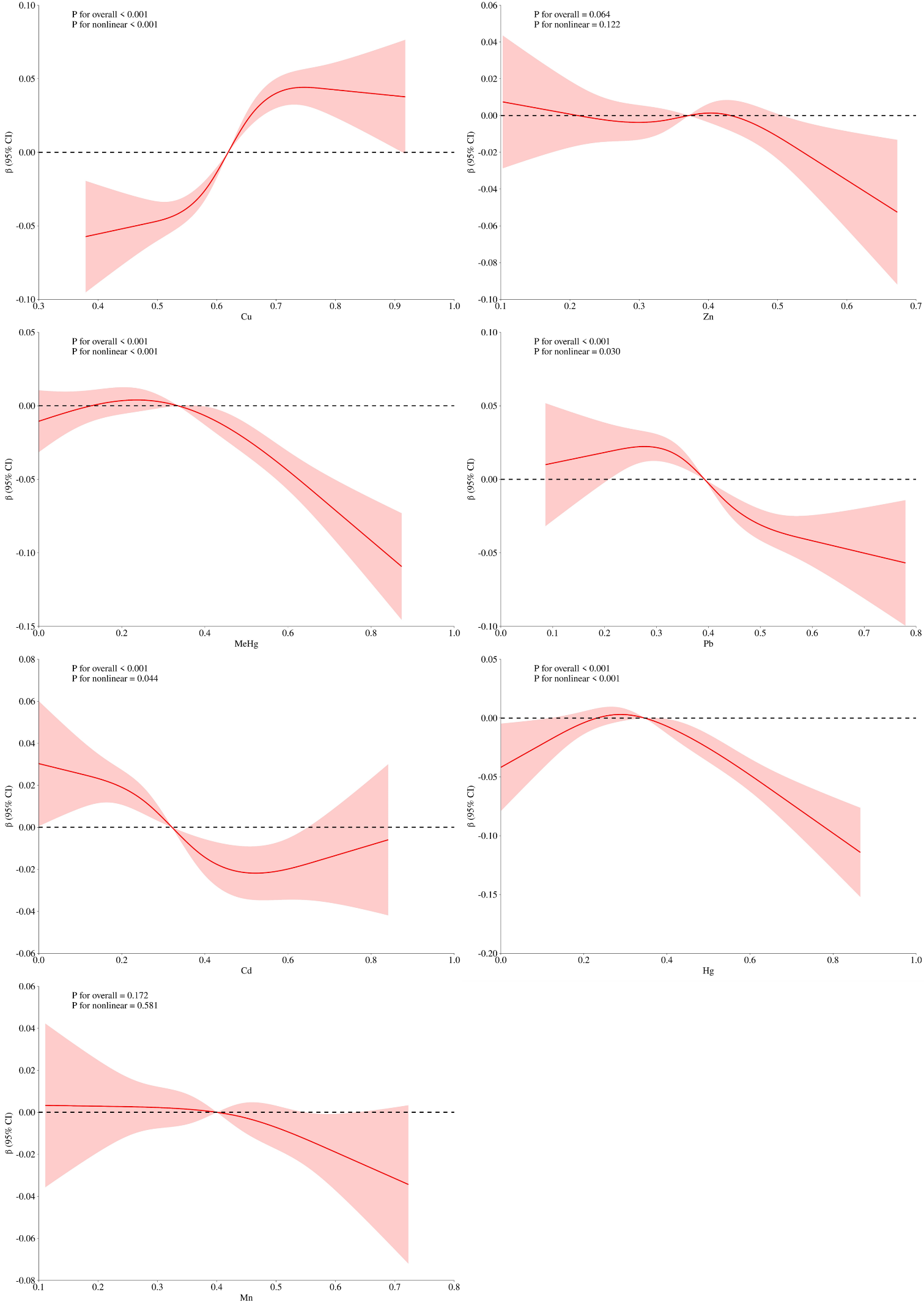


Figure S4 Weighted restricted cubic spline curve describing the non-linear association between heavy metal exposure and TyGBMI index (Unadjusted)


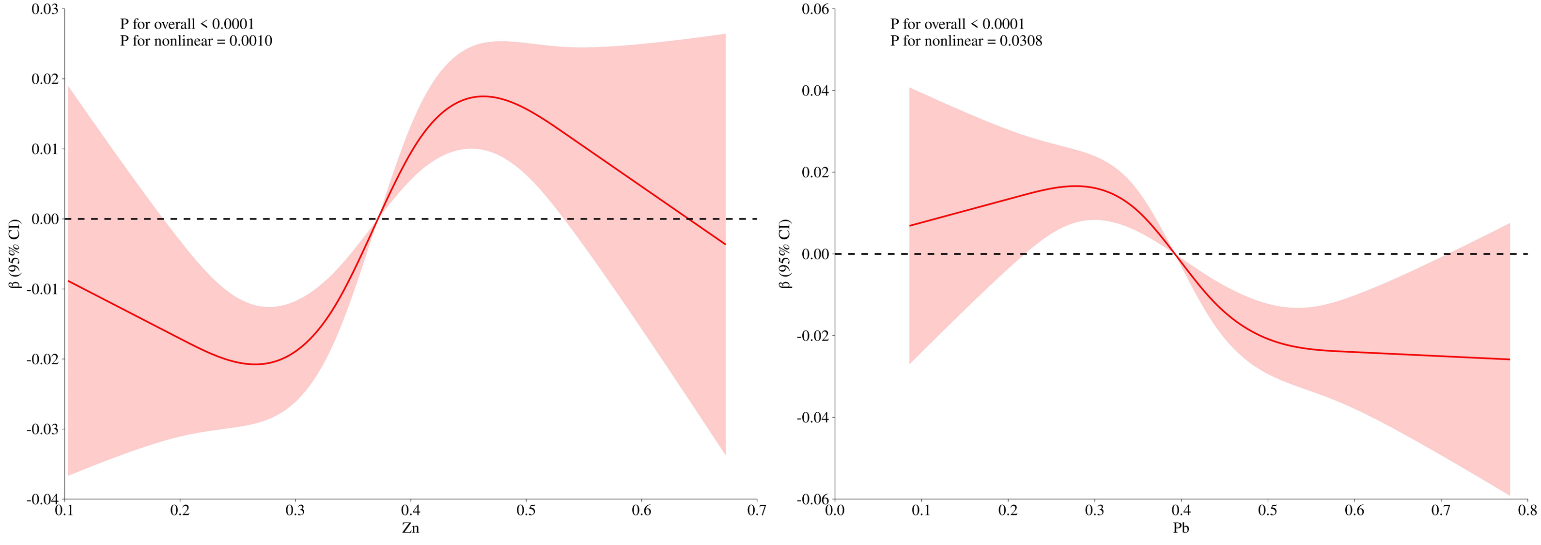


Figure S5 Weighted restricted cubic spline curve describing the non-linear association between heavy metal exposure and TyG index (Adjusted for age, gender, race, educational level, marital status and PIR)


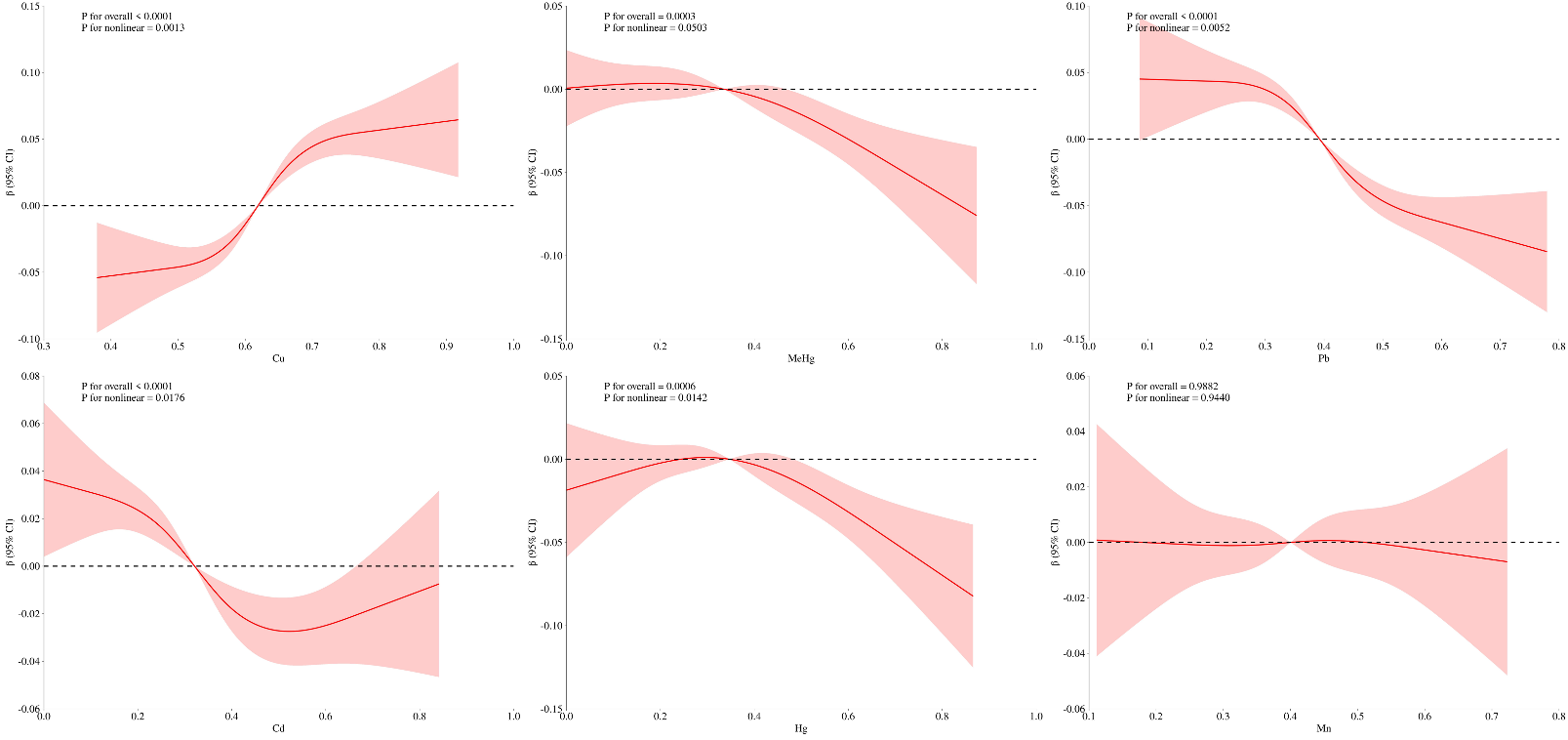


Figure S6 Weighted restricted cubic spline curve describing the non-linear association between heavy metal exposure and TyGWC index (Adjusted for age, gender, race, educational level, marital status and PIR)


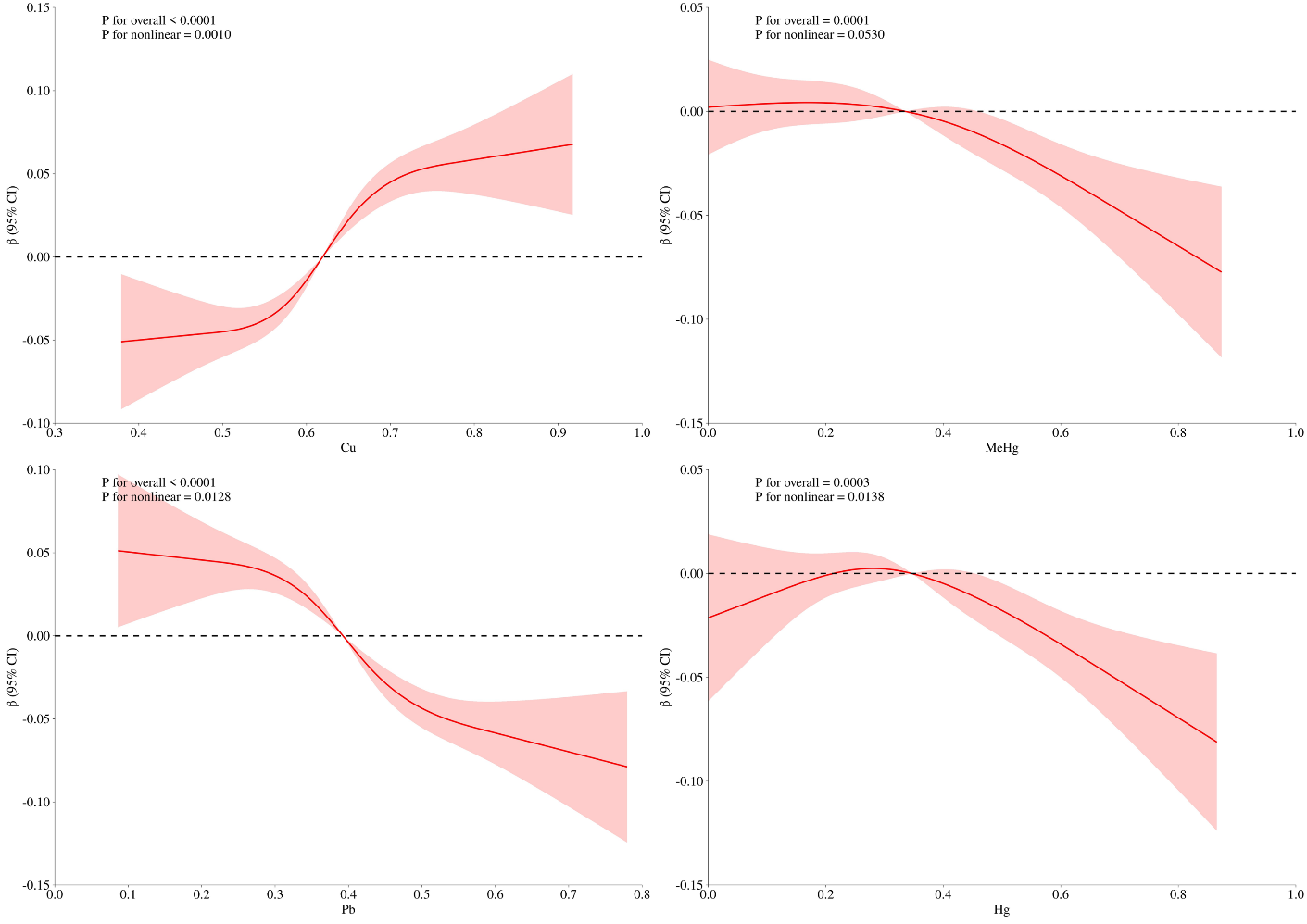


Figure S7 Weighted restricted cubic spline curve describing the non-linear association between heavy metal exposure and TyGWHtR index (Adjusted for age, gender, race, educational level, marital status and PIR)


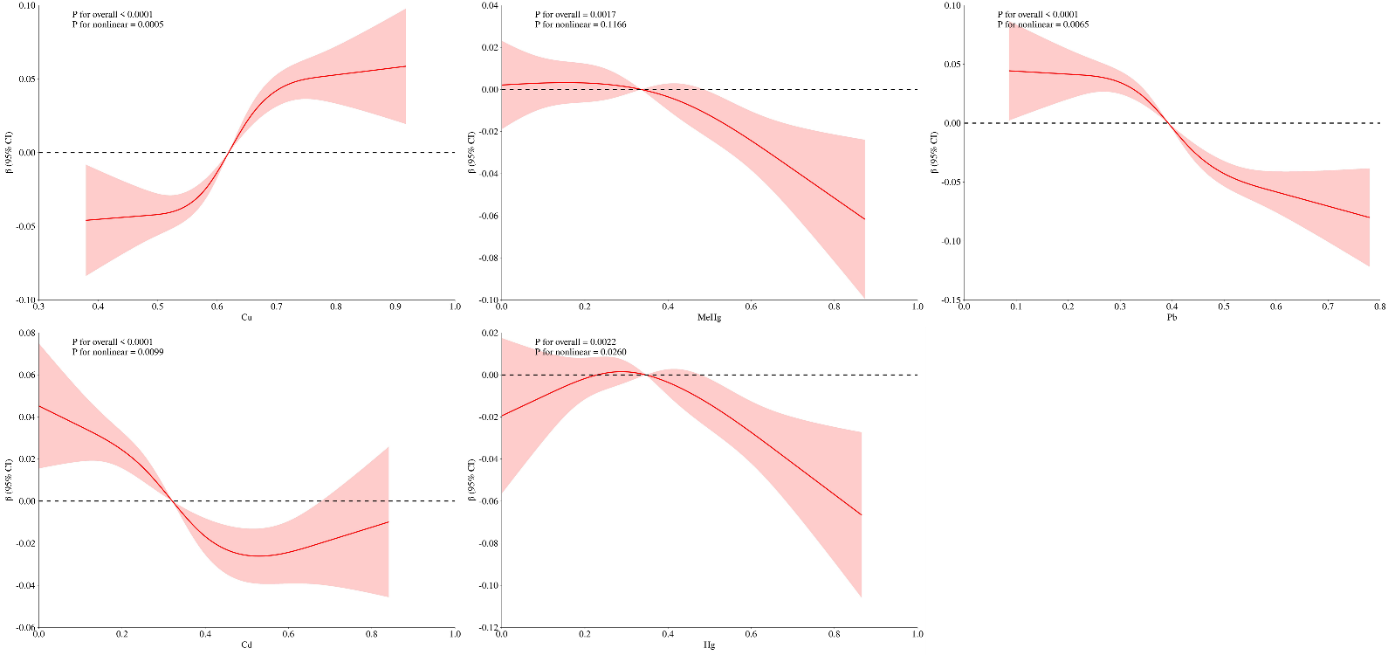


Figure S8 Weighted restricted cubic spline curve describing the non-linear association between heavy metal exposure and TyGBMI index (Adjusted for age, gender, race, educational level, marital status and PIR)


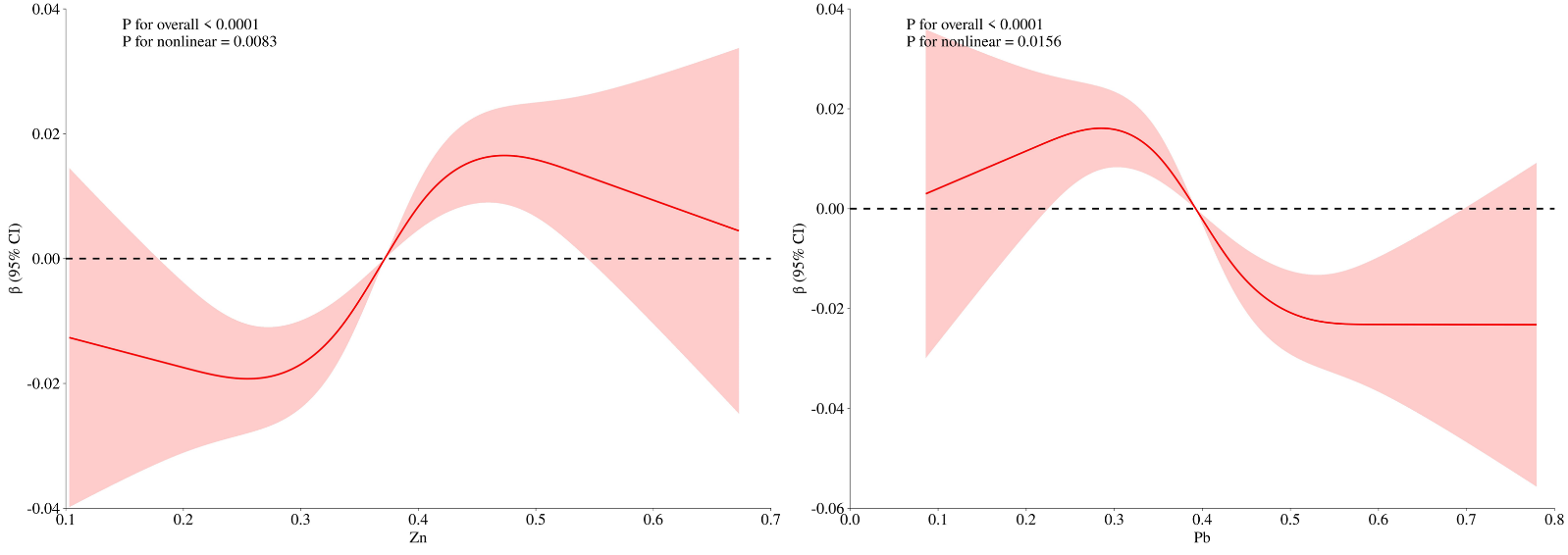


Figure S9 Weighted restricted cubic spline curve describing the non-linear association between heavy metal exposure and TyG index (Adjusted for age, gender, race, educational level, marital status, PIR, WBC, Lym, Mono, Neu, Eos and Baso)


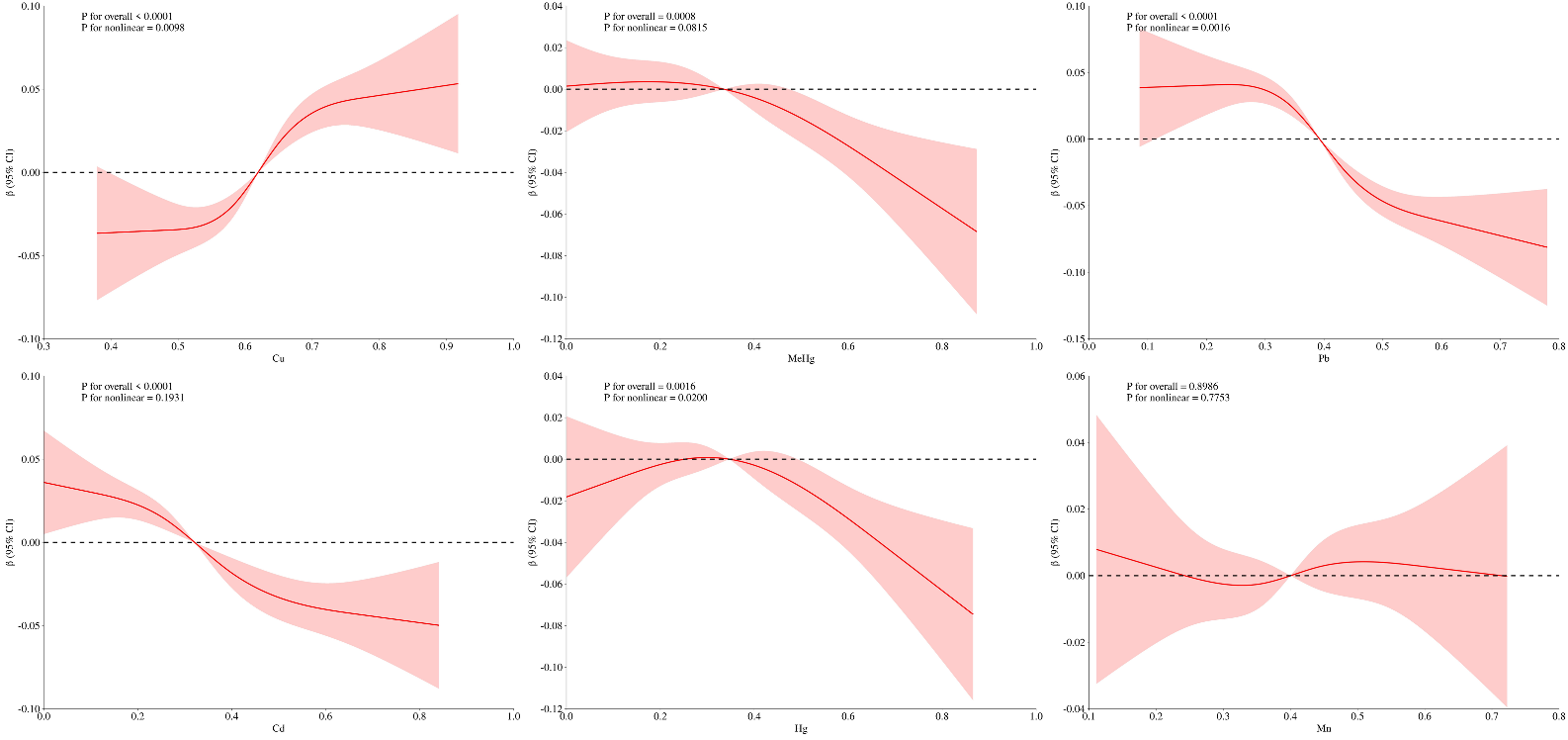


Figure S10 Weighted restricted cubic spline curve describing the non-linear association between heavy metal exposure and TyGWC index (Adjusted for age, gender, race, educational level, marital status, PIR, WBC, Lym, Mono, Neu, Eos and Baso)


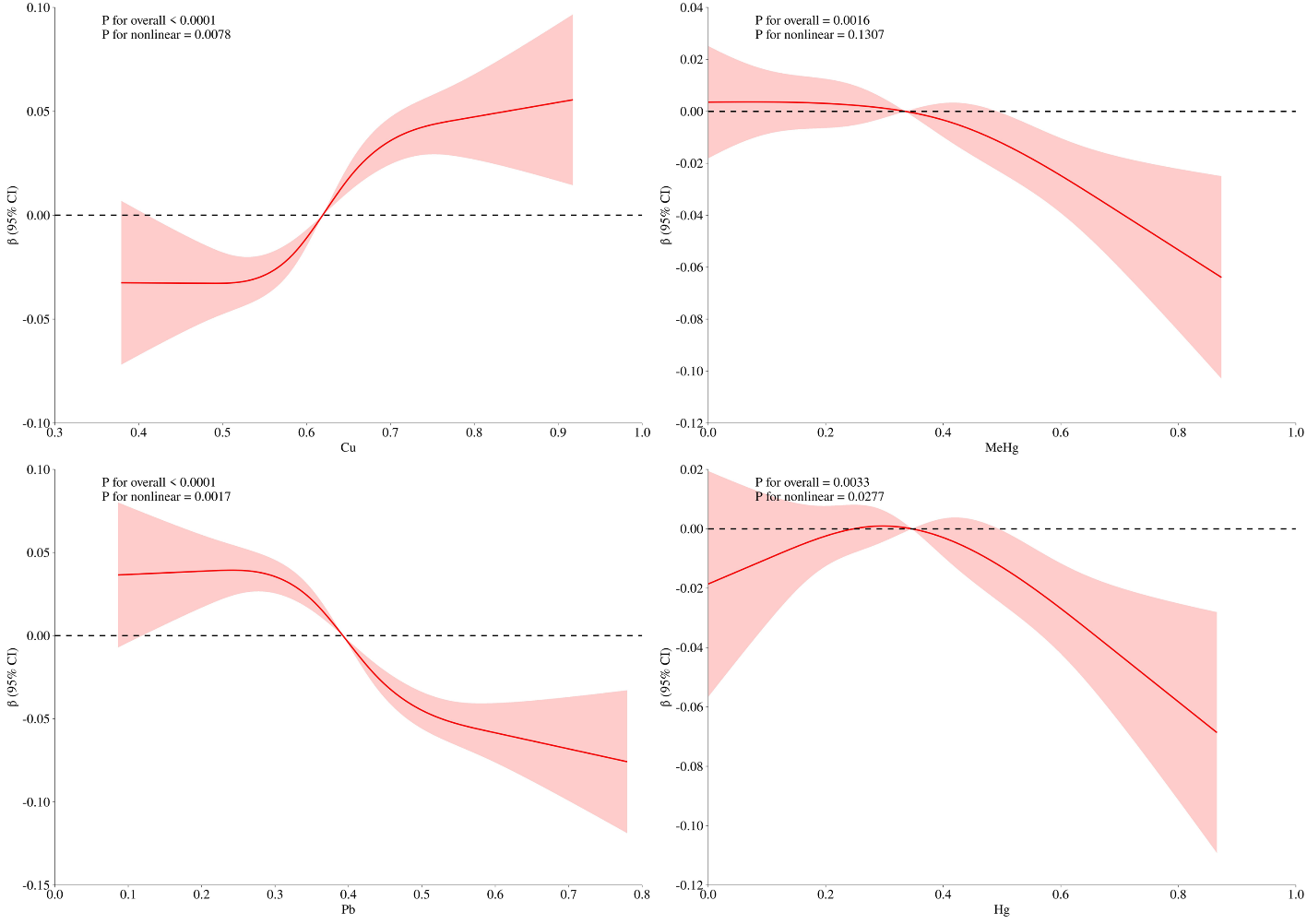


Figure S11 Weighted restricted cubic spline curve describing the non-linear association between heavy metal exposure and TyGWHtR index (Adjusted for age, gender, race, educational level, marital status, PIR, WBC, Lym, Mono, Neu, Eos and Baso)


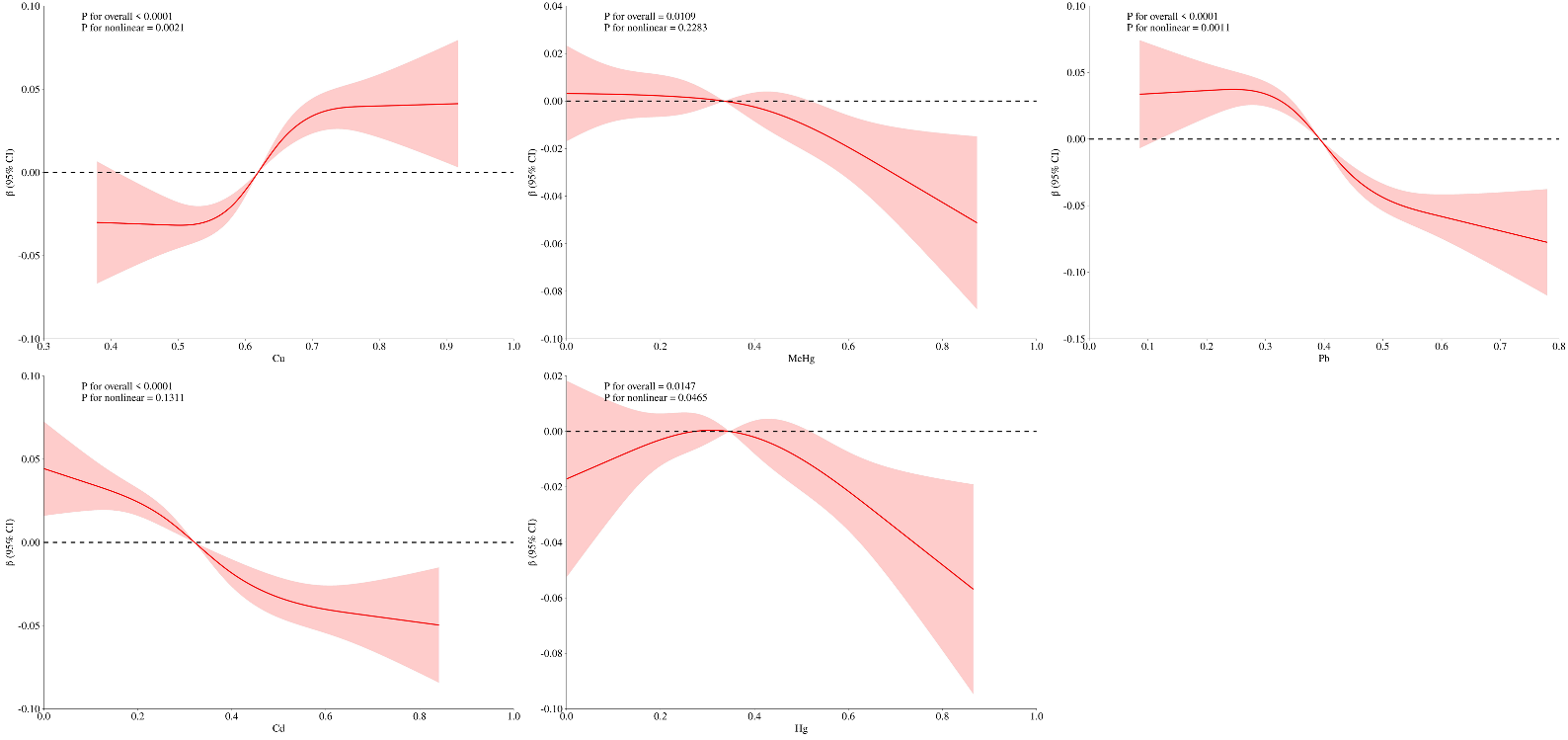


Figure S12 Weighted restricted cubic spline curve describing the non-linear association between heavy metal exposure and TyGBMI index (Adjusted for age, gender, race, educational level, marital status, PIR, WBC, Lym, Mono, Neu, Eos and Baso)


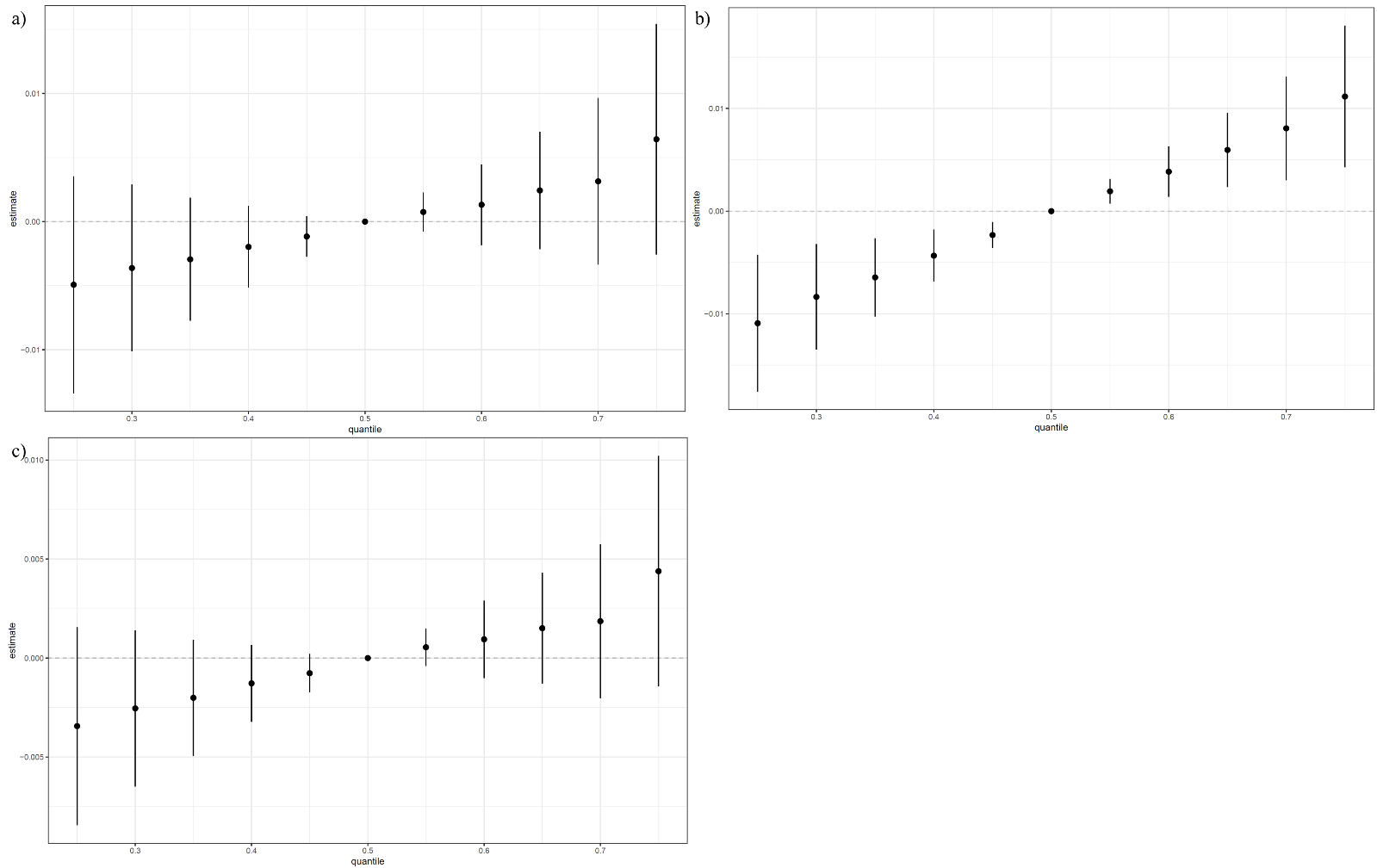


Figure S13 Overall effect of heavy metals mixtures on the TyG in BKMR model where all heavy metals at specific percentiles were compared to their 50th percentile.

*Note: a) was adjusted for age, gender, race, educational level, marital status and PIR; b) was adjusted for age, gender, race, educational level, marital status, PIR, weight, height, waist and BMI; c) was adjusted for age, gender, race, educational level, marital status, PIR, weight, height, waist, BMI, WBC, Lym, Mono, Neu, Eos and Baso.*


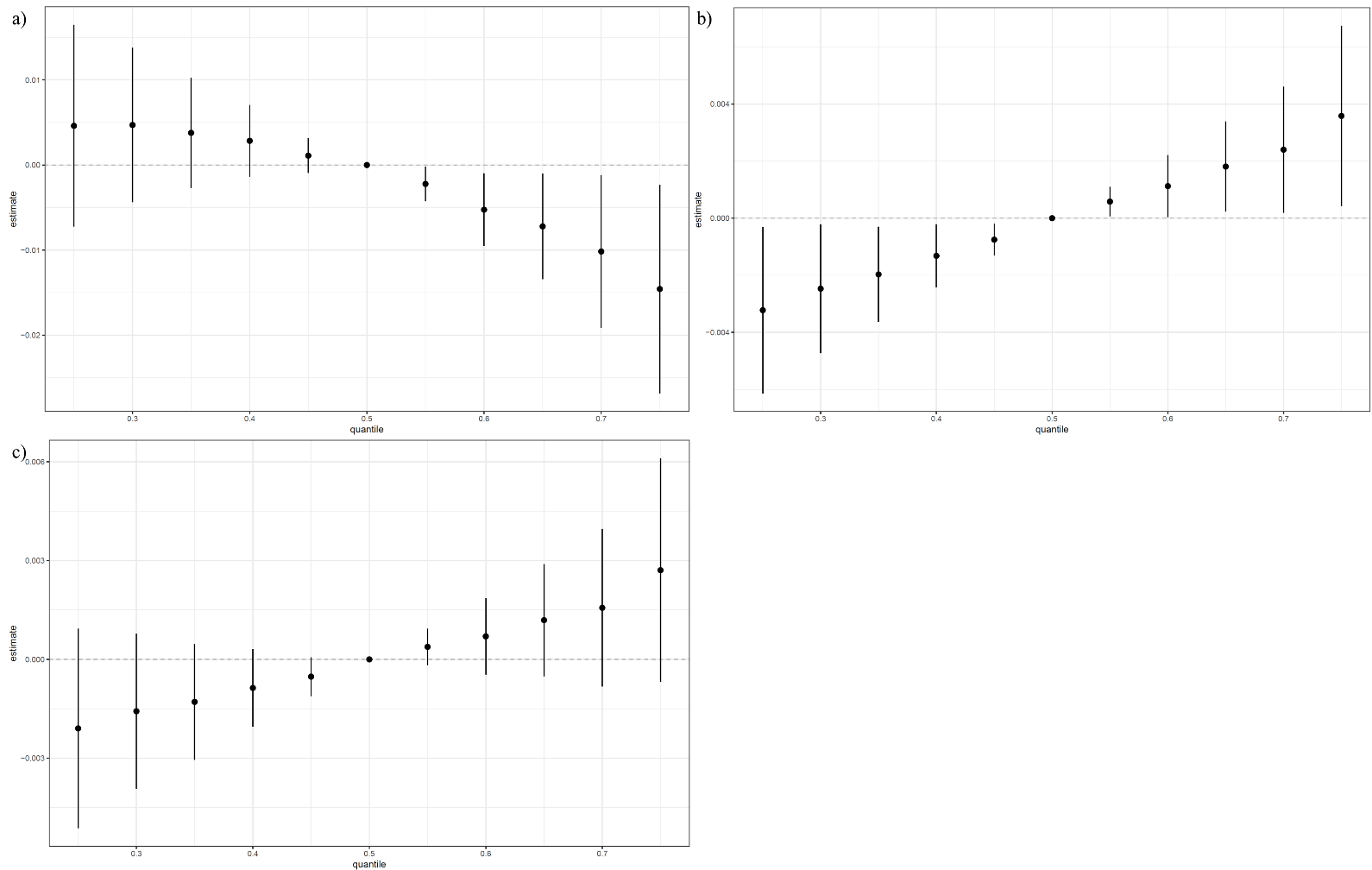


Figure S14 Overall effect of heavy metals mixtures on the TyGWC in BKMR model where all heavy metals at specific percentiles were compared to their 50th percentile.

*Note: a) was adjusted for age, gender, race, educational level, marital status and PIR; b) was adjusted for age, gender, race, educational level, marital status, PIR, weight, height, waist and BMI; c) was adjusted for age, gender, race, educational level, marital status, PIR, weight, height, waist, BMI, WBC, Lym, Mono, Neu, Eos and Baso.*


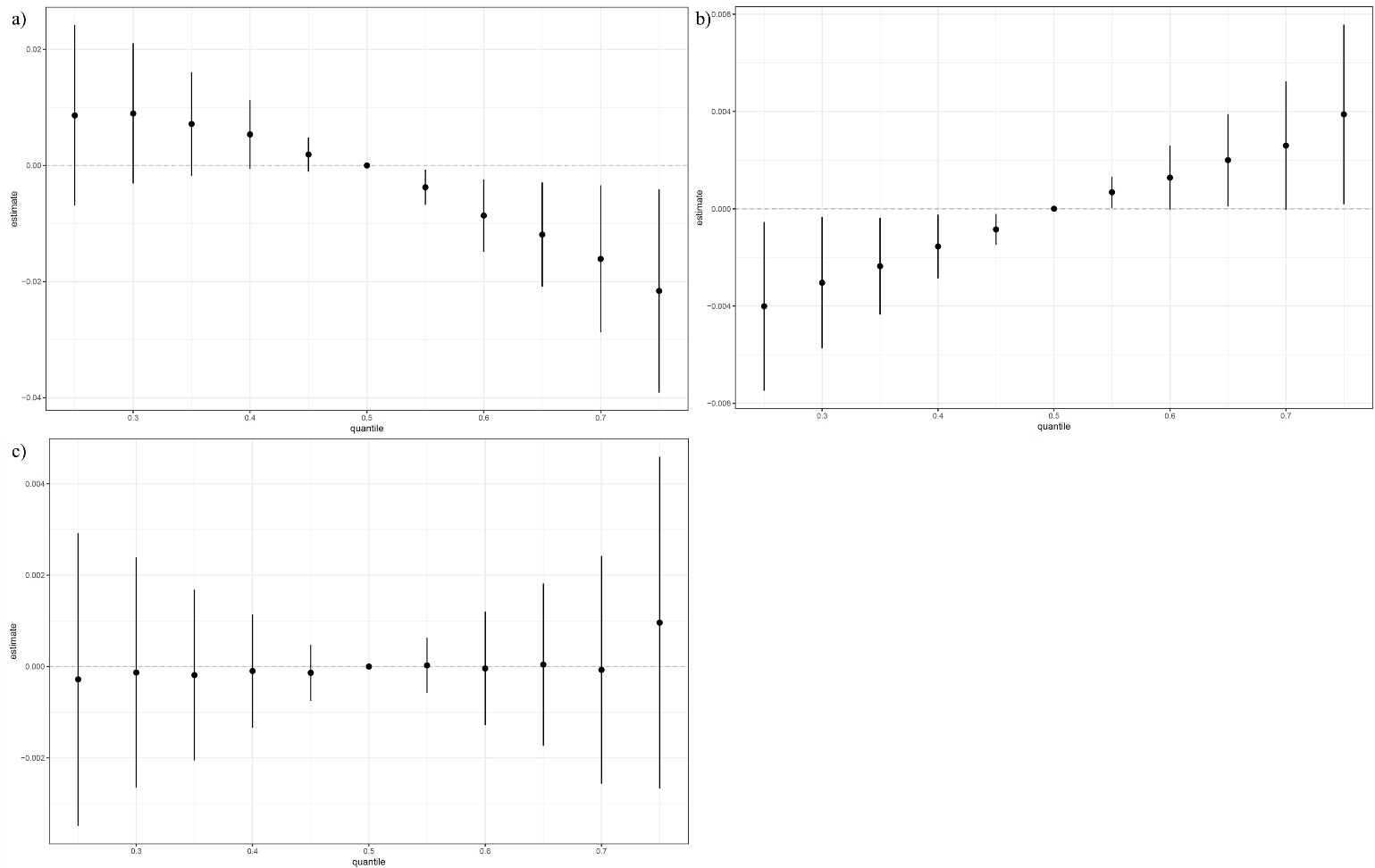


Figure S15 Overall effect of heavy metals mixtures on the TyGWhtR in BKMR model where all heavy metals at specific percentiles were compared to their 50th percentile.

*Note: a) was adjusted for age, gender, race, educational level, marital status and PIR; b) was adjusted for age, gender, race, educational level, marital status, PIR, weight, height, waist and BMI; c) was adjusted for age, gender, race, educational level, marital status, PIR, weight, height, waist, BMI, WBC, Lym, Mono, Neu, Eos and Baso.*


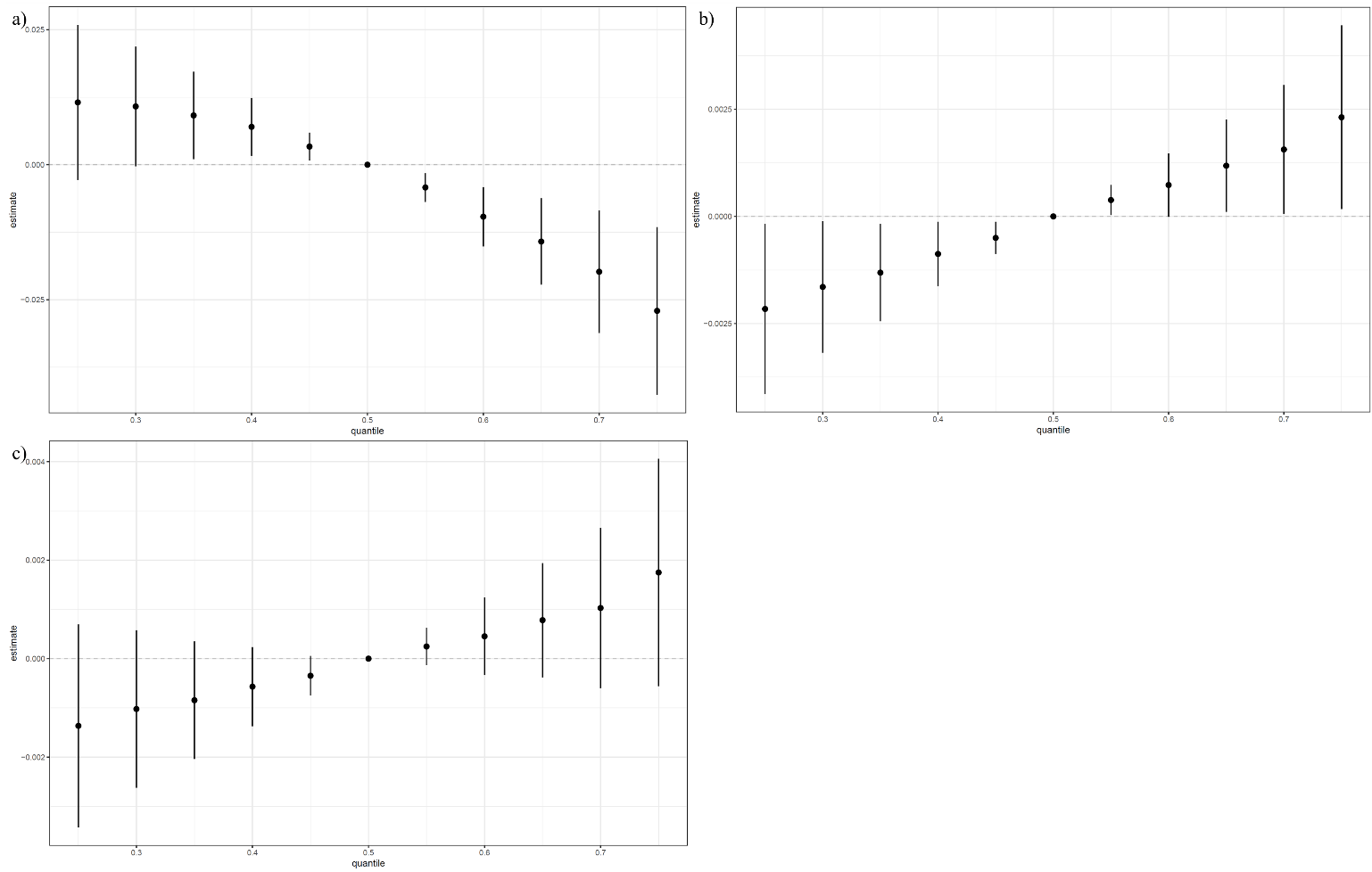


Figure S16 Overall effect of heavy metals mixtures on the TyGBMI in BKMR model where all heavy metals at specific percentiles were compared to their 50th percentile.

*Note: a) was adjusted for age, gender, race, educational level, marital status and PIR; b) was adjusted for age, gender, race, educational level, marital status, PIR, weight, height, waist and BMI; c) was adjusted for age, gender, race, educational level, marital status, PIR, weight, height, waist, BMI, WBC, Lym, Mono, Neu, Eos and Baso.*


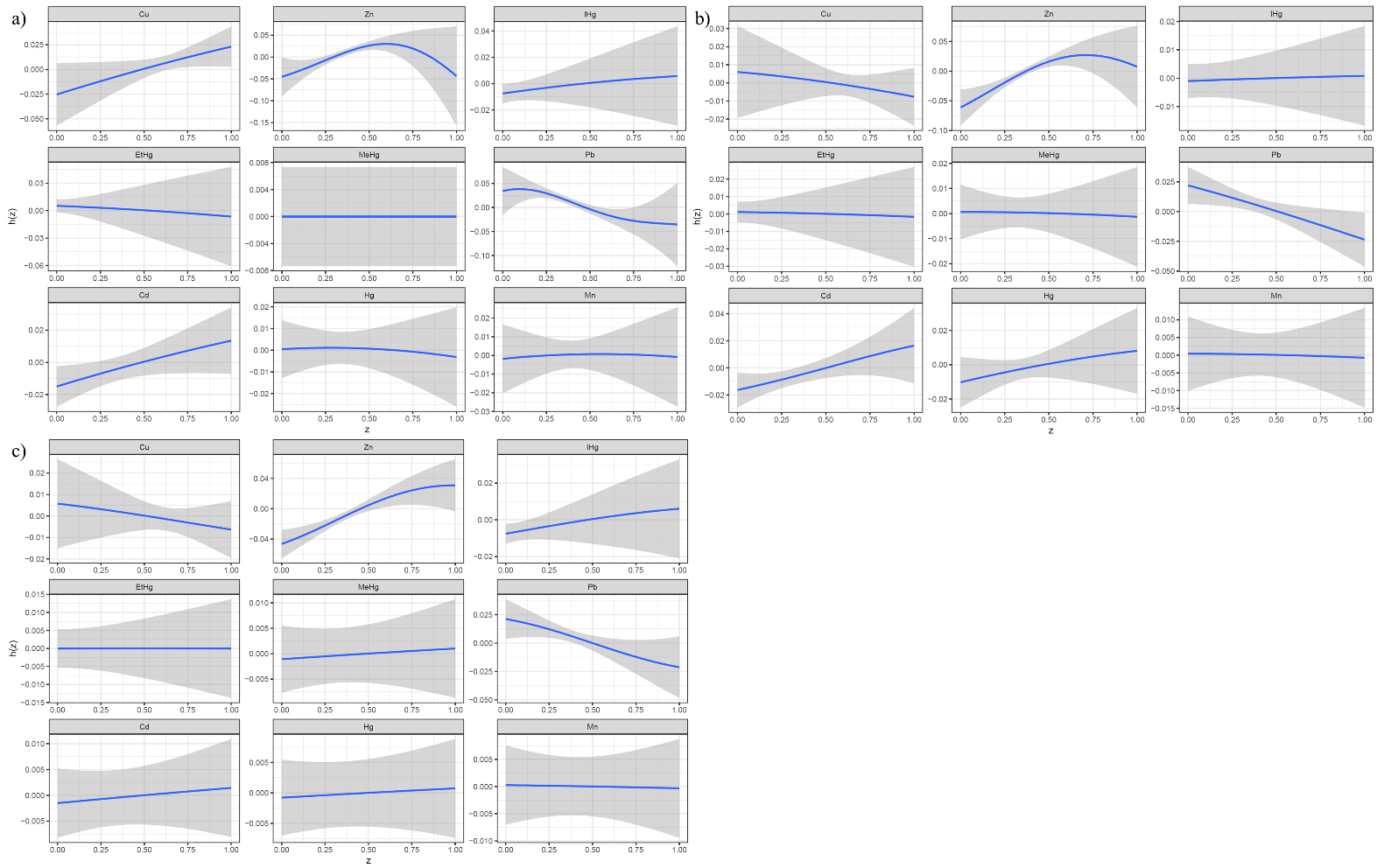


Figure S17 Univariate exposure–response function between each heavy metal and the TyG when the other heavy metals were fixed at 50th percentiles.

*Note: a) was adjusted for age, gender, race, educational level, marital status and PIR; b) was adjusted for age, gender, race, educational level, marital status, PIR, weight, height, waist and BMI; c) was adjusted for age, gender, race, educational level, marital status, PIR, weight, height, waist, BMI, WBC, Lym, Mono, Neu, Eos and Baso.*


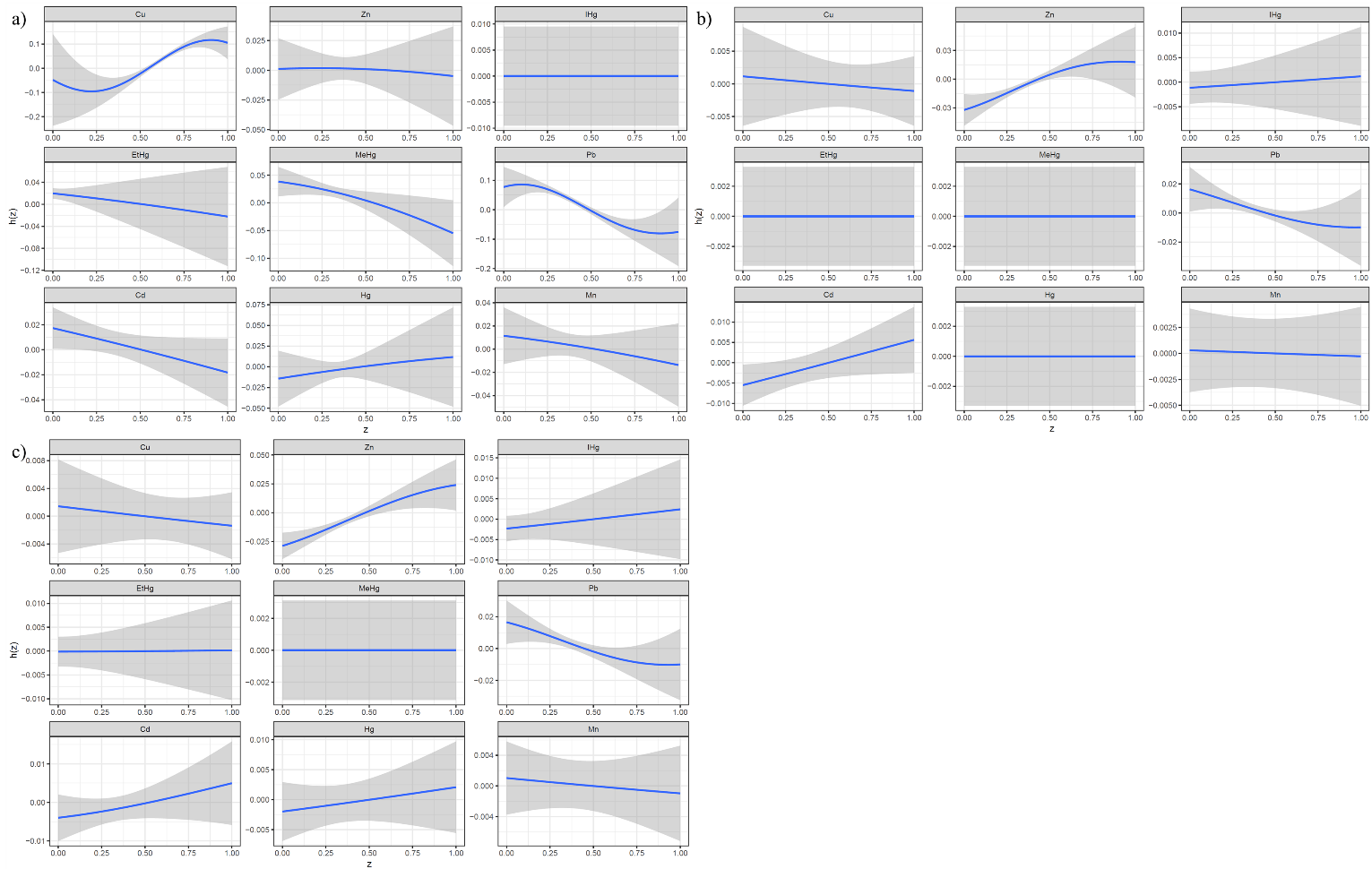


Figure S18 Univariate exposure–response function between each heavy metal and the TyGWC when the other heavy metals were fixed at 50th percentiles.

*Note: a) was adjusted for age, gender, race, educational level, marital status and PIR; b) was adjusted for age, gender, race, educational level, marital status, PIR, weight, height, waist and BMI; c) was adjusted for age, gender, race, educational level, marital status, PIR, weight, height, waist, BMI, WBC, Lym, Mono, Neu, Eos and Baso.*


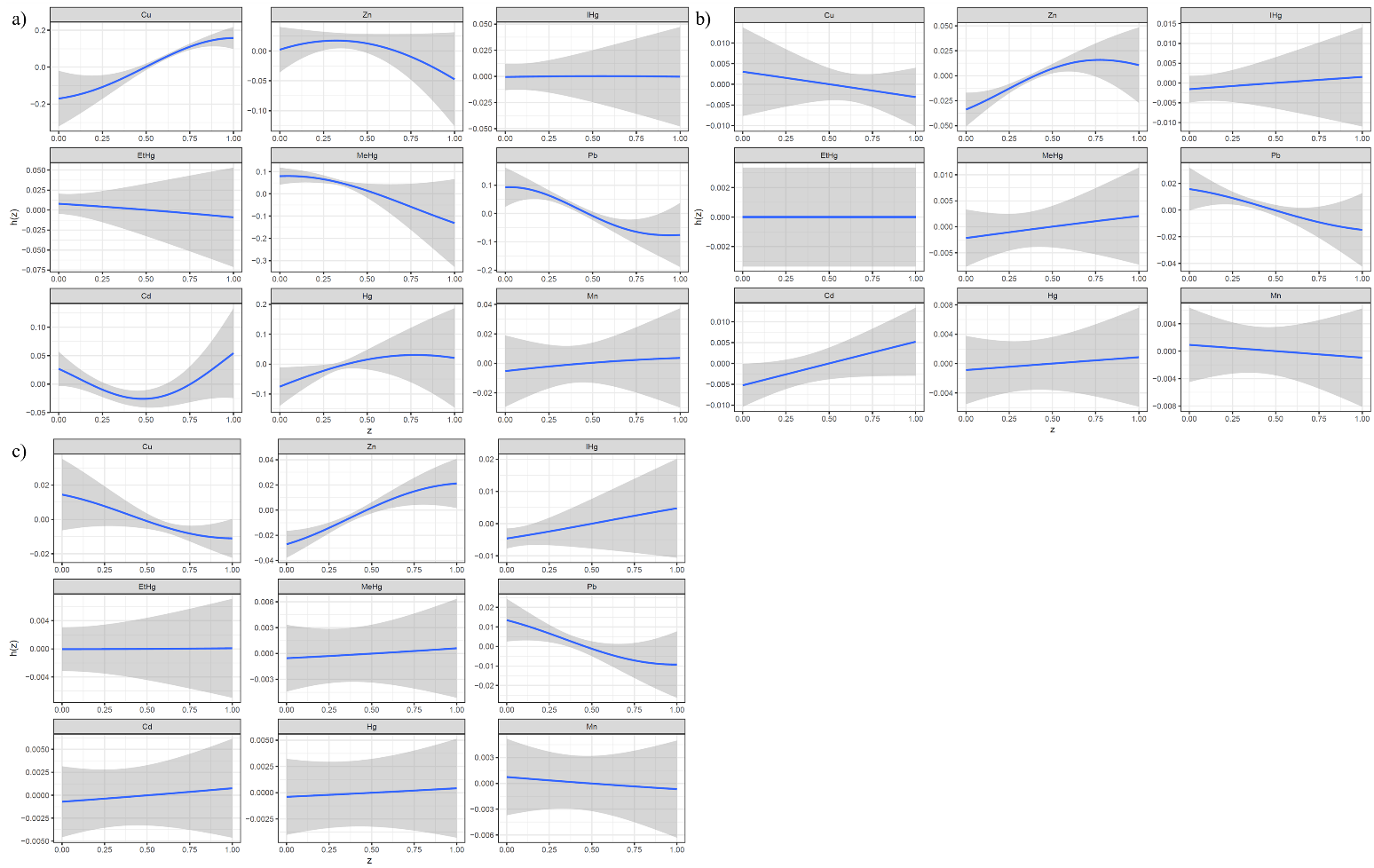


Figure S19 Univariate exposure–response function between each heavy metal and the TyGWHtR when the other heavy metals were fixed at 50th percentiles.

*Note: a) was adjusted for age, gender, race, educational level, marital status and PIR; b) was adjusted for age, gender, race, educational level, marital status, PIR, weight, height, waist and BMI; c) was adjusted for age, gender, race, educational level, marital status, PIR, weight, height, waist, BMI, WBC, Lym, Mono, Neu, Eos and Baso.*


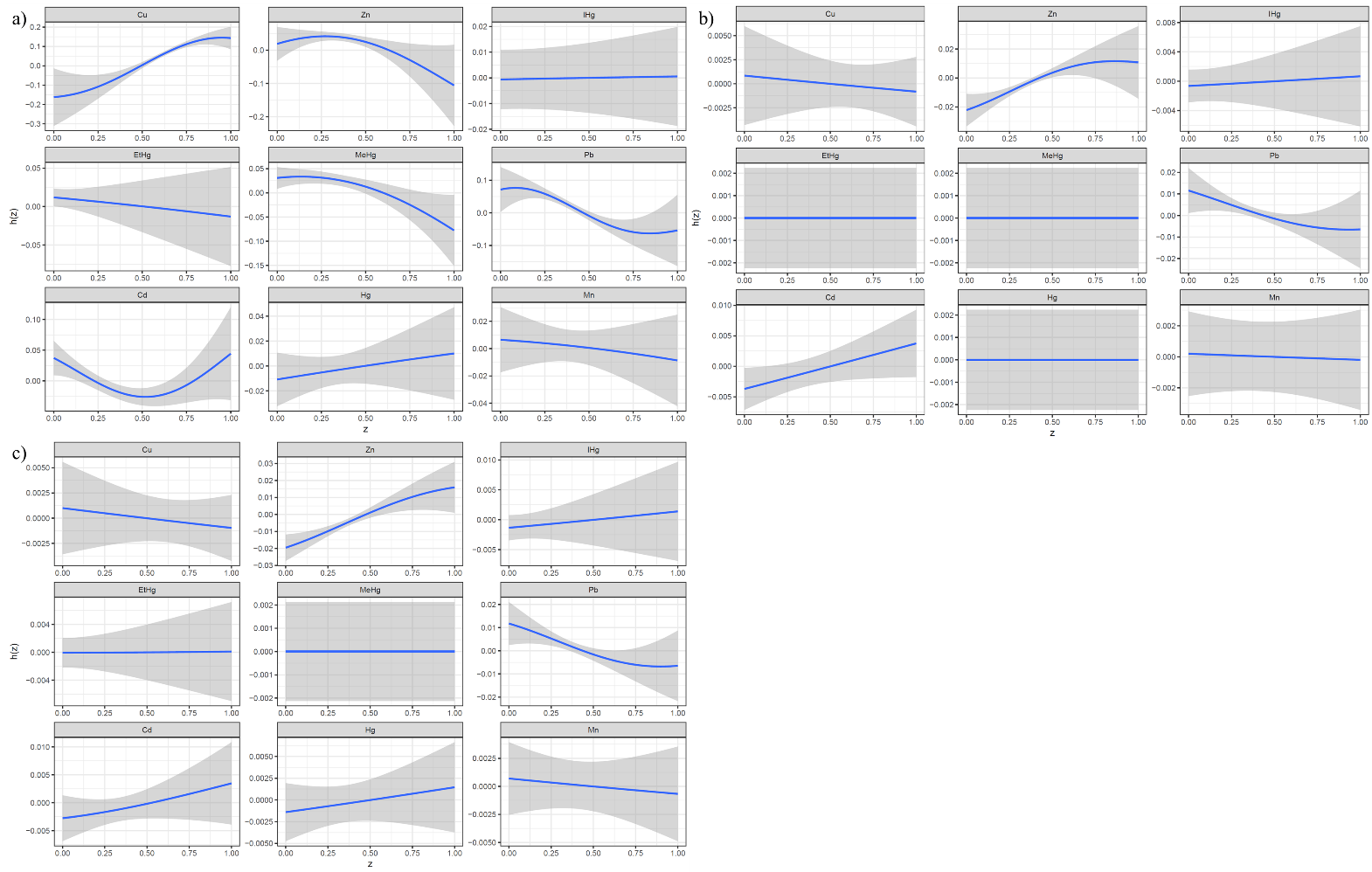


Figure S20 Univariate exposure–response function between each heavy metal and the TyGBMI when the other heavy metals were fixed at 50th percentiles.

*Note: a) was adjusted for age, gender, race, educational level, marital status and PIR; b) was adjusted for age, gender, race, educational level, marital status, PIR, weight, height, waist and BMI; c) was adjusted for age, gender, race, educational level, marital status, PIR, weight, height, waist, BMI, WBC, Lym, Mono, Neu, Eos and Baso.*


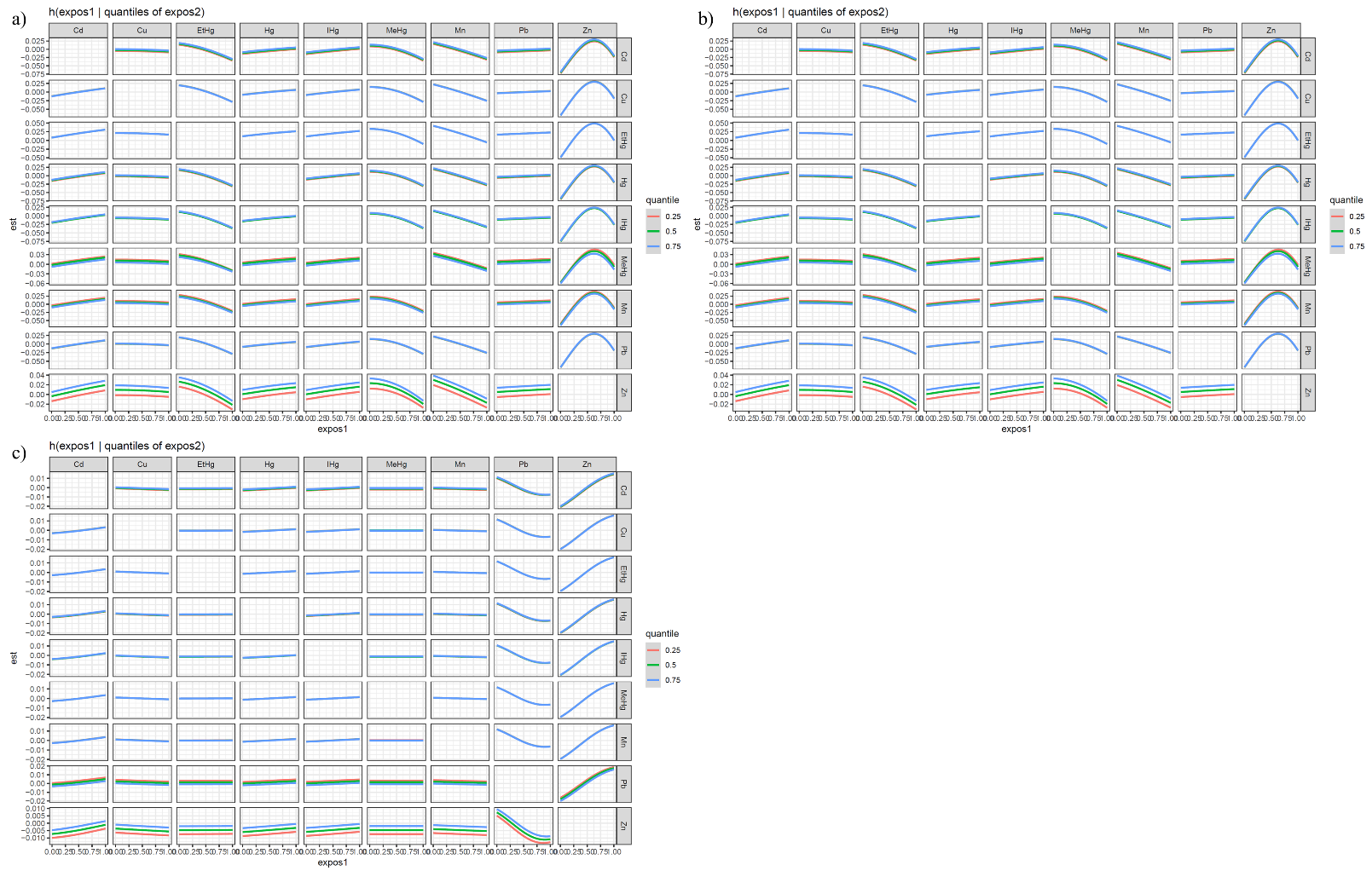


Figure S21 Single exposure-response functions for each heavy metal and the TyG when a single heavy metal was at the 75th compared with the 50th percentile and the concentrations of all the other heavy metals were fixed at either the 25th, 50th, 75th percentile in the BKMR model.

*Note: a) was adjusted for age, gender, race, educational level, marital status and PIR; b) was adjusted for age, gender, race, educational level, marital status, PIR, weight, height, waist and BMI; c) was adjusted for age, gender, race, educational level, marital status, PIR, weight, height, waist, BMI, WBC, Lym, Mono, Neu, Eos and Baso.*


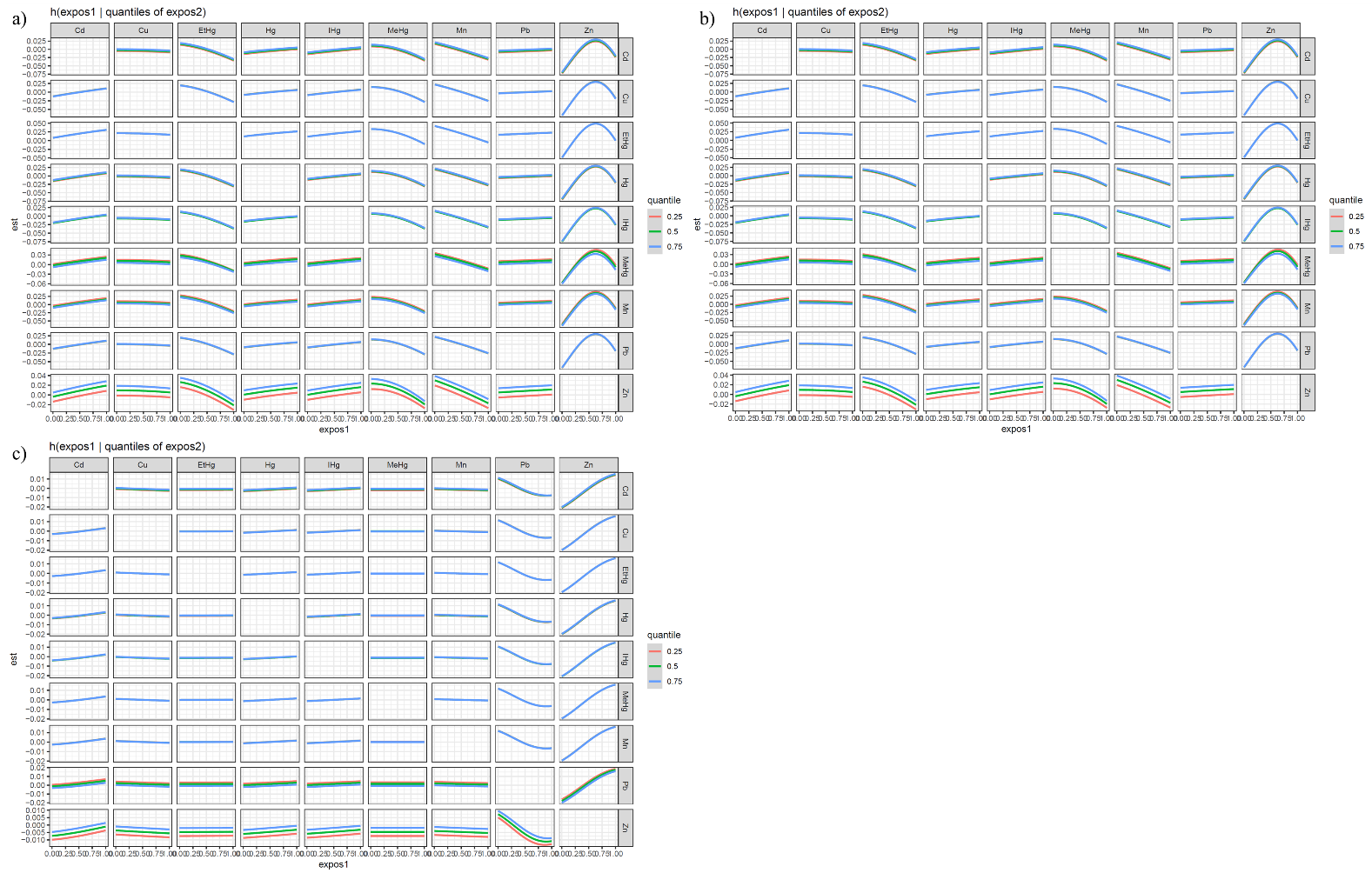


Figure S22 Single exposure-response functions for each heavy metal and the TyGWC when a single heavy metal was at the 75th compared with the 50th percentile and the concentrations of all the other heavy metals were fixed at either the 25th, 50th, 75th percentile in the BKMR model.

*Note: a) was adjusted for age, gender, race, educational level, marital status and PIR; b) was adjusted for age, gender, race, educational level, marital status, PIR, weight, height, waist and BMI; c) was adjusted for age, gender, race, educational level, marital status, PIR, weight, height, waist, BMI, WBC, Lym, Mono, Neu, Eos and Baso.*


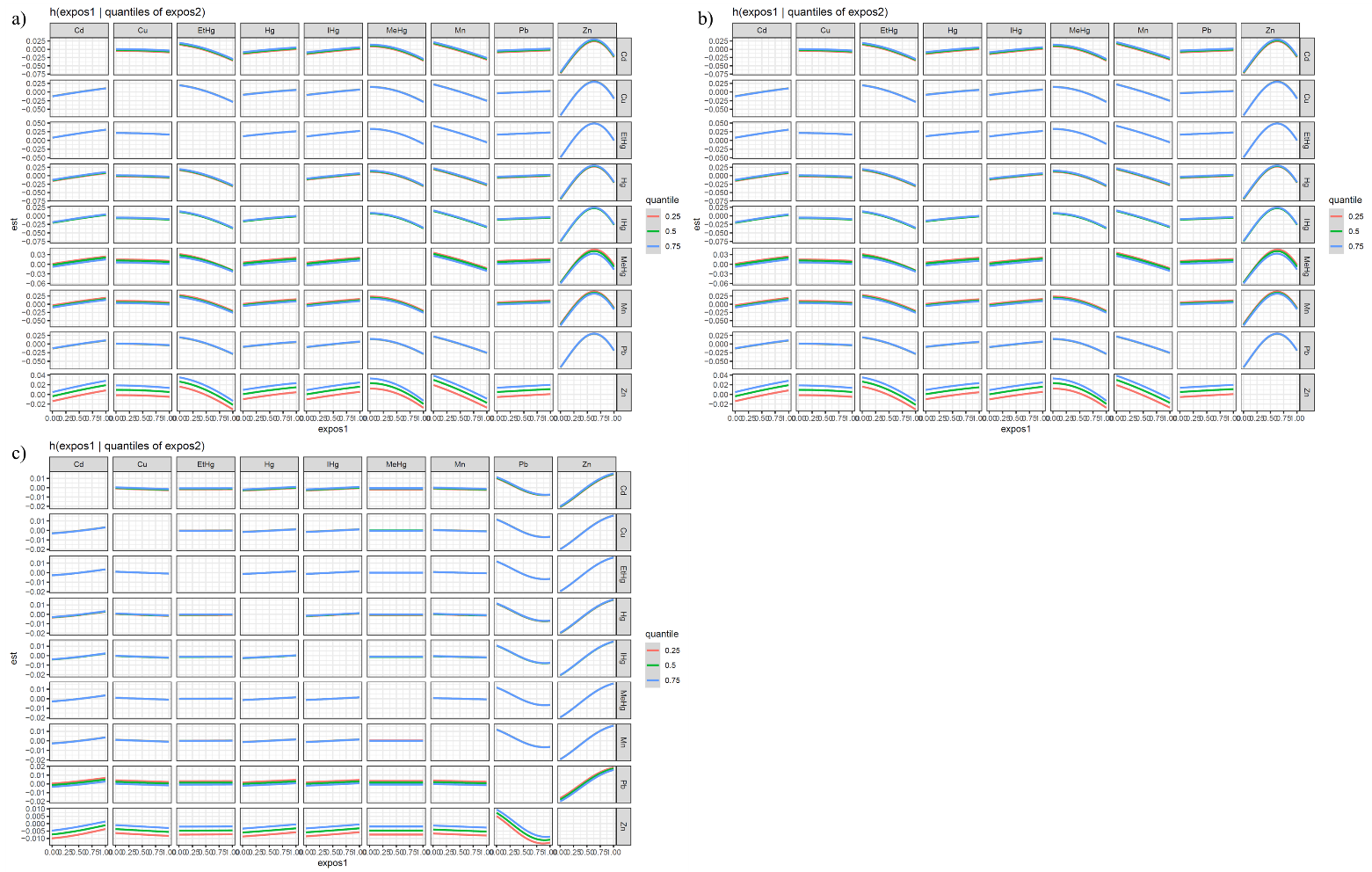


Figure S23 Single exposure-response functions for each heavy metal and the TyGWHtR when a single heavy metal was at the 75th compared with the 50th percentile and the concentrations of all the other heavy metals were fixed at either the 25th, 50th, 75th percentile in the BKMR model.

*Note: a) was adjusted for age, gender, race, educational level, marital status and PIR; b) was adjusted for age, gender, race, educational level, marital status, PIR, weight, height, waist and BMI; c) was adjusted for age, gender, race, educational level, marital status, PIR, weight, height, waist, BMI, WBC, Lym, Mono, Neu, Eos and Baso.*


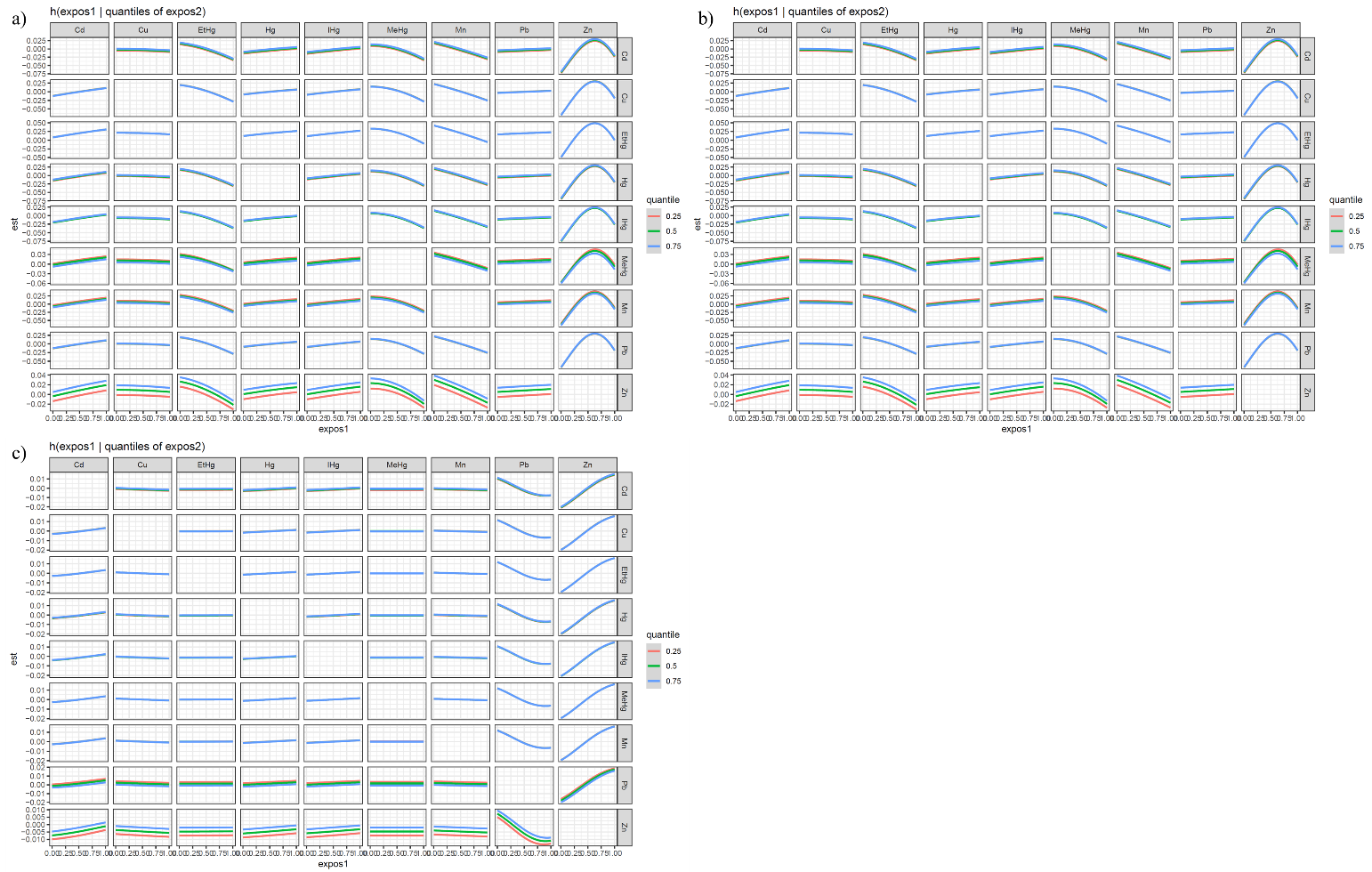


Figure S24 Single exposure-response functions for each heavy metal and the TyGBMI when a single heavy metal was at the 75th compared with the 50th percentile and the concentrations of all the other heavy metals were fixed at either the 25th, 50th, 75th percentile in the BKMR model.

*Note: a) was adjusted for age, gender, race, educational level, marital status and PIR; b) was adjusted for age, gender, race, educational level, marital status, PIR, weight, height, waist and BMI; c) was adjusted for age, gender, race, educational level, marital status, PIR, weight, height, waist, BMI, WBC, Lym, Mono, Neu, Eos and Baso.*


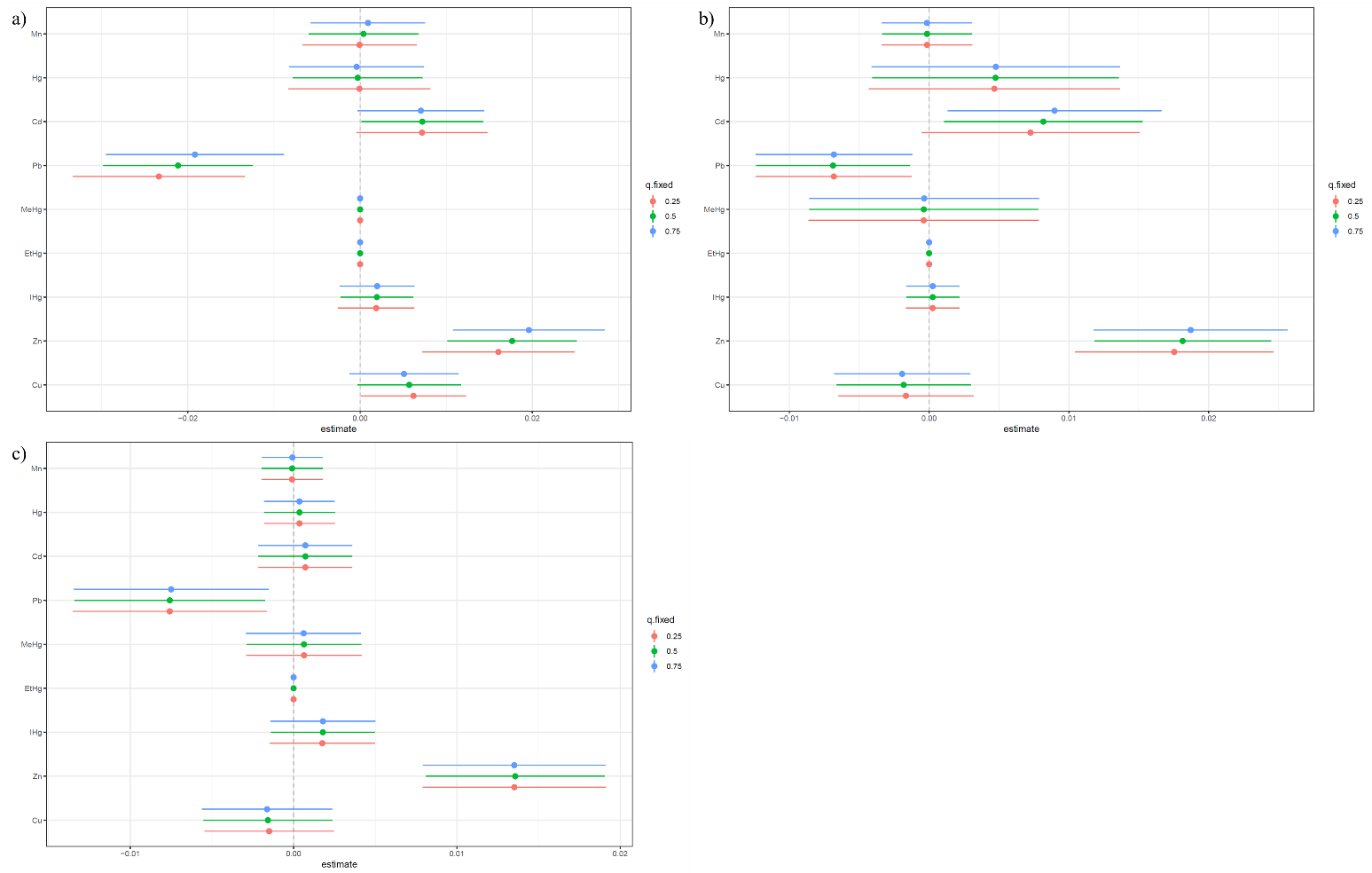


Figure S25 Bivariate exposure-response functions for each heavy metal and the TyG when one heavy metal was fixed at 25th, 50th, 75th percentiles and other heavy metals were fixed at the median in the BKMR model.

*Note: a) was adjusted for age, gender, race, educational level, marital status and PIR; b) was adjusted for age, gender, race, educational level, marital status, PIR, weight, height, waist and BMI; c) was adjusted for age, gender, race, educational level, marital status, PIR, weight, height, waist, BMI, WBC, Lym, Mono, Neu, Eos and Baso.*


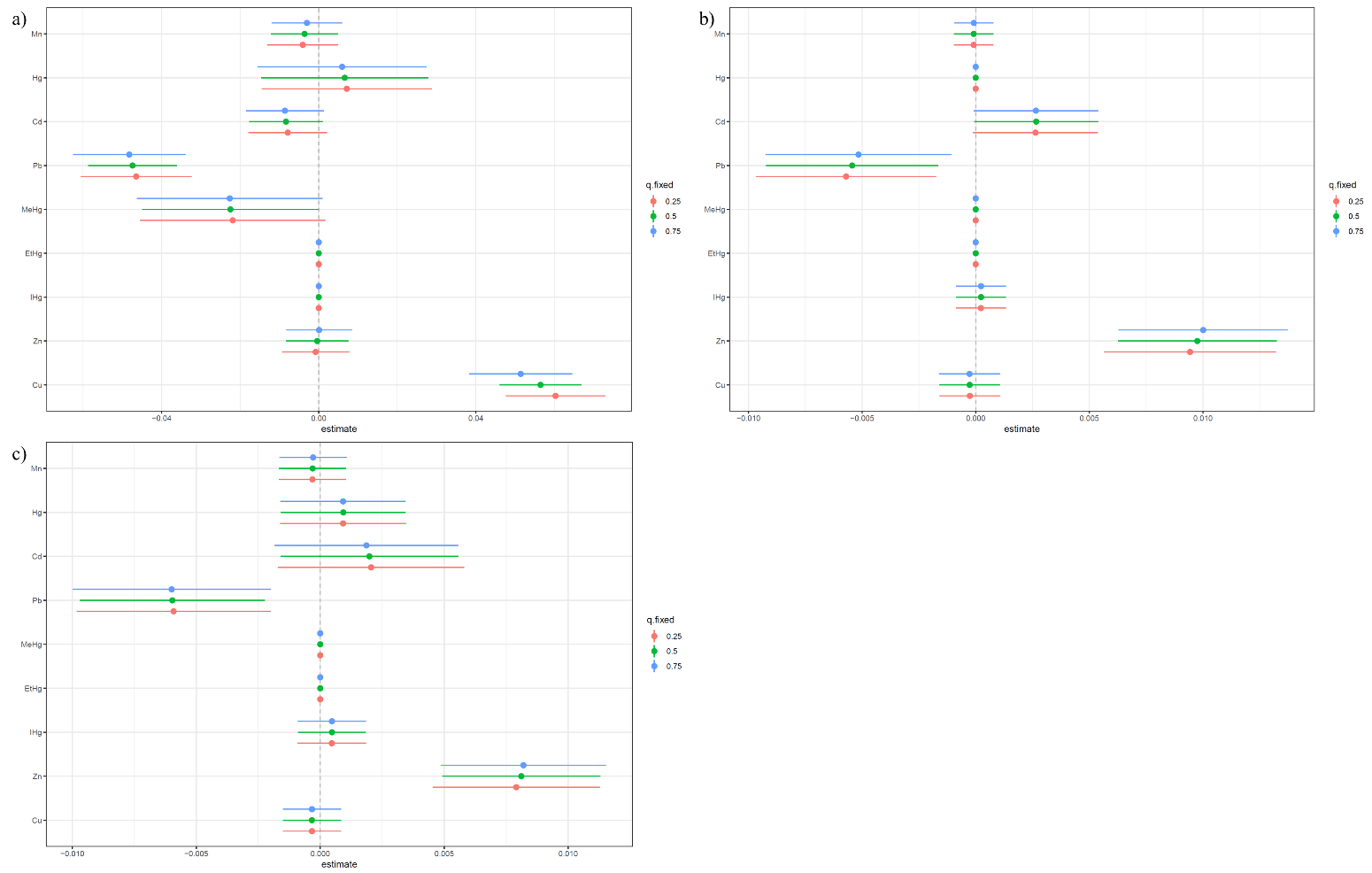


Figure S26 Bivariate exposure-response functions for each heavy metal and the TyGWC when one heavy metal was fixed at 25th, 50th, 75th percentiles and other heavy metals were fixed at the median in the BKMR model.

*Note: a) was adjusted for age, gender, race, educational level, marital status and PIR; b) was adjusted for age, gender, race, educational level, marital status, PIR, weight, height, waist and BMI; c) was adjusted for age, gender, race, educational level, marital status, PIR, weight, height, waist, BMI, WBC, Lym, Mono, Neu, Eos and Baso.*


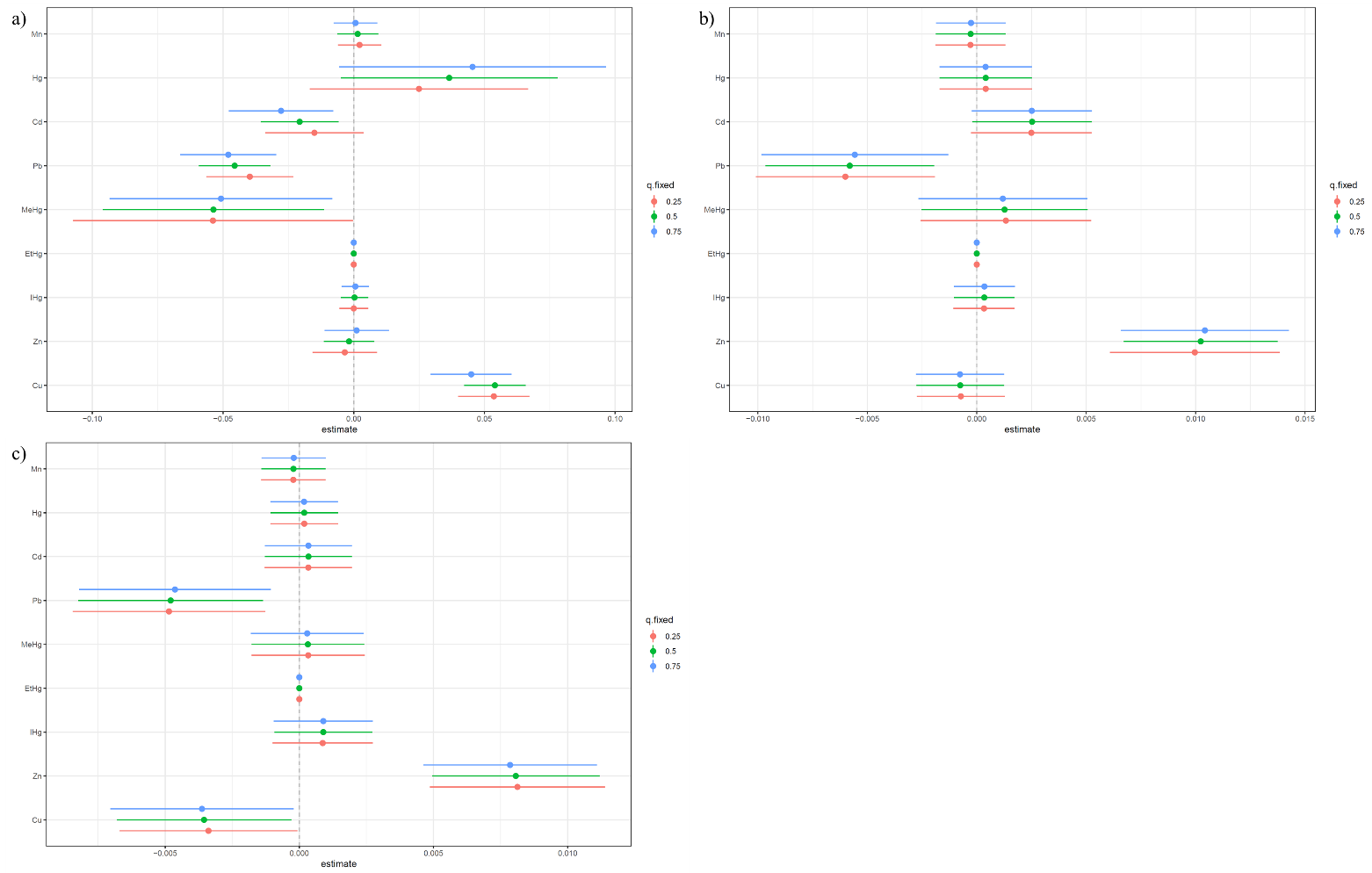


Figure S27 Bivariate exposure-response functions for each heavy metal and the TyGWHtR when one heavy metal was fixed at 25th, 50th, 75th percentiles and other heavy metals were fixed at the median in the BKMR model.

*Note: a) was adjusted for age, gender, race, educational level, marital status and PIR; b) was adjusted for age, gender, race, educational level, marital status, PIR, weight, height, waist and BMI; c) was adjusted for age, gender, race, educational level, marital status, PIR, weight, height, waist, BMI, WBC, Lym, Mono, Neu, Eos and Baso.*


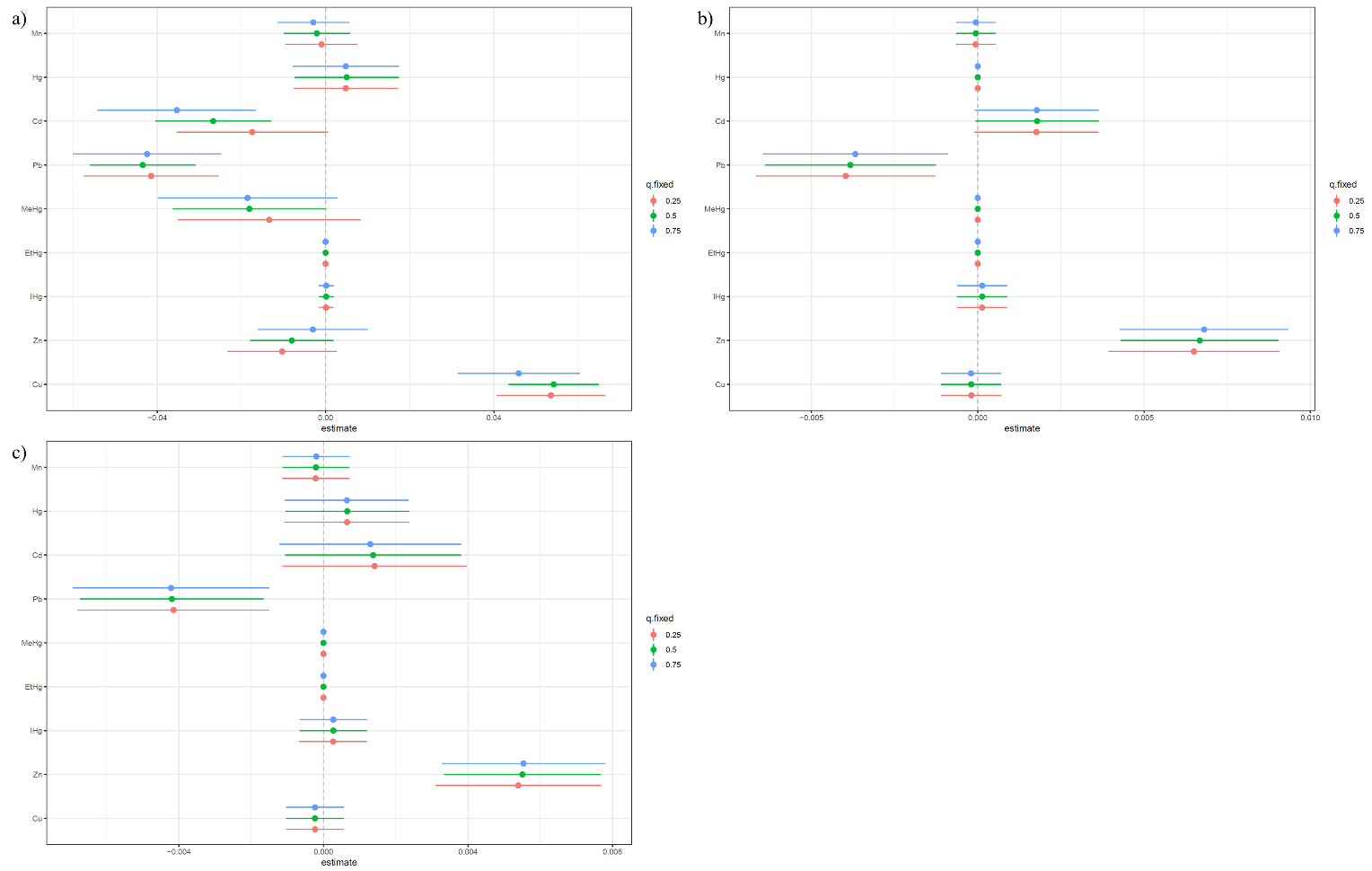


Figure S28 Bivariate exposure-response functions for each heavy metal and the TyGWC when one heavy metal was fixed at 25th, 50th, 75th percentiles and other heavy metals were fixed at the median in the BKMR model.

*Note: a) was adjusted for age, gender, race, educational level, marital status and PIR; b) was adjusted for age, gender, race, educational level, marital status, PIR, weight, height, waist and BMI; c) was adjusted for age, gender, race, educational level, marital status, PIR, weight, height, waist, BMI, WBC, Lym, Mono, Neu, Eos and Baso.*


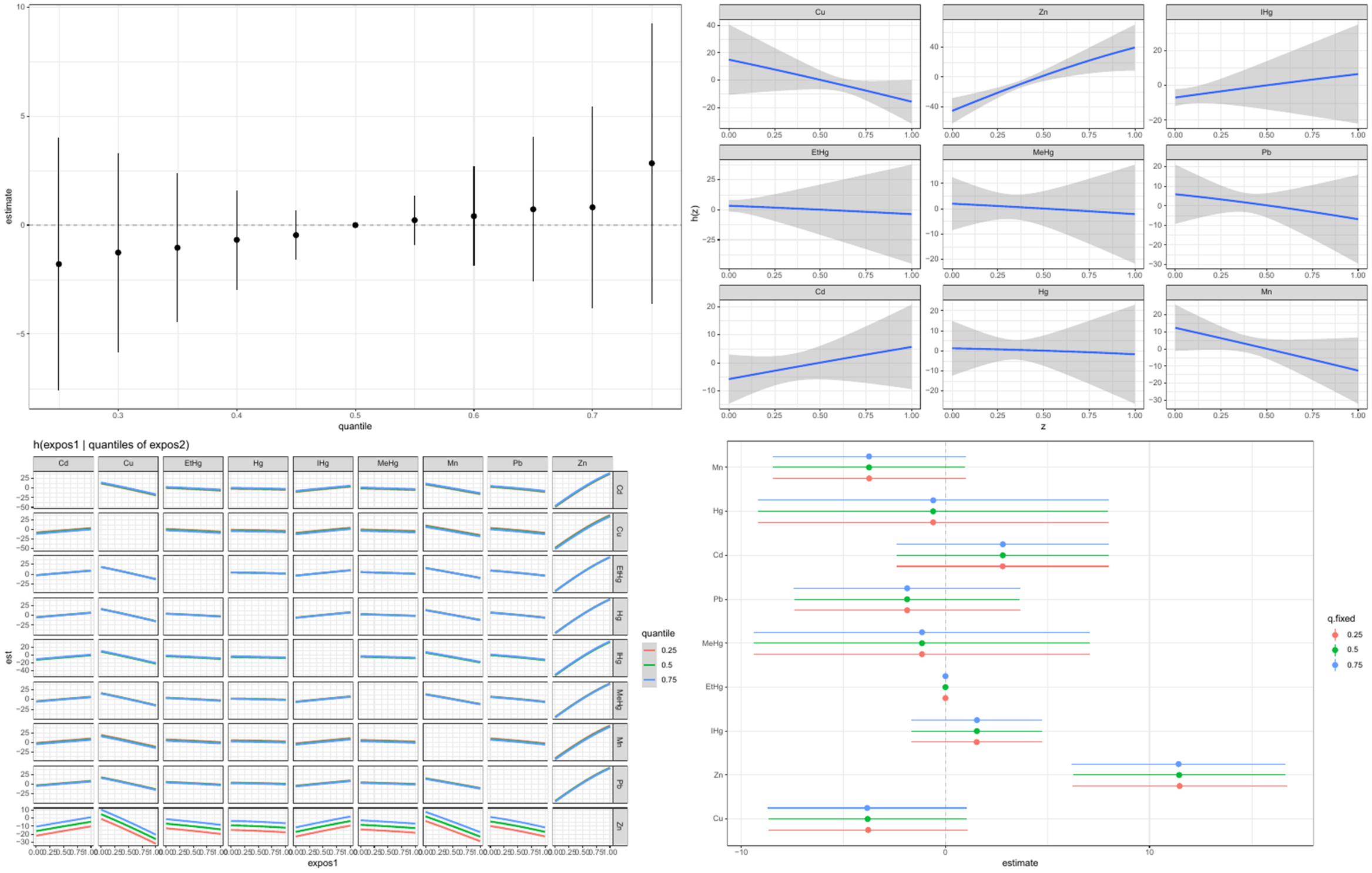


Figure S29 Association between Heavy Metal and TG


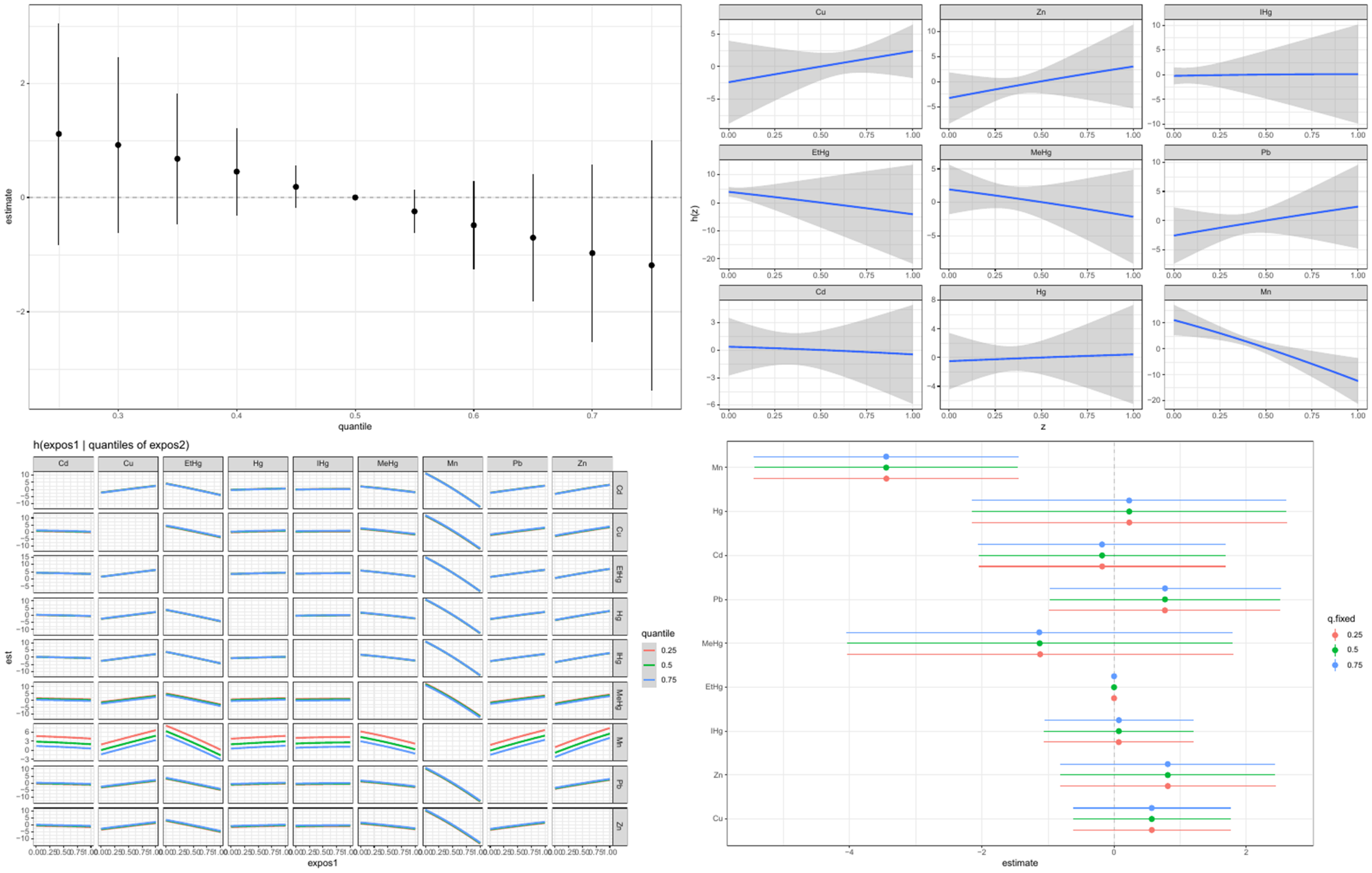


Figure S29 Association between Heavy Metal Exposure and BG
